# Can Dietary Supplements Be Linked to a Vegan Diet and Health Risk Modulation During Vegan Pregnancy, Infancy, and Early Childhood? The VedieS Study Protocol for an Explorative, Quantitative, Cross-Sectional Study

**DOI:** 10.3390/ijerph22081210

**Published:** 2025-07-31

**Authors:** Wolfgang Huber-Schneider, Karl-Heinz Wagner, Ingrid Kiefer

**Affiliations:** 1 Department of Nutritional Sciences, University of Vienna, Josef-Holaubek-Platz 2, 1090 Vienna, Austria; a00225229@unet.univie.ac.at; 2 Austrian Agency for Health and Food Safety GmbH, Spargelfeldstraße 191, 1220 Vienna, Austria; ingrid.kiefer@ages.at

**Keywords:** vegan diet supplements, childhood, pregnancy gynecologist, physician pediatrician, pharmacist, dietician

## Abstract

As veganism becomes more popular, the number of vegan pregnant women and children is steadily increasing. During vegan pregnancy and early childhood, there is a high risk for nutrient deficiencies that may impair child development. External factors, such as healthcare advice, social networks, and social environments, that affect the diet of vegan pregnant women, parents, and their children, as well as their approach towards dietary supplementation, have not yet been investigated. Various sources of information, combined with a lack of expertise, sparse food and nutritional health literacy, and qualitatively heterogeneous information provision by medical experts, unsettle vegan pregnant women and parents and affect their dietary choices and potentially the health of their children. The VedieS study aims to investigate potential connections between external influences and associated impacts on a vegan diet and the intake of dietary supplements (DS) of pregnant women and children. Two surveys are being conducted within the study: one targeting 1000 vegan pregnant women and parents, and another targeting 60 experts in each of five healthcare groups: gynecologists, pediatricians, general practitioners, pharmacists, and dietitians. This study is the first to examine how socio-economic, social, and further informational factors influence dietary practices during vegan pregnancy and childhood. It highlights the need for reliable, expert-led guidance, as current information sources are often inconsistent and may put these vulnerable groups at risk.

## 1. Introduction

During intensive development phases, such as pregnancy and childhood, the probability of insufficient nutrient supply rises [1]. In the course of a vegan diet, the focus should be on an adequate supply of vitamin B12, zinc, iron, calcium, vitamin D, iodide, and omega-3 fatty acids [2,3,4,5]. Nutrient deficiency can be prevented by supplying these critical nutrients of a vegan diet adequately, with specific foods or with DS [6,7,8]. A vegan diet is defined as a dietary pattern that excludes all animal-derived products—including meat, fish, dairy, eggs, and honey—and is based on the consumption of plant-derived foods such as fruits, vegetables, grains, legumes, nuts, and seeds [9]. A purely plant-based diet can provide all necessary macro- and micronutrients during pregnancy and childhood, except for a possible lack of docosahexaenoic acid (DHA) and eicosapentaenoic acid (EPA), as well as vitamin B12 [10]. Newborns of vegan mothers are on average smaller (small for gestational age) and are often born with a lower birth weight than those born from omnivores [11,12], which could lead to further nutrient-deficiency-related development and health impairments. If mothers lack an adequate supply of nutrients in their diet, nutrient deficiencies can occur in infants despite breastfeeding [13,14,15,16,17,18,19]. Expert groups such as the Academy of Nutrition and Dietetics (AND), the German Nutrition Society (DGE), and the Austrian Nutrition Society (OEGE) partly disagree about the risks of a purely plant-based diet for pregnant women, infants and children [20]. The AND considers a well-planned vegan diet to be safe, even during vulnerable stages such as pregnancy and childhood [21]. Although the DGE neither clearly supports nor rejects a vegan diet during the mentioned life phases, it points out insufficient data and the need for comprehensive nutritional skills for safe implementation [22]. The OEGE does not recommend a vegan diet during pregnancy and childhood due to the risks of possible malnutrition and possible associated impairments [23]. All organizations share the common point of view that a vegan diet during life phases such as pregnancy, infancy and childhood should be preceded by comprehensive planning with special emphasis on critical nutrients of a vegan diet—especially vitamin B12 supplementation. In the past no distinct statements were made on the benefits or drawbacks for vegan pregnant women and their children in terms of nutrient supply, due to a lack of studies with a sufficiently large number of participants or inadequate significance [24,25,26]. The latest findings show that there are risks of malnutrition, which can be prevented by the use of DS and observing the health and developmental status of mother and child [27,28]. Although the selection of vegan foods is becoming more diverse, there is no data on whether this expansion of what is on offer results in a better nutrient supply for vegans—the current state of studies suggests dietary supplementation is necessary [29,30,31]. Pregnant women who consume an omnivorous diet usually take DS to support the healthy development of their unborn children [32]. Influential factors that affect the approach of vegan pregnant women and parents towards DS have not yet been scientifically determined. It has been shown that mothers of vegan-fed children have less nutritional knowledge than mothers whose children follow an ovo-lacto-vegetarian diet [33]. Parents frequently ask pediatricians how to provide the most balanced diet for their children [34,35]. For this reason, constructive communication between parents of vegan-fed children and their pediatricians and dieticians is important to make comprehensive information about a healthy, purely plant-based diet available [36,37]. Despite frequent uncertainty about the safety of DS due to varying production standards regarding different origins and manufacturers, the market for supplements and botanicals is expanding [31]. Compared to pharmaceuticals, DS are subject to food law and are, therefore, liable to less strict controls [38]. An official approval process, that is required for pharmaceuticals and takes many years, is not obligatory for DS. This can have an impact on the safety and harmlessness of their consumption. Preparations that consumers buy online are often of inferior quality. Ingredients are inadequately declared and can even be detrimental to the health of consumers because of different regulations in different parts of the world, which is why customers and patients should be comprehensively informed by medical experts [39]. Additionally, adulterations can cause harmful health impacts [40]. Non-prescription DS compared to prescription DS are often characterized by insufficient information on ingredients, dosage and intake [41]. This represents a major risk, especially for pregnant women and children [42]. The relationship between vegan parents and especially physicians can, therefore, influence the health and well-being of vegan-fed children. However, the majority of pediatricians is perceived skeptical or negative towards a vegan diet, which can affect vegan parents’ trust in their children’s physicians and limit or prevent communication [34,43]. Many parents do not inform pediatricians about their children’s vegan diet in order to avoid being exposed to criticism [34]. A skeptical attitude of physicians could result from legitimate, nutrition-related objections on the one hand and from a lack of knowledge about a vegan diet and DS on the other [44]. This can lead to health impairments for vegan pregnant women and children due to a lack of information supply and miscommunication. It is not yet clear whether other specialists and medical experts, in addition to pediatricians, have a negative attitude towards a vegan diet during pregnancy and childhood and, therefore, offer little or no advice on vegan diets and DS. If a negative attitude also exists among gynecologists, general practitioners, dieticians and pharmacists, this could have a negative health- and nutrition related effect on vegans due to a lack of information transfer. The level of information and the associated information provided by medical experts on a vegan diet and DS during pregnancy and childhood, therefore, has a significant impact on the adequate nutrient supply of vegans. The level of knowledge among medical experts regarding a vegan diet and DS has not yet been sufficiently examined. Competent advice on supplementation with DS is essential for a healthy development during pregnancy and childhood. If information is not provided by medical experts, vegans use other sources of information, particularly social media. It can be assumed that many vegans are insufficiently, incorrectly or not at all informed about DS and a well-balanced vegan diet due to the different quality of disposable information. Dubious sources of information and influential factors (e.g., social environment, internet and social media, magazines, advertisement) are likely to make parents feel insecure and can affect the health of their children. A lack of expertise on the part of vegan pregnant women and parents (on a vegan diet and DS) and insufficient support from medical experts (pediatricians, gynecologists, general practitioners, pharmacists, dieticians) entail possible health risks. The impact of medical expertise and other social, sociodemographic factors (e.g., age, social environment and background, internet and social media, advertising), on the diet of vegan pregnant women and children (up to the age of 5 years), as well as on supplementation with DS needs to be investigated. Whether and to what extent external factors influence a vegan pregnancy, infancy and childhood have not yet been comprehensively investigated. In addition, there is still no data on the opinion and level of knowledge of consulting medical experts and associated consequences regarding nutrition and the possibility of supplementing DS for vegans.

Previous studies that investigated vegan diets during pregnancy and early childhood focused either on medical risks or aspects such as parents’ knowledge about a vegan diet or DS intake. Kostecka et al. [33] found that parental food and nutritional health literacy plays a central role in ensuring safe vegetarian diet for children, especially regarding the correct use of DS. Palma et al. [5], Sebastiani et al. [7] and Papadopoulou et al. [45] show that a well-planned vegan diet can be suitable during pregnancy. However, careful medical monitoring of micronutrient intake is necessary, which highlights the importance of advice from medical experts. Nevertheless, these studies do not fully consider the social and behavioral factors that can influence parental decisions. The VedieS study addresses these gaps by investigating how external factors (e.g., criticism from medical experts, lack of advice, use of non-professional information sources) influence dietary behavior and DS use among pregnant vegans and parents of vegan children. A study by Bivi et al. [34] showed that many vegan parents reported that they do not feel sufficiently supported by their pediatricians. Instead, many relied on online platforms (e.g., social media) or advice from peer groups to decide whether to take or administer DS. The VedieS study aims to examine how factors such as trust in expert advice, perceived barriers regarding effectiveness of DS, and social expectations can influence vegans’ adherence to supplementation recommendations and maintenance of their diet during sensitive life stages such as pregnancy and childhood. Furthermore, little is known about medical experts’ perspective on this issue. A recent study from France [46] found that many pediatricians and dieticians feel unprepared to advise vegan families. Some even have negative attitudes toward a vegan diet. By including both vegan participants and medical experts in our survey, we want to examine whether such attitudes lead to less effective counseling, inconsistent advice, lower patient trust and consequently to possible health risks. Understanding these connections is important to improve communication and to provide reliable, competent support for vegan parents and pregnant women. Previous research has primarily focused on food and nutritional health literacy or the medical impact of a vegan diet. Our study is one of the first to examine how social environment, information sources, and trust in medical experts influence DS use and dietary behavior in vegan families during pregnancy and early childhood. The findings can help explain behavior patterns and support medical experts in their advisory role.

The VedieS study is based on several research hypotheses that examine how external factors influence the dietary behavior of vegans during pregnancy and early childhood. We hypothesize that pregnant vegans and parents of vegan-fed children often seek information and support from other vegans, peer groups, or social media but not from medical experts. This is especially important when medical advice is lacking or perceived negatively towards a vegan diet. We also hypothesize that critical or unclear advice from medical experts may lead to uncertainty, fear of nutritional deficiencies, or the use of unreliable information sources. In some cases, this could result in dietary changes or incorrect use (or even avoidance) of DS. Another hypothesis is that inadequate communication about the use of DS, particularly about the administration of DS to children (e.g., dosage, dosage form), may lead to inconsistent use or misunderstandings, thus increasing the risk of health risks. It is also assumed that many vegan parents have difficulties to clearly distinguish between DS, food, and medication—especially when they receive unclear advice from medical experts or insufficient advice. Finally, while many vegans consider their diet to be generally healthy, they also recognize the importance of DS during pregnancy and early childhood. However, the decision to take DS may be more influenced by social or emotional factors than by scientific reasons. These hypotheses form the basis of the study and shape the development of the survey, the research questions and the planned analysis.

### Theoretical Framework

**Figure 1:**
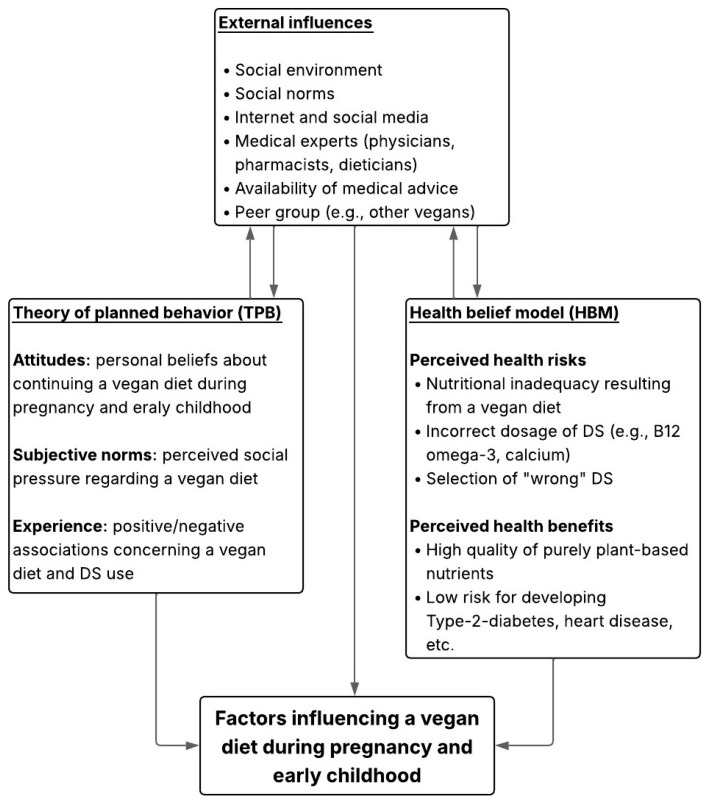
Factors influencing a vegan diet during pregnancy and early childhood, based on TPB, HBM, and external influences.

The theoretical framework of the VedieS study is based on two models from health psychology: it combines the Health Belief Model (HBM) and the Theory of Planned Behavior (TPB), as shown in Figure 1. These models can help to explain how pregnant vegans and parents of vegan-fed children make decisions about their diet and DS use during pregnancy and early childhood and can help to understand how views and communication of medical experts can influence their decisions. The HBM focuses on individuals’ perceptions of health risks (e.g., nutrient deficiencies of vitamin B12 or calcium), as well as the perceived benefits and barriers related to DS intake. For instance, if vegans feel criticized or not adequately supported by medical experts, they may lose confidence in their advice and instead turn to less reliable sources, such as social media or peer groups (e.g., other vegans, friends). Studies have shown that the HBM can help to predict behavior related to diet and DS use, particularly among pregnant women [47,48]. The TPB describes the factors that shape people’s intentions to act. It considers their attitudes toward DS, the influence of others (e.g., family, friends, medical experts), and whether they feel capable of managing their diet and supplement use. This becomes especially relevant when vegans are seeking trustworthy dietary advice. TPB has been used in previous research to explain motivations for plant-based diets and supplement intake [47,49]. For these reasons, both models served as the basis for designing the VedieS study and developing the questionnaires. Items for both groups (vegans and medical experts) were grounded in the main principles of HBM and TPB, which include perceptions of risks and benefits, social influences (e.g., pressure, support), and confidence in managing DS-related decisions (e.g., dosage, administration). This approach is supported by recent studies [33,34,46], which highlight the importance of trust, attitudes, and access to comprehensive information among vegans and vegetarians. These findings provide further justification for using the HBM and TPB as the theoretical framework for the VedieS study.

## 2. Materials and Methods

### 2.1. Objective

The aim of the descriptive, cross-sectional VedieS study is to investigate, for the first time, the factors influencing vegan parents and pregnant women in adhering to a strictly plant-based diet and in their use of DS, as well as the potential implications for their health and the health of their children up to five years of age. External factors that lead to and influence the development of individual food and nutritional health literacy and the use of DS by vegans are the main research questions. Hence, this approach focuses on the relevant information sources for vegans as the main cause of their food and nutritional health literacy. Not only vegans themselves but also medical experts (pediatricians, gynecologists, general practitioners, pharmacists, dietitians) are being asked about their opinion, knowledge and advice on a vegan diet, to be able to draw conclusions about the quality of information they provide to vegan parents and pregnant women.

Influential factors, sources of information gathering, reasons to choose certain DS, difficulties in administering DS to vegan-fed children (up to the age of 5 years), as well as details about the compliance of DS intake of vegan pregnant women and parents, should be revealed. The results can serve as a starting point for comprehensive information provision for vegans and provide insight into their food and nutritional health literacy. The opinion of medical experts has a decisive influence on the health of vegans. For this reason, the content of their advice will also be assessed as the advisory role of the medical experts is essential to provide vegans with reliable information on a balanced vegan diet and use of DS.

Current medical advisory conditions and sources of information for vegans are being examined in detail. The obtained results can indicate the importance of comprehensive advice from medical experts and the prevention of health risks for pregnant vegans and parents of vegan-fed children. Therefore, the study aims to highlight that a balanced, purely plant-based diet for pregnant women and children depends mainly on advice from physicians, pharmacists and dieticians. A comprehensive specialist knowledge about a vegan diet and DS among medical experts is the basic prerequisite for competent information provision and as a result essential for a low-risk vegan pregnancy and childhood. The study results are intended to encourage medical experts to provide comprehensive advice to vegans to be able to support pregnant women and parents of vegan-fed children as best as possible during these vulnerable life stages. As the number of vegans is constantly increasing, expertise about a vegan diet and DS should be expanded among physicians, pharmacists and dieticians and made permanently available.

For this reason, the following objectives were defined:

#### 2.1.1. Primary Objectives

##### Determination of Main Influential Factors That Affect Vegans Regarding Their Diet During Pregnancy and When Feeding Their Children (Up to the Age of 5 Years)

Influence by medical experts or other influences (e.g., social environment, Internet and social media, magazines) concerning the decision for or against a vegan diet should be clarified. Depending on the source of information, the possible risks of a vegan diet during pregnancy, infancy and early childhood can be misjudged.

The identification of information sources for vegans on dietary supplements, as a reason for the approval or rejection of DS (during pregnancy, infancy and early childhood), embodies trustworthiness by ranking.

It should be determined which information on DS (during pregnancy, infancy and early childhood) is used and whether this information appears trustworthy or causes uncertainty. Information received affects selection, intake, or avoidance of certain DS. The health impact of selected sources of information on DS can be made visible. Medical expert advice and other opinion-forming aspects (e.g., social environment, Internet and social media, magazines) should be ranked according to their relevance. Main sources of information should be identified.

This can be an important approach for the improvement of information supply on DS in a vegan diet and for minimizing risks preventively. By recognizing sources of information classified as the most important for vegans, the main causes of frequent inadequacies in the provision of information could be identified and if necessary, positively changed.

##### Opinions and General Knowledge of Medical Experts About a Vegan Diet and Dietary Supplements (For a Vegan Pregnancy, Infancy and Early Childhood) Should Be Established

The impact of expert opinion and knowledge on a vegan diet regarding their advice on DS for vegans (during vegan pregnancy, infancy and early childhood) should be clarified. Statistically evaluated results of the survey should allow conclusions about the influence of information supply by medical experts on nutrient supply, of vegan pregnant women and growing children (up to the age of 5 years). A possible misinterpretation of the health risk of a vegan diet during pregnancy, infancy and early childhood due to insufficient knowledge of DS could increase health risks.

A negative basic attitude towards a vegan diet influences the relationship of trust between vegans and their physicians. If there is no advice on DS, health risks can increase.

#### 2.1.2. Secondary Objectives

##### Determination of the Level of Knowledge of Vegans on Dietary Supplements, Nutrient Deficiency Risks and Correct Dosage of Dietary Supplements During Pregnancy, Infancy and Early Childhood (Up to the Age of 5 Years)

If the risk of incorrect dosage of DS is misjudged by vegans during pregnancy, infancy and early childhood, nutrient deficiencies or overdose with DS become likely. Risks of nutrient deficiency due to missing intake or incorrect selection of DS because of a lack of food and nutritional health literacy should also be cognizable. Avoiding these circumstances would require a competent information provision for vegans by medical experts (pediatricians, gynecologists, general practitioners, pharmacists, dieticians). Imprecise advice could affect the relationship of trust between medical experts and vegans as well as the nutrient supply during pregnancy, infancy and early childhood.

##### Investigation of Conceptualization of Dietary Supplements, with a Special Focus on Their Contribution to Health Maintenance and Optimization from Vegans’ Perspective

The perception of what a DS can achieve, whether it is comparable to medicine or food, or whether a positive/negative effect can be expected, depends on the level of knowledge of vegans about DS. How vegans perceive DS and how they rate their effectiveness influences their intake behavior. What health means for vegans and whether DS are perceived as health-promoting from a vegan perspective should also be clarified.

Determining what is meant by health optimization for vegans creates an understanding of the intake or rejection of DS. Rejection of DS due to health concerns can indicate insufficient education about health benefits.

##### Determination of Compliance with Dietary Supplements During Pregnancy, Infancy and Early Childhood as Well as the Consequences of Administration Difficulties to Vegan-Fed Children (Up to the Age of 5 Years)

It will be investigated whether vegans consider regular intake or administration of DS during pregnancy, infancy and early childhood to be effective. In addition, it should be clarified whether certain dosage forms (e.g., drops, pills, capsules) of existing DS for infants and children represent an unsettling factor for vegan parents, which in turn increases the risk of incorrect dosage and resulting nutrient deficiencies (e.g., wrong administration, wrong dosage). Medical experts should also evaluate the options of dosage forms and compliance as well as the consistency of administration by parents to their vegan-fed child. This should clarify whether dosage forms of DS can have an impact on the regularity of administration and subsequently on the development of nutrient deficiencies. Statements about the regularity, duration of intake and compliance of DS are intended to allow conclusions about the supply of consumers with the corresponding DS and to show any potential dangers or health-enhancing effects.

If vegans indicate a high level of compliance when taking DS, the willingness to take DS is obvious and the relevance of competent expert advice on DS can be strengthened.

##### Investigate if Gender-Specific and Socio-Demographic Differences Within the Group of Vegans Exist

Different perceptions of and opinions on a vegan diet and DS (during pregnancy, infancy and early childhood) between vegan women, men, diverse people as well as between vegans in and outside the city should clarify differences in nutrient deficiency-associated risks of a vegan diet and the intake of DS. Other socio-demographic characteristics such as age, education, income, and employment status should also be included. Risk deviations could exist due to unequal access to information.

The goals of the VedieS study are to

- Illustrate the importance and necessity of comprehensive advice on DS from medical experts;
- Reduce the risk of malnutrition of vegans by encouraging physicians, pharmacists and dieticians to provide competent advice;
- Promote further training on the knowledge of a vegan diet and DS among medical experts;
- Increase the willingness to optimize the food and nutritional health literacy of pregnant vegans and parents of vegan-fed children and raise their awareness of its importance.

### 2.2. Study Design

The VedieS study is an explorative, cross-sectional study. Two quantitative online surveys on a vegan diet and the intake of DS during pregnancy, infancy and early childhood (up to the age of 5 years) are carried out: The first survey focuses on vegans (parents and pregnant women) and the second survey on medical experts (pediatricians, gynecologists, general practitioners, pharmacists, dieticians). Each survey will take approximately 20–25 min to answer.

Recruitment for the survey is scheduled to take place between October 2023 and the end of July 2025. There is no survey restriction to Austria. Participants from Germany, Switzerland and Italy/South Tyrol, Liechtenstein, and Luxembourg (or other EU countries) can also answer the questionnaires and will be included in the evaluations. Since the survey is conducted in German, the survey refers to participants who understand the German language.

### 2.3. Participants

Vegans are recruited by public announcements, newsletters and social media (Facebook, Instagram) of cooperating specialized organizations and medical experts that forward survey announcements, posters, and flyers, as well as info sheets, to potential participants.

Participants will be forwarded an online link/URL to the anonymous questionnaire. Before they can start the survey, the topic and further details of the survey are explained in an introduction. Potential participants interested in the study will have the immediate possibility to participate in the online survey wherever they are.

Medical experts (pediatricians, gynecologists, general practitioners, pharmacists, dieticians) are recruited by being contacted via email and through the support of professional organizations, info sheets, notices in hospitals, medical offices, pharmacies, and universities. Participants interested in the study will have the immediate possibility to participate in the (online) survey via an (online) link/URL.

The questions asked within the questionnaire determine whether participants are eligible for the survey or are excluded (inclusion/exclusion criteria).

More than 15 organizations forward/forwarded the survey via their newsletter and/or social media.

### 2.4. Eligibility Criteria

Inclusion criteria for vegans:

1. Females/diverse participants (ages 18 and over) who follow/followed a vegan diet during their pregnancy/pregnancies (if there is/was more than one pregnancy, at least during one of their pregnancies).
2. Parents (female/male/diverse—ages 18 and over) who feed/fed their child/children (if there is more than one child, at least one of them) a vegan diet at least between the ages of 0 and 5 years.
3. Vegan mothers/diverse participants (ages 18 and over) who breastfeed/breastfed their infant(s)/child(ren). Answering questions regarding their infant(s)/child(ren) up to the age of 5 years will be included.
4. Pregnant and breastfeeding women/gender diverse participants/parents (f/m/d) who make rare exceptions to their vegan diet and a vegan diet of their child(ren)—up to the age of 5 years. Inclusion in the survey depends on the frequency of exceptions → determined through questions/given answers within the survey. Participants that consume animal products a maximum of twice a month will be included; hence, participants who do not make vegetarian, pescetarian, or omnivorous exceptions more than twice a month will be included.
5. Confirmation of the participant information (online—addressing the subjects, purpose and process of the study, opportunities for discussion of further questions, duration of the questionnaire, who is conducting the study, possibility for further inquiries).

Exclusion criteria for vegans:

1. Females/diverse participants who change/changed their vegan diet (eat/ate a vegetarian or omnivorous diet) during their pregnancy/pregnancies.
2. Parents who do not feed/have never fed their children a vegan diet at least up to the age of 5 years (if vegan mothers followed a vegan diet during their pregnancy, exclusion only relates to questions concerning their children).
3. No confirmation of the participant information (online).

For inclusion in the study, at least one inclusion criterion (plus participant information) must be fulfilled. Exclusion (or not inclusion) will happen if only exclusion 3 is fulfilled. If only 1 or 2 is fulfilled, and 3 is not, participants will be included in the survey.

Inclusion criteria for medical experts:

1. Gynecologists, pediatricians, general practitioners, pharmacists, and dieticians with or without a consulting focus on vegans.
2. Confirmation of the participant information (online).

Exclusion criteria for medical experts:

1. Other medical specialists not mentioned in the inclusion criteria.
2. No confirmation of the participant information.

For inclusion in the study, all criteria must be fulfilled. Exclusion (or non-inclusion) will happen if only one exclusion criterion is fulfilled.

All participants have to understand the German language in order to answer the questionnaire.

### 2.5. Questionnaires

Participants are forwarded the scientific survey via a link/URL. The questionnaires can be answered at any time and from any chosen location via LimeSurvey [50] (LimeSurvey Cloud Version 6.6.7) using a tablet, PC, laptop, or smartphone. The questionnaires used in this study are provided in the Supplementary Materials. The survey tools were developed specifically for this research question and are grounded in the ideas of the Health Belief Model (HBM) and the Theory of Planned Behavior (TPB). These theoretical models served as a basis to define research questions and hypotheses and to develop questionnaire items. Two online surveys were developed: one for vegan participants (pregnant women and parents of vegan-fed children) and one for medical experts (physicians, pharmacists, and dieticians). All participants in each target group received the same version of the respective questionnaire. Within the survey for vegan participants, there are 10 question categories and corresponding detailed questions on the following: health (A), definition and reasons for a vegan diet (B), definition and understanding of and about DS (C), benefits, risks and expectations of DS (D), personal selection criteria of DS (E), level of knowledge about DS (F), sources of information about a vegan diet and DS (G), influential factors regarding a vegan diet and DS (H), deficiency and dosage of DS (I), and administration and compliance with DS intake (J). The questionnaire categories for the medical experts are structured similarly, but with a focus on topics related to patient and client counseling, as well as the personal views of physicians, pharmacists, and dietitians regarding a vegan diet and the intake of DS during pregnancy and childhood. Both questionnaires contain Likert scales, multiple-choice and single-choice questions, ranking questions, and an open-ended item. While most of the used response formats are common in surveys, the actual items were newly developed for the VedieS study. Because the questionnaires were developed based on the theoretical framework and objectives of the study itself, no standardized or pre-validated instruments were used, so no external approval was required. No additional statistical validation methods (such as factor analysis) were planned, as the questionnaires were not designed as psychometric tests but as data collection instruments based on the study hypotheses. To ensure content validity, a pre-test was conducted. The questionnaire was reviewed by nutritional experts, and a pretest was conducted with individuals from the target groups (vegans *n* = [20.0], medical experts *n* = [10.0]). This pretest focused on comprehension, relevance, and completion time of the items. Due to the received feedback, minor modifications for improvement were made. On average, completing the questionnaire took approximately 25 min for vegan participants and 20 min for medical experts.

### 2.6. Calculations of Case Number Scenarios

#### 2.6.1. Sample Size

This study does not follow a hypothesis-testing design with predefined effect sizes or intervention outcomes. Therefore, no formal power calculation (e.g., based on alpha level, statistical power, or expected effect size) was carried out. Instead, the sample size was determined based on population estimates, feasibility, and the exploratory character of the study.

##### Planned Sample Size for Vegan Participants

The study targets a small and hard-to-reach group whose exact size and characteristics are difficult to determine: individuals who follow a vegan diet during pregnancy or while raising their children. Although estimates suggest that 0.5–1% of the Austrian population follows a vegan diet [51], there are no official data on how many continue this diet throughout pregnancy and early parenthood. It is assumed that some individuals who usually follow a vegan diet may change their diet during these life stages, often due to concerns about nutrient adequacy, limited medical counseling, or external pressures. While exact numbers are unavailable, studies suggest that such factors may lead some vegans to modify or discontinue their diet in pregnancy or because of their children [33,52]. This uncertainty makes it particularly difficult to determine the actual size of the relevant study population. According to national birth statistics, around 400,000 children are expected to be born in Austria over a five-year period [53]. If 0.5–1% of those children are raised in vegan households, this would correspond to an estimated 2000–4000 families. The study aims to reach approximately 25% of this estimated population, resulting in a target sample of 1000 vegan participants. This sample size allows for meaningful descriptive analyses and subgroup comparisons (e.g., supplement users vs. non-users), without implying statistical representativeness.

##### Planned Sample Size for Medical Experts

For medical experts, the aim is to recruit approximately 60 participants per expert group (pediatrics, gynecology, general medicine, pharmacy, dietetics). This number was chosen because of similar studies, along with what is realistically possible, and the range of opinions expected across different medical professions [35,54,55].

Feedback from the pretest and preparation phase showed that many medical experts feel they have little or no experience with vegan patients—especially pregnant individuals or families with vegan-fed children. Hence, some may not see the topic as relevant to their work, which can affect their willingness to participate. Still, gathering a broad range of perspectives (including those who feel unsure or not directly involved) is important for the goals of the VedieS study. The planned sample size is large enough to examine differences in counseling practices, knowledge, and attitudes both within and between the professional groups.

### 2.7. Statistical Methods and Data Analysis

The survey data will first be exported from LimeSurvey to Excel (Microsoft Corp., Redmond, WA, USA) and then analyzed using IBM SPSS Statistics version 31 (IBM Corp., Armonk, NY, USA) and R version 4.5.1 (R Core Team). Descriptive statistics will be used to summarize the results. For example, averages and standard deviations will be calculated for numeric variables, while frequencies and percentages will be reported for categorical data. Diagrams such as bar charts and boxplots will visualize the results. Depending on the type of data, different methods will be used to compare groups. Categorical variables will be analyzed using chi-square tests, and continuous variables with ANOVA. For yes/no outcomes (such as whether participants use DS or whether medical experts recommend them) logistic regression models will be applied to examine possible influencing factors. We plan to use both simple (univariate) and more complex (multivariate) logistic regression models. In the multivariate models, we will include relevant background characteristics like age, gender, education, household situation, or medical profession to better account for possible confounding effects. Since the study is exploratory, *p*-values will be reported descriptively. There will be no correction for multiple testing. Comparisons between groups will be carried out only when there is a sound theoretical reason and enough data to support it.

Possible comparisons within the vegan participant group include

- People living in rural vs. urban areas;
- Supplement users vs. non-users (for themselves or their children);
- Participants who changed their diet during pregnancy vs. those who did not;
- Individuals who received professional advice vs. those who used informal sources like social media.

Possible comparisons within the group of medical experts include

- Those who support vs. those who reject vegan diets during pregnancy and childhood;
- Those who advise supplementation vs. those who discourage it;
- Differences between professions (e.g., pediatricians, pharmacists, dietitians).

Depending on the final data, additional group comparisons will be added. We will only report comparisons that are well justified and provide useful insights.

### 2.8. Trial Status

Prior to launching the main survey, a pretest was conducted with 20 participants from the vegan community and 10 participants from the group of medical experts to refine and validate the questionnaire. Following pretesting and ethical approval from the Ethics Committee of the University of Vienna (Reference Number: 01021; see Supplementary Materials for the ethics decision), the survey targeting vegans was launched in October 2023, while the survey for medical experts commenced in February 2024. The study was registered on clinicaltrials.gov (NCT06669819) in October 2024 (31 October 2024). The survey will remain accessible for participant responses until end of July 2025.

### 2.9. Data Security

Survey data is collected using the online survey tool LimeSurvey. Participants who want to take part in the scientific survey follow a link/URL that takes them to the survey. All anonymous data will be transferred from LimeSurvey to EXCEL (Microsoft Corp., Redmond, WA, USA) and further to R version 4.5.1 (R Core Team) and IBM SPSS Statistics version 31 (IBM Corp., Armonk, NY, USA) for processing and statistical evaluation. Data that are in the stage of statistical analysis and evaluation will be stored at the u:cloud storage system of the University of Vienna (protected server). Access to the LimeSurvey Cloud instance and u:cloud storage system will only be possible by password personalized for the principal investigator (PI) and study directors. No names of participants will be asked or recorded. Only answered, anonymized questionnaires will be stored until final analysis and publication of data at the LimeSurvey cloud instance and the u:cloud storage system.

Participation in the survey is voluntary and can be canceled at any time. The survey tool LimeSurvey processes survey data completely anonymously. LimeSurvey is an international survey tool, but the protected server where survey data is hosted (saved at the LimeSurvey Cloud instance) is located in Germany. Data about names, Email or IP-addresses are not saved. Collected data is protected by the consent of the General Data Protection Regulation (GDPR). LimeSurvey guarantees full EU GDPR compliance because of the selection of the German server as server location. Survey/response data is stored in a separate database with a separate username/password for each LimeSurvey Cloud instance. The connection of the used browser to LimeSurvey servers is also encrypted using SSL (Secure Sockets Layer) which is known to be the standard technology for securing Internet connections and protecting sensitive data. The connection is encrypted, which ensures protection, security and anonymity for participants and their personal data. The data received will be treated confidentially and processed and evaluated exclusively for scientific purposes. The link/URL for the survey will remain accessible for approximately 2 years (July 2023–July 2025).

## 3. Results/Outcome Measures

### 3.1. Primary Outcome Measures

Primary outcome measures include influential factors that affect how vegans approach their diet during pregnancy. Furthermore, details about decisions regarding the use of DS of vegan pregnant women and parents’ administration of DS to their children, which impacts their nutritional status and health, are essential outcomes.

Other primary outcome measures involve methods of information gathering related to a vegan diet and DS among vegans, as well as opinions and recommendations from medical experts on these topics and their advice to support or reject a vegan diet and DS. All findings will be based on the frequency and prevalence of collected responses.

### 3.2. Secondary Outcome Measures

Secondary outcome measures include gathering information on socio-economic factors such as age, gender, highest educational attainment, place of origin (urban/rural), household size, and pregnancy status. This data is collected to link these variables with responses on vegan diets and dietary supplement usage, to identify and cluster behavioral patterns among vegans. Additionally, medical experts such as physicians, pharmacists, and dietitians are asked about their practice location (postal code) and, in the case of physicians, their specialty (gynecology, pediatrics, or general medicine) to understand how these factors might influence their perspectives, similar to the approach taken with vegan participants.

Secondary outcome measures for vegans are as follows: assessment of health perceptions during pregnancy and childhood, evaluation of perceived health benefits associated with a purely plant-based diet, and the interpretation of their own vegan lifestyle: This includes factors such as frequency of dietary exceptions, perceived health benefits of veganism, duration of adherence to a vegan diet, whether vegans maintained a vegan diet during one or more pregnancies, and any potential influences or reasons that may have hindered or altered their dietary choices. Other aspects include understanding of DS (such as perceived effectiveness, categorization, selection criteria), self-reported compliance, consistency of supplement administration by parents to vegan-fed children, support or opposition to supplement use during pregnancy and childhood, data on dosage estimations, challenges with administration based on form (e.g., syrup, capsules, tablets), evaluation of nutritional needs for vegan pregnancy and childhood, willingness to receive guidance on vegan nutrition during pregnancy and early childhood, and subjective trust in advisory sources regarding a vegan diet during these stages.

Secondary outcome measures for medical experts include gathering the personal opinions of physicians, pharmacists, and dietitians regarding DS and a vegan diet during pregnancy and childhood. This encompasses assessing the perceived health impacts of a vegan diet in these life stages. Furthermore, the study evaluates compliance with supplement intake among vegans, the resulting implications, and any challenges in administering DS to their children. Experts’ experiences with advising vegan diets and their stance on recommending or discouraging the use of DS should be assessed as well.

A statistical analysis on the survey data will be conducted to identify and interpret any previously unrecognized relationships between responses and potential consequences for vegans.

## 4. Discussion

As this is a study protocol, findings are not yet available. However, this section highlights key knowledge gaps and discusses the rationale behind the VedieS study. Further it illustrates how the study may help address unresolved questions in this field.

Whether and to what extent external factors influence vegans and their diet during pregnancy, infancy and childhood has not yet been comprehensively investigated. There is still limited data on the opinions and knowledge levels of medical experts regarding a vegan diet and the use of dietary supplements. A position paper by Klug et al. [22] emphasizes that many medical experts lack practical training on how to support a vegan lifestyle during pregnancy and childhood. Similarly, Jeitler et al. [56] reported that even medical experts who took part in a survey at a conference on plant-based diets were unsure how to apply their knowledge in practice and noted challenges in monitoring important nutrients, especially vitamin B12, in their patients. Since many vegans feel they cannot fully rely on comprehensive guidance from physicians, pharmacists, or dietitians, they often seek information from non-professional sources. This, in turn, may result in a limited and potentially inaccurate understanding of nutritional requirements, which can increase the risk of health impairments caused by misleading or incorrect advice. Studies have already confirmed that vegan parents and pregnant women often turn to alternative information sources because they receive limited support from medical experts, feel judged, or fear being misunderstood: Farella et al. [57] describe how mistrust and communication barriers can strain the relationship between pediatricians and vegan families. Pereboom et al. [58] found that vegan mothers often experienced limited support or perceived skepticism from medical experts, which led them to seek guidance from alternative platforms regarding the diet of their vegan-fed children. It remains unclear which specific information sources, apart from medical experts, have the greatest influence, how they shape aspects of a vegan diet such as its continuation, modifications, DS use, or dosage issues, and whether this influence could involve potential health risks. Hence, the emphasis of the VedieS study is also health risk analysis and will result in prevention-approaches for vegans. Furthermore, there is hardly any scientific data on the influential factors mentioned, information gathering, reasons for choosing certain DS for pregnant vegans and their children, difficulties in administering DS to children, compliance with DS and possible resulting health impairments. A recent systematic review and meta-analysis by Papadopoulou et al. [45] found that following a strict vegetarian diet during pregnancy was linked to a higher risk of small-for-gestational-age infants and a lower average birth weight compared to omnivorous diets. This indicates possible knowledge gaps among pregnant vegans regarding a balanced diet and DS use, as well as reliance on potentially inadequate sources of information, underscoring the need for reliable and accessible advice. The VedieS study can contribute new insights into these areas, which have received little research attention so far and can affect a healthy, low-risk pregnancy and normal child development. Improving food and nutritional health literacy and developing evidence-based supplementation guidelines are essential to support informed decisions and reduce health risks in this vulnerable group [22,56]. A qualitative study by Cnossen et al. [59] found that pregnant vegans often receive misleading or insufficient advice from medical experts, indicating a degree of stigmatization and a lack of appropriate nutritional counseling. A recent scoping review from McLeod et al. [60] also shows that medical experts’ knowledge of a plant-based diet during pregnancy is limited and strongly linked to a lack of specialized nutrition training. It is important to reveal deficits in the provision and quality of information for vegans and to identify needs for competent information delivery. In this way, the advice given to vegans can be improved. By including the gender aspect and socio-demographic characteristics further differences will be taken into consideration.

Research gaps concerning the administration of DS, the consistency of taking them and compliance shall be closed by statistically evaluating data from performed surveys (vegans, pediatricians, gynecologists, general practitioners, pharmacists, and dieticians). In addition, causes for the intake or rejection of DS (from a social and socio-demographic perspective) are to be examined. Resulting risks concerning the supply of nutrients are to be determined as well as the role that information sources play in this context. It is precisely these unanswered questions that underline the relevance of the VedieS study, as they are directly related to risk prevention. According to a recent study by Meulenbroeks et al. [61], pregnant women following a vegan diet often receive limited or no specific dietary guidance from obstetric healthcare providers. Only 19% of participants reported having received nutritional counseling that was specific to their needs during pregnancy. The VedieS study’s approach to identifying the cause of nutrient deficiencies during vegan pregnancy and childhood, due to a fundamental lack of information, is new and can be important for preventing health risks for vegans. Unsupplemented vegan pregnancies are associated with a higher risk of adverse outcomes [45]. These findings highlight the importance of guideline-based supplementation for vegans during sensitive life stages. The quality of the information sources used by vegans is most likely dependent on the existing attitude of the information providers. If medical experts have a negative attitude towards a vegan diet and DS, their advice may be inadequate—either due to a lack of knowledge about a purely plant-based diet, or because a vegan diet is discouraged per se without providing additional information. Knowledge gaps regarding vegan diets remain common among medical experts, likely due to limited nutrition education and a lack of familiarity with plant-based dietary patterns [44,60]. As a result of limited professional support, many vegan pregnant women turn to alternative sources of information such as books, online platforms, or peer communities to address their specific dietary needs [61]. It is important to know exactly where the information received comes from in order to be able to draw conclusions about its safety and reliability. In a cross-sectional online survey by Fuschlberger et al. [62] among Austrian vegans, 92% reported taking vitamin B_12_ through DS or fortified foods, yet fewer than 10% had consulted a medical expert. However, more research is needed to find out exactly which specific sources of information are being used.

The results obtained from the VedieS study can show possible gaps in the information provided to pregnant vegans and parents of vegan-fed children and promote the provision of information to prevent health risks. In addition, there is still little data on the opinions of physicians, pharmacists and dieticians regarding this topic. The information provided by medical experts could be one of the most important aspects when it comes to selecting information sources for vegans. If vegans avoid nutritional advice from medical experts due to a negative attitude, this represents a potential health risk factor [60].

Other, previous studies on vegan diets during pregnancy and early childhood have mostly focused on clinical or biochemical outcomes (e.g., nutrient levels) and have not paid comprehensive attention to social influences, quality of advice, or information sources [25,45,63]. Qualitative research has shown that pregnant vegans frequently encounter challenges in accessing trustworthy information [59]. They also report feeling judged and often receive advice that is insufficient or not based on scientific evidence. These results indicate that social and information-related factors have so far been insufficiently considered in research, an area the VedieS study aims to address. A systematic review by Meulenbroeks et al. [24] confirmed that vegan diets during pregnancy are possible, but only with careful supplementation and professional support. Nevertheless, studies by McLeod et al. [60] and Barbier et al. [46] found that many medical experts feel unprepared to advise vegan families or hold critical views on a vegan diet, which may result in conflicting recommendations and reduced patient trust. Additionally, data from Leiva et al. [10] highlights that, even when general clinical outcomes appear normal, many vegan pregnant women still have low levels of critical nutrients like vitamin B12 or DHA. This supports our intent to explore how information sources and risk perception influence a vegan diet and DS intake during pregnancy and early childhood. These studies reveal an obvious need to understand how trust in medical experts, use of alternative information sources, and risk awareness shape the dietary practices and supplementation decisions of vegan families during pregnancy and early childhood. The results of the VedieS study could help to improve medical experts’ food and nutritional health literacy regarding purely plant-based diets and support the health of pregnant vegans and vegan-fed children by providing guidance that is better adapted to their needs.

## 5. Conclusions

The VedieS study is the first to incorporate perspectives not only from vegan pregnant women and parents but also from medical experts on the topic of a vegan diet and the intake of DS during pregnancy and childhood. It offers new, valuable insights that can positively impact health risk prevention through preparedness for enhanced information provision. With the growing number of vegans and the increasing relevance of vegan diets during pregnancy and childhood, the study delivers essential insights that improve and expand the availability of information for these vulnerable groups. This practical approach, which aims to enrich and refine information resources for vegans, underscores the VedieS study’s scientific contributions and its broader utility and necessity. Enhanced information availability for vegans supports health during pregnancy and benefits childhood health. By highlighting the importance of comprehensive guidance, the study encourages physicians, pharmacists, and dietitians to offer meaningful support, reducing reliance on potentially dubious sources of information. This approach can optimize the food and nutritional health literacy of vegans, and the knowledge required for medical experts to provide professional guidance on a vegan diet and DS.

### 5.1. Practical Implications

Given the growing prevalence of vegan diets, targeted research on vulnerable populations—particularly vegan pregnant women and children—is increasingly important. Although there is a lot of information on critical nutrients of a vegan diet and guidelines on nutrient requirements during pregnancy and childhood, many studies have not yet taken into account whether and how vegans even receive the necessary information to ensure a well-balanced, purely plant-based diet. Since an intensive development phase (pregnancy, early childhood up to the age of 5 years) together with a diet that completely excludes animal products represents a particularly risky combination for health, the study is timely and important.

A balanced vegan diet requires food and nutritional health literacy, which can become very complex, especially during pregnancy and childhood. It must be ensured that medical experts can offer comprehensive advice. Due to the increasing number of existing vegans, the group of vegan pregnant women and children should not be marginalized in medical circles and competent information should be available. The study thus addresses an increasingly important topic that is making its way into the field of public health.

### 5.2. Limitations and Strengths of the Study

Since the study is based on survey responses, there is a possibility of over- or under-reporting due to the subjective self-assessment of participants regarding, e.g., compliance when taking DS, parental administration consistency and correct dosage of DS, the frequency of exceptions to a vegan diet, and the period within parents and children already follow a vegan diet. Data is based on participants’ motivation to self-report. Hence, only responses from actual participants are considered which does not necessarily capture the opinion of all vegans and medical experts. Although all genders are included, it is likely that women will be overrepresented, because of the focus on pregnant/breastfeeding women and secondarily vegan parents. Nonetheless, the strengths of this study outweigh its limitations. This is the first time data has been collected considering perspectives of both vegans and different medical specialists regarding a vegan diet and DS during pregnancy and childhood. Resulting insights could help to shift experts’ attitudes from rejection of a fully plant-based lifestyle and the refusal of information provision on a vegan diet and DS during these life stages to more comprehensive, informed guidance for vegans. Experts’ acceptance of the need and the willingness for detailed advice can be promoted which could cause distancing from devaluing a vegan lifestyle. The value of gathering these fresh data on public health nutrition ultimately outweighs limitations as results provide significant benefits to both vegans and medical experts alike.

## Data Availability

Collected survey data will be available in future publications after statistical evaluations have been completed. Currently only the PI as well as the study directors has data access. Inquiries regarding access to the datasets can be addressed to a0225229@unet.univie.ac.at.

## Supplementary Materials

The following supporting information can be downloaded at: https://www.mdpi.com/article/10.3390/ijerph22081210/s1.

## Abbreviations

The following abbreviations are used in this manuscript:

DS	Dietary Supplements
PI	Principal Investigator
WHS	Wolfgang Huber-Schneider
KHW	Karl-Heinz Wagner
IK	Ingrid Kiefer
HBM	Health Belief Model
TPB	Theory of Planned Behavior
DHA	Docosahexaenoic acid
EPA	Eicosapentaenoic acid
