# Supplementary material for: Can Dietary Supplements Be Linked to a Vegan Diet and Health Risk Modulation During Vegan Pregnancy, Infancy, and Early Childhood? The VedieS Study Protocol for an Explorative, Quantitative, Cross-Sectional Study
Source: Int J Environ Res Public Health. 2025 Jul 31;22(8):1210. doi: 10.3390/ijerph22081210 (PMC12386434; doi:10.3390/ijerph22081210)
Supplement: Supplementary file 1 [file ijerph-22-01210-s001.zip › S1_3677589.pdf]

Befragung von Veganer:innen zu veganer Ernährung sowie zur Einnahme von Nahrungsergänzungsmitteln während der Schwangerschaft und im Säuglings- und frühen Kindesalter

Teilnehmer:inneninformation und Einwilligungserklärung zur Teilnahme an der Studie:

Arbeitstitel der Studie:

Können Nahrungsergänzungsmittel mit veganer Ernährung und einer Modulation des Gesundheitsrisikos während Schwangerschaft, Säuglings- und frühen Kindesalters in Verbindung gebracht werden?

Sehr geehrte Teilnehmerin, sehr geehrter Teilnehmer,

wir laden Sie ein, an der oben genannten Studie teilzunehmen.

Ihre Teilnahme an dieser Studie erfolgt freiwillig. Sie können jederzeit, ohne Angabe von Gründen, Ihre Teilnahme im Verlauf der Befragung beenden und somit Ihre Teilnahmebereitschaft an der Studie, durch Abbruch der Befragung, widerrufen. Die Ablehnung der Teilnahme oder ein vorzeitiges Ausscheiden aus dieser Studie hat keine nachteiligen Folgen für Sie. Diese Art von Studien ist notwendig, um verlässliche neue wissenschaftliche Forschungsergebnisse zu gewinnen. Unverzichtbare Voraussetzung für die Durchführung von Studien ist jedoch, dass Sie Ihr Einverständnis zur Teilnahme an dieser Studie erklären. Es werden KEINE sensiblen Daten wie Name, E-Mail-Adresse oder IP-Adresse erhoben. Die Teilnahme an der Befragung wird **ca. 25 Minuten** Ihrer Zeit in

Anspruch nehmen. Bitte lesen Sie den folgenden Text sorgfältig durch und zögern Sie nicht, Fragen zu stellen. Bei Rückfragen wenden Sie sich bitte an den angegebenen Kontakt des **studiendurchführenden Doktoratsstudenten Wolfgang Huber-Schneider** (Kontakt ganz unten im Text).

Bitte bestätigen Sie die Einwilligungserklärung nur (Bestätigung = Anklicken von "Weiter" - der Beginn der Befragung gilt als Bestätigung der Einwilligungserklärung):

- wenn Sie Art und Ablauf der Studie vollständig verstanden haben,
- wenn Sie bereit sind, der Teilnahme zuzustimmen und
- wenn Sie sich über Ihre Rechte als Teilnehmer:in an dieser Studie im Klaren sind.

- **Was ist der Zweck der Studie?**

Das Ziel der Studie ist es, Zusammenhänge von Beratung durch medizinische Expert:innen (z.B. durch Arzt/Ärztin, Apotheker:innen) und anderen Einflussfaktoren (z.B. soziales Umfeld, soziale Medien), mit der veganen Ernährung und der Einnahme von Nahrungsergänzungsmitteln in Schwangerschaft, Säuglingsalter und früher Kindheit (bis zu einem Alter von 5 Jahren) von Veganer:innen zu klären. Somit können mögliche gesundheitliche Risiken für betreffende Veganer:innen in Erfahrung gebracht und präventiv minimiert werden. Bitte helfen Sie mit Ihrer Teilnahme an der Befragung mit, Informationsmöglichkeiten und Risikoprävention für die Gesundheit von Veganer:innen zu optimieren. Die Befragung wird in Zusammenhang mit einem PhD Projekt durchgeführt.

- **Wie läuft die Studie ab?**

Der Fragebogen richtet sich an Veganer:innen. Fragen zur veganen Ernährung und Einnahme von Nahrungsergänzungsmitteln in Schwangerschaft, Säuglingsalter und früher Kindheit (bis zu einem Alter von 5 Jahren) werden gestellt. Die Beantwortung des Fragenbogens wird ca. **25 Minuten in Anspruch nehmen.**

- **Worin liegt der Nutzen einer Teilnahme an der Studie?**

Es ist nicht zu erwarten, dass Sie aus Ihrer Teilnahme einen direkten, persönlichen Nutzen ziehen werden, allerdings leisten Sie durch die Beantwortung des Fragebogens einen wichtigen Beitrag, mögliche gesundheitliche Risiken für schwangere Veganerinnen und vegan ernährte Kinder präventiv zu minimieren. Aus wissenschaftlicher Sicht soll, durch Erforschung von Informationsquellen und deren Einflussnahme auf die Ernährung und Einnahme von Nahrungsergänzungsmitteln, von und für genannte Veganer:innen, die Informationsversorgung und Informationsqualität für Veganer:innen optimiert werden.

- **Gibt es Risiken bei der Durchführung der Studie und ist mit Beschwerden oder anderen**

Begleiterscheinungen zu rechnen?

Es ist nicht zu erwarten, dass die Teilnahme an der Studie mit Unannehmlichkeiten oder Risiken verbunden ist.

Einschlusskriterien für die Teilnahme an der Studie:

1. Schwangere und Teilnehmerinnen (ab 18 Jahren), die sich während ihrer Schwangerschaft/en vegan ernähren/ernährt haben

2. Eltern, die ihr/e Kind/er (bis zu einem Alter von 5 Jahren) vegan ernähren/ernährt haben

3. Mütter, die sich vegan ernähren und ihre Kinder stillen

4. Bestätigung der Teilnehmer:inneninformation und Einverständniserklärung

Ausschlusskriterien für die Teilnahme an der Studie:

5. Teilnehmer:innen (ab 18 Jahren), die sich während ihrer Schwangerschaft/en nicht vegan ernähren/ernährt haben

6. Eltern, die ihr/e Kind/er (bis zu einem Alter von 5 Jahren) nicht vegan ernähren/ernährt haben

7. Keine Bestätigung der Teilnehmer:inneninformation und Einverständniserklärung

Für die Teilnahme an der Studie muss zumindest ein Einschlusskriterium 1,2 oder 3 (IMMER inkl. 4) erfüllt sein. Der Ausschluss aus der Studie erfolgt, wenn 7 erfüllt ist. Wenn nur 5 ODER 6 erfüllt sind, und 7 nicht, ist die Teilnahme möglich.

- **Hat die Teilnahme an der Studie sonstige Auswirkungen auf die Lebensführung und welche Verpflichtungen ergeben sich daraus?**

Nein, Sie können jederzeit an der Befragung teilnehmen und diese zu jedem beliebigen Zeitpunkt, ohne Konsequenzen oder sonstigen Auswirkungen, abbrechen.

- **Was ist zu tun beim Auftreten von Beschwerdesymptomen, unerwünschten Begleiterscheinungen und/oder Verletzungen?**

Da es sich um eine Befragung handelt, ist mit keinen Beschwerdesymptomen zu rechnen.

- **Wann wird die Studie vorzeitig beendet?**

Sie können jederzeit, auch ohne Angabe von Gründen, Ihre Teilnahmebereitschaft widerrufen und aus der Studie ausscheiden (indem Sie die Befragung abbrechen), ohne dass dadurch irgendwelche Nachteile für Sie entstehen.

- **In welcher Weise werden die im Rahmen dieser Studie gesammelten Daten verwendet?**

Innerhalb Ihrer Teilnahme an der Befragung ist es nicht erforderlich, persönliche Daten anzugeben. Die Beantwortung der Fragen erfolgt völlig anonym. Anonyme, gesammelte Daten werden ausschließlich für statistische Zwecke gespeichert. Es werden ausnahmslos keine persönlichen Daten (z.B. Name, IP-Adresse, E-Mail-Adresse) erfragt oder gespeichert. Keine Rückschlüsse auf Ihre Person sind möglich.

- **Entstehen für die Teilnehmer:innen Kosten? Gibt es einen Kostenersatz oder eine Vergütung?**

Durch die Teilnahme an dieser Studie entstehen für Sie keinerlei Kosten. Es gibt keinen Kostenersatz und keine Vergütung für Teilnehmer:innen der Befragung/Studie.

- **Möglichkeit zur Diskussion weiterer Fragen**

Für weitere Fragen im Zusammenhang mit dieser Studie steht Ihnen Wolfgang Huber-Schneider gern zur Verfügung. Auch Fragen, die Ihre Rechte als Teilnehmer:in betreffen, werden Ihnen selbstverständlich beantwortet. Sobald allgemeine Ergebnisse dieser Studie vorliegen, können Sie ebenfalls darüber informiert werden, falls Sie dies wünschen.

- **Kontakt - Studienteam/Doktoratsstudent**

Die Befragung wird im Zuge der Dissertation von **Wolfgang Huber-Schneider** am **Department für Ernährungswissenschaften der Universität Wien in Kooperation mit der AGES (Österreichische Agentur für Gesundheit und Ernährungssicherheit)** durchgeführt. Bei Fragen kontaktieren Sie:

Doktoratsstudent: Mag. pharm. Wolfgang Huber-Schneider, a00225229@unet.univie.ac.at

Studienleitung: Univ.-Prof. Mag. Dr. Karl-Heinz Wagner (Department für Ernährungswissenschaften Universität Wien), Univ.-Doz.in Mag.a Dr.in Ingrid Kiefer (AGES), Mag. pharm. Wolfgang Huber-Schneider (Doktoratsstudent)
Prüfarzt: Univ.-Prof. Dr. Daniel König (Institut für Sportwissenschaft Universität Wien)

Sobald Sie auf **"Weiter"** klicken, stimmen Sie automatisch Ihrer Teilnahme zur Befragung zu.

In dieser Umfrage sind 75 Fragen enthalten.

Allgemeines

Geschlecht *

Bitte wählen Sie eine der folgenden Antworten:
Bitte wählen Sie nur eine der folgenden Antworten aus:

- ◯ Weiblich
- ◯ Männlich
- ◯ Divers

Alter (in Jahren) *

Bitte wählen Sie eine der folgenden Antworten:
Bitte wählen Sie nur eine der folgenden Antworten aus:

- ◯ Unter 18
- ◯ 18-20
- ◯ 21-30
- ◯ 31-40
- ◯ 41-50
- ◯ 51-60
- ◯ 61 oder älter

Höchste abgeschlossene Ausbildung *

Bitte wählen Sie eine der folgenden Antworten:
Bitte wählen Sie nur eine der folgenden Antworten aus:

- ◯ Pflichtschule
- ◯ Lehre/Berufsausbildung/Berufliche Grundbildung
- ◯ Matura/Abitur
- ◯ Kolleg
- ◯ Fachhochschule
- ◯ Universität
- ◯ Sonstiges

Haushaltsnettoeinkommen monatlich (Einkommen aller Haushaltsmitglieder - netto - inkl. Förderungen und/oder Beihilfen) - in EUR bzw. CHF *

Bitte wählen Sie eine der folgenden Antworten:
Bitte wählen Sie nur eine der folgenden Antworten aus:

- ◯ Bis 1.500
- ◯ 1.501 - 2.000
- ◯ 2.001 - 2.500
- ◯ 2.501 - 3.000
- ◯ 3.001 - 3.500
- ◯ 3.501 - 4.000
- ◯ Über 4.001
- ◯ Keine Angabe

Haushaltsgröße (Personen inkl. Kinder) *

Bitte wählen Sie eine der folgenden Antworten:
Bitte wählen Sie nur eine der folgenden Antworten aus:

- ◯ 1
- ◯ 2
- ◯ 3
- ◯ 4 oder mehr

In welchem Land leben Sie? *

Bitte wählen Sie eine der folgenden Antworten:
Bitte wählen Sie nur eine der folgenden Antworten aus:

- ◯ Österreich
- ◯ Deutschland
- ◯ Schweiz
- ◯ Italien
- ◯ Anderes EU-Land
- ◯ Sonstiges

Wo wohnen Sie (Postleitzahl)? *

Bitte geben Sie Ihre Antwort hier ein:

Schwangerschaft *

Beantworten Sie diese Frage nur, wenn folgende Bedingungen erfüllt sind:
Antwort war 'Divers' *oder* 'Weiblich' bei Frage ' [G1Q00001]' (Geschlecht)

Bitte wählen Sie eine der folgenden Antworten:
Bitte wählen Sie nur eine der folgenden Antworten aus:

- ◯ Ich bin aktuell schwanger
- ◯ Ich habe bereits eine Schwangerschaft durchlebt
- ◯ Ich habe bereits mehrere Schwangerschaften durchlebt
- ◯ Ich bin aktuell schwanger und habe schon ein oder mehrere Schwangerschaften durchlebt
- ◯ Ich bin NICHT schwanger und habe auch noch KEINE Schwangerschaft durchlebt

Haben Sie Kinder? *

Bitte wählen Sie eine der folgenden Antworten:
Bitte wählen Sie nur eine der folgenden Antworten aus:

- ◯ Ja
- ◯ Nein

Wie viele Kinder haben Sie? *

Beantworten Sie diese Frage nur, wenn folgende Bedingungen erfüllt sind:
Antwort war 'Ja' bei Frage ' [G1Q00009]' (Haben Sie Kinder?)

Bitte wählen Sie eine der folgenden Antworten:
Bitte wählen Sie nur eine der folgenden Antworten aus:

- ◯ 1
- ◯ 2
- ◯ 3
- ◯ Mehr als 3

Stillen/Stillten Sie Ihre Kinder? *

Beantworten Sie diese Frage nur, wenn folgende Bedingungen erfüllt sind:
Antwort war 'Weiblich' *oder* 'Divers' bei Frage ' [G1Q00001]' (Geschlecht) *und* Antwort war 'Ich habe bereits mehrere Schwangerschaften durchlebt' *oder* 'Ich bin aktuell schwanger und habe schon ein oder mehrere Schwangerschaften durchlebt' bei Frage ' [G1Q00008]' (Schwangerschaft) *und* Antwort war 'Ja' bei Frage ' [G1Q00009]' (Haben Sie Kinder?) *und* Antwort war '2' *oder* '3' bei Frage ' [G1Q00011]' (Wie viele Kinder haben Sie?)

Bitte wählen Sie eine der folgenden Antworten:
Bitte wählen Sie nur eine der folgenden Antworten aus:

- ◯ Ja
- ◯ Nein
- ◯ Ich habe nicht alle meine Kinder gestillt

Stillen/Stillten Sie ihr Kind? *

Beantworten Sie diese Frage nur, wenn folgende Bedingungen erfüllt sind:
Antwort war 'Weiblich' *oder* 'Divers' bei Frage ' [G1Q00001]' (Geschlecht) *und* Antwort war 'Ich habe bereits eine Schwangerschaft durchlebt' bei Frage ' [G1Q00008]' (Schwangerschaft) *und* Antwort war 'Ja' bei Frage ' [G1Q00009]' (Haben Sie Kinder?) *und* Antwort war '1' bei Frage ' [G1Q00011]' (Wie viele Kinder haben Sie?)

Bitte wählen Sie eine der folgenden Antworten:
Bitte wählen Sie nur eine der folgenden Antworten aus:

- ◯ Ja
- ◯ Nein

Gesundheit

Was bedeutet Gesundheit für Sie? (Reihen Sie bitte maximal 3 Antworten) *

Alle Ihre Antworten müssen unterschiedlich sein, und müssen zugeordnet sein.
Bitte wählen Sie maximal 3 Antworten.
Bitte nummerieren Sie jede Box in der Reihenfolge Ihrer Präferenz, beginnen mit 1 bis 8
Bitte wähle Sie nicht mehr als 3 Einträge aus.

☐ Ein Zustand völligen psychischen, physischen und sozialen Wohlbefindens

☐ Das Freisein von Krankheit und Gebrechen

☐ Sich wohlzufühlen - auch ohne Diagnosen und Untersuchungsergebnisse

☐ An keiner diagnostizierten Krankheit zu leiden

☐ Den Alltag ohne Einschränkungen leben zu können

☐ Psychisch und physisch belastbar zu sein

☐ Glücklich und beschwerdefrei zu sein

☐ Überdurchschnittlich leistungsfähig zu sein

Gesundheit 2

Was bedeutet Gesundheit in der Schwangerschaft vor allem für Sie? *

Bitte wählen Sie eine der folgenden Antworten:
Bitte wählen Sie nur eine der folgenden Antworten aus:

- ◯ Eine komplikationsfreie Schwangerschaft
- ◯ Eine Schwangerschaft, in der sich das Kind durch Bereitstellung aller benötigten Nährstoffe ideal entwickelt
- ◯ Eine Schwangerschaft, in der das Kind den Entwicklungsfortschritten (lt. ärztlichen Untersuchungen) bestmöglich entspricht
- ◯ Eine Schwangerschaft, in der man sich wohl fühlt - auch ohne ärztliche Bestätigung des Gesundheitszustandes
- ◯ Eine Schwangerschaft, die zeitlich wie erwartet und planmäßig verläuft

Gesundheit 3

Was bedeutet Gesundheit im Kindesalter vor allem für Sie? *

Bitte wählen Sie eine der folgenden Antworten:
Bitte wählen Sie nur eine der folgenden Antworten aus:

- ◯ Das Kind macht physische und psychische Fortschritte, die seinem Alter entsprechen (lt. Arzt/Ärztin, Pädagog:innen etc.)
- ◯ Das Kind macht physische und psychische Fortschritte, die ich als angemessen empfinde
- ◯ Das Kind neigt nicht zu Infekten und hat ein starkes Immunsystem
- ◯ Das Kind ist meiner Beobachtung nach glücklich und ausgeglichen
- ◯ Das Kind macht überdurchschnittliche physische und psychische Fortschritte

VEGANE ERNÄHRUNG: DEFINITION UND BEWEGGRÜNDE

Wie ernähren Sie sich? (Mehrfachauswahl möglich) *

Wählen Sie alle zutreffenden Optionen
Bitte wählen Sie alle zutreffenden Antworten aus:

- ☐ Ich ernähre mich IMMER rein pflanzlich (vegan)
- ☐ Ich ernähre mich FAST IMMER vegan, aber selten nehme ich auch Honig und/oder andere Imkereierzeugnisse (z.B. Propolis, Gelee Royal) zu mir
- ☐ Ich ernähre mich FAST IMMER vegan, aber selten nehme ich auch Molkereierzeugnisse (z.B. Milch, Butter, Joghurt) zu mir
- ☐ Ich ernähre mich FAST IMMER vegan, aber selten esse ich auch Fisch und/oder Meeresfrüchte (z.B. Garnelen, Muscheln)
- ☐ Ich ernähre mich FAST IMMER vegan, aber selten esse ich auch Fleisch (z.B. Huhn)
- ☐ Ich ernähre mich FAST IMMER vegan, aber selten esse ich auch Fisch und Fleisch

Wie oft kommt es vor, dass Sie eine Ausnahme von der veganen Ernährung machen? *

Beantworten Sie diese Frage nur, wenn folgende Bedingungen erfüllt sind:
Antwort war 'Ich ernähre mich FAST IMMER vegan, aber selten esse ich auch Fisch und Fleisch' *oder* 'Ich ernähre mich FAST IMMER vegan, aber selten esse ich auch Fleisch (z.B. Huhn)' *oder* 'Ich ernähre mich FAST IMMER vegan, aber selten esse ich auch Fisch und/oder Meeresfrüchte (z.B. Garnelen, Muscheln)' *oder* 'Ich ernähre mich FAST IMMER vegan, aber selten nehme ich auch Molkereierzeugnisse (z.B. Milch, Butter, Joghurt) zu mir' *oder* 'Ich ernähre mich FAST IMMER vegan, aber selten nehme ich auch Honig und/oder andere Imkereierzeugnisse (z.B. Propolis, Gelee Royal) zu mir' bei Frage '[G5Q00001]' (Wie ernähren Sie sich? (Mehrfachauswahl möglich))

Bitte wählen Sie eine der folgenden Antworten:
Bitte wählen Sie nur eine der folgenden Antworten aus:

- ◯ Ca. ein- bis zweimal im Jahr
- ◯ Ca. ein- bis zweimal im halben Jahr
- ◯ Ca. ein- bis zweimal im Monat
- ◯ Ca. ein- bis zweimal in der Woche

Ist vegane Ernährung gesund? *

Bitte wählen Sie eine der folgenden Antworten:
Bitte wählen Sie nur eine der folgenden Antworten aus:

- ◯ Ja
- ◯ Nein
- ◯ Weiß nicht

Warum ernähren Sie sich vegan? (Mehrfachauswahl möglich) *

Wählen Sie alle zutreffenden Optionen
Bitte wählen Sie alle zutreffenden Antworten aus:

- ☐ Aus gesundheitlichen Gründen
- ☐ Aus Gründen des Tierschutzes
- ☐ Aus Gründen des Klimaschutzes
- ☐ Weil mir tierische Lebensmittel nicht schmecken
- ☐ Sonstiges

Wann haben Sie begonnen, sich vegan zu ernähren? *

Beantworten Sie diese Frage nur, wenn folgende Bedingungen erfüllt sind:
Antwort war 'Weiblich' *oder* 'Divers' bei Frage ' [G1Q00001]' (Geschlecht) *und* Antwort war 'Ich bin aktuell schwanger' *oder* 'Ich habe bereits eine Schwangerschaft durchlebt' *oder* 'Ich habe bereits mehrere Schwangerschaften durchlebt' *oder* 'Ich bin aktuell schwanger und habe schon ein oder mehrere Schwangerschaften durchlebt' bei Frage ' [G1Q00008]' (Schwangerschaft)

Bitte wählen Sie eine der folgenden Antworten:
Bitte wählen Sie nur eine der folgenden Antworten aus:

- ◯ Vor meiner/n Schwangerschaft/en
- ◯ Während meiner Schwangerschaft/en
- ◯ Zwischen zwei meiner Schwangerschaften (d.h. Sie haben sich nicht während jeder Schwangerschaft vegan ernährt)

Ab welchem Trimenon (Schwangerschaftsdrittel) haben Sie sich vegan ernährt? *

Beantworten Sie diese Frage nur, wenn folgende Bedingungen erfüllt sind:
Antwort war 'Weiblich' *oder* 'Divers' bei Frage ' [G1Q00001]' (Geschlecht) *und* Antwort war 'Während meiner Schwangerschaft/en' bei Frage ' [G5Q00005]' (Wann haben Sie begonnen, sich vegan zu ernähren?)

Bitte wählen Sie eine der folgenden Antworten:
Bitte wählen Sie nur eine der folgenden Antworten aus:

- ◯ Erstes Trimenon
- ◯ Zweites Trimenon
- ◯ Drittes Trimenon

Haben Sie sich während Ihrer (aktuellen/letzten) Schwangerschaft immer vegan ernährt? *

Beantworten Sie diese Frage nur, wenn folgende Bedingungen erfüllt sind:
Antwort war 'Weiblich' *oder* 'Divers' bei Frage ' [G1Q00001]' (Geschlecht) *und* Antwort war 'Ich bin aktuell schwanger' *oder* 'Ich habe bereits eine Schwangerschaft durchlebt' *oder* 'Ich habe bereits mehrere Schwangerschaften durchlebt' *oder* 'Ich bin aktuell schwanger und habe schon ein oder mehrere Schwangerschaften durchlebt' bei Frage ' [G1Q00008]' (Schwangerschaft)

Bitte wählen Sie eine der folgenden Antworten:
Bitte wählen Sie nur eine der folgenden Antworten aus:

- ◯ Ich habe mich weiterhin vegan ernährt
- ◯ Ich habe mich vegetarisch ernährt
- ◯ Ich habe auch Fisch gegessen
- ◯ Ich habe auch Fisch und Fleisch gegessen

Weshalb haben Sie Ihre vegane Ernährungsweise geändert? (Mehrfachauswahl möglich) *

Beantworten Sie diese Frage nur, wenn folgende Bedingungen erfüllt sind:
Antwort war 'Ich habe mich vegetarisch ernährt' *oder* 'Ich habe auch Fisch gegessen' *oder* 'Ich habe auch Fisch und Fleisch gegessen' bei Frage ' [G5Q00007]' (Haben Sie sich während Ihrer (aktuellen/letzten) Schwangerschaft immer vegan ernährt?)

Wählen Sie alle zutreffenden Optionen
Bitte wählen Sie alle zutreffenden Antworten aus:

- [ ] Aus Angst, meinem Kind mit veganer Ernährung zu schaden
- [ ] Weil mein/e Arzt/Ärztin mir dazu geraten hat
- [ ] Weil mein:e Apotheker:in mir dazu geraten hat
- [ ] Weil mein:e Diätolog:in mir dazu geraten hat
- [ ] Weil meine Hebamme mir dazu geraten hat
- [ ] Weil Freund:innen, Familie und/oder Arbeitskolleg:innen mir dazu geraten haben
- [ ] Weil mir andere Veganer:innen dazu geraten haben
- [ ] Aufgrund von Informationen aus dem Internet oder den sozialen Medien (Instagram, Facebook etc.)
- [ ] Aufgrund von Informationen aus Fachliteratur- und Zeitschriften
- [ ] Aufgrund von Informationen aus Zeitschriften, Zeitungen und/oder Lifestyle-Magazinen
- [ ] Sonstiges

Weshalb haben Sie Ihre vegane Ernährungsweise nicht geändert? (Mehrfachauswahl möglich) *

Beantworten Sie diese Frage nur, wenn folgende Bedingungen erfüllt sind:
Antwort war 'Ich habe mich weiterhin vegan ernährt' bei Frage ' [G5Q00007]' (Haben Sie sich während Ihrer (aktuellen/letzten) Schwangerschaft immer vegan ernährt?)

Wählen Sie alle zutreffenden Optionen
Bitte wählen Sie alle zutreffenden Antworten aus:

- ☐ Alle Nährstoffe für Mutter und Kind wurden abgedeckt
- ☐ Weil ich Nahrungsergänzungsmittel zu mir genommen habe, um möglichen Nährstoffmängeln vorzubeugen
- ☐ Weil mein/e Arzt/Ärztin mir dazu geraten hat
- ☐ Weil mein:e Apotheker:in mir dazu geraten hat
- ☐ Weil mein:e Diätolog:in mir dazu geraten hat
- ☐ Weil meine Hebamme mir dazu geraten hat
- ☐ Weil Freund:innen, Familie und/oder Arbeitskolleg:innen mir dazu geraten haben
- ☐ Weil mir andere Veganer:innen dazu geraten haben
- ☐ Aufgrund von Informationen aus dem Internet oder den sozialen Medien (Instagram, Facebook etc.)
- ☐ Aufgrund von Informationen aus Fachliteratur- und Zeitschriften
- ☐ Aufgrund von Informationen aus Zeitschriften, Zeitungen und/oder Lifestyle-Magazinen
- ☐ Ich habe nicht über eine Ernährungsumstellung nachgedacht
- ☐ Sonstiges

Wie lange ernähren Sie sich schon vegan? *

Bitte wählen Sie eine der folgenden Antworten:
Bitte wählen Sie nur eine der folgenden Antworten aus:

- ◯ Weniger als einen Monat
- ◯ Ca. 3 Monate
- ◯ Ca. 6 Monate
- ◯ Ca. 1 Jahr
- ◯ Ca. 1-3 Jahre
- ◯ Ca. 3-5 Jahre
- ◯ Ca. 5-10 Jahre
- ◯ Über 10 Jahre

NAHRUNGSERGÄNZUNGSMITTEL: DEFINITION UND VERSTÄNDNIS

Welche Nahrungsergänzungsmittel kennen Sie? Nennen Sie **bis zu drei Beispiele** *

Bitte geben Sie Ihre Antwort(en) hier ein:

Was sind Nahrungsergänzungsmittel? (Mehrfachauswahl möglich) *

Wählen Sie alle zutreffenden Optionen

Bitte wählen Sie alle zutreffenden Antworten aus:

- ☐ Lebensmittel die zur Ergänzung der normalen Ernährung des Menschen dienen
- ☐ Konzentrate zur Aufnahme in kleinen, abgemessenen Mengen zur Einnahme in dosierter Form
- ☐ Typische Bestandteile von Nahrungsergänzungsmitteln sind Vitamine, Mineralstoffe, essentielle Fettsäuren, Aminosäuren und Pflanzenbestandteile
- ☐ Nahrungsergänzungsmittel sind Medikamente
- ☐ Nahrungsergänzungsmittel sind Lebensmittel wie Bonbons, Limonaden, Tees etc.
- ☐ Weiß nicht

Wie wirken Nahrungsergänzungsmittel? *

Bitte wählen Sie eine der folgenden Antworten:

Bitte wählen Sie nur eine der folgenden Antworten aus:

- ◯ Wie rezeptpflichtige Medikamente
- ◯ Wie frei verkäufliche Medikamente
- ◯ Wie Lebensmittel
- ◯ Wie Placebos (Scheinmedikamente ohne Wirkstoffe)
- ◯ Sind in ihrer Wirkung mit keiner der Antwortmöglichkeiten vergleichbar
- ◯ Weiß nicht

NAHRUNGSERGÄNZUNGSMITTEL: NUTZEN, RISIKEN UND ERWARTUNGSHALTUNG

Wie wirken sich Nahrungsergänzungsmittel auf die Gesundheit aus? *

Bitte wählen Sie eine der folgenden Antworten:

Bitte wählen Sie nur eine der folgenden Antworten aus:

- ◯ Nahrungsergänzungsmittel sind immer vorteilhaft für die Gesundheit
- ◯ Nahrungsergänzungsmittel können sich positiv auf die Gesundheit auswirken
- ◯ Nahrungsergänzungsmittel haben keinen Einfluss auf die Gesundheit
- ◯ Nahrungsergänzungsmittel können der Gesundheit schaden
- ◯ Nahrungsergänzungsmittel sind immer gesundheitsgefährdend
- ◯ Weiß nicht

NAHRUNGSERGÄNZUNGSMITTEL: NUTZEN, RISIKEN UND ERWARTUNGSHALTUNG 2

Bitte bewerten Sie folgende Aussagen: *

Bitte wählen Sie die zutreffende Antwort für jeden Punkt aus:

	trifft zu	trifft eher zu	trifft eher nicht zu	trifft nicht zu	weiß nicht
**Die Einnahme von Nahrungsergänzungsmitteln während Zeiten körperlicher Belastung (wie Schwangerschaft, Entwicklungsunterstützung von Kindern, Krankheit, Stress etc.) ist gesundheitsfördernd**	○	○	○	○	○
**Die Einnahme von Nahrungsergänzungsmitteln ist vor allem für Veganer:innen vorteilhaft für die Gesundheit**	○	○	○	○	○
**Die Einnahme von Nahrungsergänzungsmitteln ist für schwangere Frauen vorteilhaft für die Gesundheit**	○	○	○	○	○
**Nahrungsergänzungsmittel haben eine negative Wirkung auf die Gesundheit von Mutter und Kind in der Schwangerschaft**	○	○	○	○	○

	trifft zu	trifft eher zu	trifft eher nicht zu	trifft nicht zu	weiß nicht
**Schwangere Veganerinnen sollten eher Nahrungsergänzungsmittel einnehmen als nicht vegane Schwangere**	○	○	○	○	○
**Vegan ernährte Kinder sollten keine Nahrungsergänzungsmittel einnehmen**	○	○	○	○	○
**Vegan ernährte Kinder sollten eher Nahrungsergänzungsmittel einnehmen als Kinder, die durch Mischkost ernährt werden**	○	○	○	○	○

NAHRUNGSERGÄNZUNGSMITTEL: PERSÖNLICHE AUSWAHLKRITERIEN 1

Bitte beantworten Sie folgende Fragen mit **ja** oder **nein:** *

Bitte wählen Sie die zutreffende Antwort für jeden Punkt aus:

	ja	nein
**Nehmen Sie Nahrungsergänzungsmittel ein?**	○	○
**Wurden Sie in der Vergangenheit zur Einnahme von Nahrungsergänzungsmitteln beraten?**	○	○
**Sollen Ärzt:innen, Apotheker:innen und Diätolog:innen mehr Beratung zu Nahrungsergänzungsmitteln für schwangere Veganerinnen anbieten?**	○	○
**Sollen Ärzt:innen, Apotheker:innen und Diätolog:innen mehr Beratung zu Nahrungsergänzungsmitteln für vegan ernährte Kinder anbieten?**	○	○
**Befürworten Sie die Einnahme von Nahrungsergänzungsmitteln?**	○	○
**Befürworten Sie die Einnahme von Nahrungsergänzungsmitteln (z.B. Multivitaminpräparaten, Kombinationspräparaten,**	○	○

	ja	nein
**einzelnen Nährstoffen) in der Schwangerschaft?**		
**Befürworten Sie die Einnahme von Nahrungsergänzungsmitteln (z.B. Multivitaminpräparaten, Kombinationspräparaten, einzelnen Nährstoffen) im Kindesalter (von 0-5 Jahren)?**	○	○

NAHRUNGSERGÄNZUNGSMITTEL: PERSÖNLICHE AUSWAHLKRITERIEN

Was sind Ihre wichtigsten Beweggründe für die Einnahme von Nahrungsergänzungsmitteln? (Reihen Sie bitte maximal 5 Antworten) *

Beantworten Sie diese Frage nur, wenn folgende Bedingungen erfüllt sind:
Antwort war 'ja' bei Frage ' [G9Q00001]' (Bitte beantworten Sie folgende Fragen mit ja oder nein: (Nehmen Sie Nahrungsergänzungsmittel ein?))

Alle Ihre Antworten müssen unterschiedlich sein, und müssen zugeordnet sein.
Bitte wählen Sie maximal 5 Antworten.
Bitte nummerieren Sie jede Box in der Reihenfolge Ihrer Präferenz, beginnen mit 1 bis 11
Bitte wähle Sie nicht mehr als 5 Einträge aus.

☐ Nahrungsergänzungsmittel unterstützen die Gesundheit

☐ Notwendige Nährstoffe, die durch die vegane Ernährung nicht ausreichend zugeführt werden, können ergänzt werden

☐ Mein/e Arzt/Ärztin hat mir zur Einnahme von Nahrungsergänzungsmitteln geraten

☐ Mein:e Apotheker:in hat mir zur Einnahme von Nahrungsergänzungsmitteln geraten

☐ Mein:e Diätolog:in hat mir zur Einnahme von Nahrungsergänzungsmitteln geraten

☐ Mein:e Freund:innen, Familie und/oder Arbeitskolleg:innen haben mir zur Einnahme von Nahrungsergänzungsmitteln geraten

☐ Internet und soziale Medien (Instagram, Facebook etc.) haben mich zur Einnahme motiviert

☐ Werbung hat mich zur Einnahme motiviert

☐ Fachliteratur- und Zeitschriften haben mich zur Einnahme motiviert

☐ Zeitschriften, Zeitungen und/oder Lifestyle-Magazine haben mich zur Einnahme motiviert

☐ Sonstiges

Weshalb lehnen Sie die Einnahme von Nahrungsergänzungsmitteln, in der Schwangerschaft, ab? (Mehrfachauswahl möglich) *

Beantworten Sie diese Frage nur, wenn folgende Bedingungen erfüllt sind:
Antwort war 'nein' bei Frage ' [G9Q00001]' (Bitte beantworten Sie folgende Fragen mit ja oder nein: (Befürworten Sie die Einnahme von Nahrungsergänzungsmitteln (z.B. Multivitaminpräparaten, Kombinationspräparaten, einzelnen Nährstoffen) in der Schwangerschaft?))

Wählen Sie alle zutreffenden Optionen
Bitte wählen Sie maximal 5 Antworten.
Bitte wählen Sie alle zutreffenden Antworten aus:

- ☐ Nahrungsergänzungsmittel können vom Körper schlecht aufgenommen werden
- ☐ Alle notwendigen Nährstoffe können auch durch die vegane Ernährung zugeführt werden
- ☐ Nahrungsergänzungsmittel schaden der Gesundheit der Schwangeren
- ☐ Nahrungsergänzungsmittel schaden der Gesundheit des ungeborenen Kindes
- ☐ Ich bin nicht sicher, ob Nahrungsergänzungsmittel aus veganen Quellen stammen
- ☐ Mein/e Arzt/Ärztin hat mir von der Einnahme abgeraten
- ☐ Mein:e Apotheker:in hat mir von der Einnahme abgeraten
- ☐ Mein:e Diätolog:in hat mir von der Einnahme abgeraten
- ☐ Mein:e Freund:innen, Familie und/oder Arbeitskolleg:innen haben mir von der Einnahme abgeraten
- ☐ Sonstiges

Weshalb lehnen Sie die Einnahme von Nahrungsergänzungsmitteln, von Kindern (im Alter von 0-5 Jahren), ab? (Mehrfachauswahl möglich) *

Beantworten Sie diese Frage nur, wenn folgende Bedingungen erfüllt sind:
Antwort war 'nein' bei Frage ' [G9Q00001]' (Bitte beantworten Sie folgende Fragen mit ja oder nein: (Befürworten Sie die Einnahme von Nahrungsergänzungsmitteln (z.B. Multivitaminpräparaten, Kombinationspräparaten, einzelnen Nährstoffen) im Kindesalter (von 0-5 Jahren)?))

Wählen Sie alle zutreffenden Optionen
Bitte wählen Sie maximal 5 Antworten.
Bitte wählen Sie alle zutreffenden Antworten aus:

- ☐ Nahrungsergänzungsmittel können vom kindlichen Körper schlecht aufgenommen werden
- ☐ Alle notwendigen Nährstoffe können auch durch die vegane Ernährung zugeführt werden
- ☐ Nahrungsergänzungsmittel schaden der Gesundheit des Kindes
- ☐ Ich bin nicht sicher, ob Nahrungsergänzungsmittel aus veganen Quellen stammen
- ☐ Mein/e Arzt/Ärztin hat mir von der Verabreichung von Nahrungsergänzungsmitteln an mein/e Kind/er abgeraten
- ☐ Mein:e Apotheker:in hat mir von der Verabreichung von Nahrungsergänzungsmitteln an mein/e Kind/er abgeraten
- ☐ Mein:e Diätolog:in hat mir von der Verabreichung von Nahrungsergänzungsmitteln an mein/e Kind/er abgeraten
- ☐ Mein:e Freund:innen, Familie und/oder Arbeitskolleg:innen haben mir von der Verabreichung von Nahrungsergänzungsmitteln an mein/e Kind/er abgeraten
- ☐ Sonstiges

Weshalb befürworten Sie die Einnahme von Nahrungsergänzungsmitteln, in der Schwangerschaft? (Mehrfachauswahl möglich) *

Beantworten Sie diese Frage nur, wenn folgende Bedingungen erfüllt sind:
Antwort war 'ja' bei Frage ' [G9Q00001]' (Bitte beantworten Sie folgende Fragen mit ja oder nein: (Befürworten Sie die Einnahme von Nahrungsergänzungsmitteln (z.B. Multivitaminpräparaten, Kombinationspräparaten, einzelnen Nährstoffen) in der Schwangerschaft?))

Wählen Sie alle zutreffenden Optionen
Bitte wählen Sie maximal 5 Antworten.
Bitte wählen Sie alle zutreffenden Antworten aus:

- ☐ Nahrungsergänzungsmittel unterstützen die positive Entwicklung des Kindes
- ☐ Nahrungsergänzungsmittel unterstützen die Gesundheit der Schwangeren
- ☐ Notwendige Nährstoffe die durch die vegane Ernährung nicht ausreichend zugeführt werden, können ergänzt werden
- ☐ Nahrungsergänzungsmittel versorgen Mutter und Kind mit dem zusätzlichen Bedarf an Nährstoffen in der Schwangerschaft
- ☐ Mein/e Arzt/Ärztin hat mir zur Einnahme von Nahrungsergänzungsmitteln geraten
- ☐ Mein:e Apotheker:in hat mir zur Einnahme von Nahrungsergänzungsmitteln geraten
- ☐ Mein:e Diätolog:in hat mir zur Einnahme von Nahrungsergänzungsmitteln geraten
- ☐ Mein:e Freund:innen, Familie und/oder Arbeitskolleg:innen haben mir zur Einnahme von Nahrungsergänzungsmitteln geraten
- ☐ Sonstiges

Weshalb befürworten Sie die Einnahme von Nahrungsergänzungsmitteln, von Kindern (im Alter von 0-5 Jahren)? (Mehrfachauswahl möglich) *

Beantworten Sie diese Frage nur, wenn folgende Bedingungen erfüllt sind:
Antwort war 'ja' bei Frage ' [G9Q00001]' (Bitte beantworten Sie folgende Fragen mit ja oder nein: (Befürworten Sie die Einnahme von Nahrungsergänzungsmitteln (z.B. Multivitaminpräparaten, Kombinationspräparaten, einzelnen Nährstoffen) im Kindesalter (von 0-5 Jahren)?))

Wählen Sie alle zutreffenden Optionen
Bitte wählen Sie maximal 5 Antworten.
Bitte wählen Sie alle zutreffenden Antworten aus:

- ☐ Nahrungsergänzungsmittel unterstützen die positive Entwicklung des Kindes
- ☐ Notwendige Nährstoffe, die durch die Ernährung nicht ausreichend zugeführt werden, können ergänzt werden
- ☐ Mein/e Arzt/Ärztin hat mir zur Verabreichung von Nahrungsergänzungsmitteln an mein/e Kind/er geraten
- ☐ Mein:e Apotheker:in hat mir zur Verabreichung von Nahrungsergänzungsmitteln an mein/e Kind/er geraten
- ☐ Mein:e Diätolog:in hat mir zur Verabreichung von Nahrungsergänzungsmitteln an mein/e Kind/er geraten
- ☐ Mein:e Freund:innen, Familie und/oder Arbeitskolleg:innen haben mir zur Verabreichung von Nahrungsergänzungsmitteln an mein/e Kind/er geraten
- ☐ Sonstiges

Achten Sie darauf, dass die von Ihnen eingenommen Nahrungsergänzungsmittel vegan sind? *

Beantworten Sie diese Frage nur, wenn folgende Bedingungen erfüllt sind:
Antwort war 'ja' bei Frage ' [G9Q00001]' (Bitte beantworten Sie folgende Fragen mit ja oder nein: (Befürworten Sie die Einnahme von Nahrungsergänzungsmitteln?))

Bitte wählen Sie eine der folgenden Antworten:
Bitte wählen Sie nur eine der folgenden Antworten aus:

- ◯ Ja
- ◯ Nein
- ◯ Ich nehme keine Nahrungsergänzungsmittel ein

Welche Nahrungsergänzungsmittel nehmen Sie ein? (Mehrfachauswahl möglich) *

Beantworten Sie diese Frage nur, wenn folgende Bedingungen erfüllt sind:
Antwort war 'ja' bei Frage ' [G9Q00001]' (Bitte beantworten Sie folgende Fragen mit ja oder nein: (Befürworten Sie die Einnahme von Nahrungsergänzungsmitteln?))

Wählen Sie alle zutreffenden Optionen
Bitte wählen Sie alle zutreffenden Antworten aus:

- ☐ Vitamin B12
- ☐ Folsäure
- ☐ Omega-3-Fettsäuren (DHA/EPA)
- ☐ Eisen
- ☐ Vitamin D
- ☐ Iod
- ☐ Zink
- ☐ Kalzium
- ☐ Magnesium
- ☐ Kombinationspräparate (z.B. Multivitaminpräparate)
- ☐ Keine
- ☐ Andere

Welche Nahrungsergänzungsmittel haben Sie während Ihrer/n Schwangerschaft/en eingenommen bzw. nehmen Sie während Ihrer Schwangerschaft ein? (Mehrfachauswahl möglich) *

Beantworten Sie diese Frage nur, wenn folgende Bedingungen erfüllt sind:
Antwort war 'Weiblich' *oder* 'Divers' bei Frage ' [G1Q00001]' (Geschlecht) *und* Antwort war 'Ich bin aktuell schwanger' *oder* 'Ich habe bereits eine Schwangerschaft durchlebt' *oder* 'Ich habe bereits mehrere Schwangerschaften durchlebt' *oder* 'Ich bin aktuell schwanger und habe schon ein oder mehrere Schwangerschaften durchlebt' bei Frage ' [G1Q00008]' (Schwangerschaft)

Wählen Sie alle zutreffenden Optionen
Bitte wählen Sie alle zutreffenden Antworten aus:

- ☐ Vitamin B12
- ☐ Folsäure
- ☐ Omega-3-Fettsäuren (DHA/EPA)
- ☐ Eisen
- ☐ Vitamin D
- ☐ Iod
- ☐ Zink
- ☐ Kalzium
- ☐ Magnesium
- ☐ Kombinationspräparate (z.B. Multivitaminpräparate für Schwangere)
- ☐ Keine
- ☐ Andere

Welche Nahrungsergänzungsmittel verabreichen Sie Ihren Kindern/haben Sie ihnen verabreicht (im Alter von 0 bis 5 Jahren)? (Mehrfachauswahl möglich) *

Beantworten Sie diese Frage nur, wenn folgende Bedingungen erfüllt sind:
Antwort war 'Ja' bei Frage ' [G1Q00009]' (Haben Sie Kinder?)

Wählen Sie alle zutreffenden Optionen
Bitte wählen Sie alle zutreffenden Antworten aus:

- ☐ Vitamin B12
- ☐ Folsäure
- ☐ Omega-3-Fettsäuren (DHA/EPA)
- ☐ Eisen
- ☐ Vitamin D
- ☐ Iod
- ☐ Zink
- ☐ Kalzium
- ☐ Magnesium
- ☐ Kombinationspräparate (z.B. Multivitaminpräparate für Kinder)
- ☐ Keine
- ☐ Andere

NAHRUNGSERGÄNZUNGSMITTEL: WISSENSSTAND 1

Bitte beantworten Sie nachfolgende Fragen durch Markierung der zutreffenden Antwort: *

Bitte wählen Sie die zutreffende Antwort für jeden Punkt aus:

	trifft zu	trifft eher zu	trifft eher nicht zu	trifft nicht zu	weiß nicht
**Ich bin umfassend über Nahrungsergänzungsmittel für vegan ernährte Kinder informiert**	○	○	○	○	○
**Ich bin umfassend über Nahrungsergänzungsmittel für schwangere Veganerinnen informiert**	○	○	○	○	○

NAHRUNGSERGÄNZUNGSMITTEL: WISSENSSTAND

Welche Nahrungsergänzungsmittel kann man Kindern ohne Beratung durch Arzt/Ärztin, Apotheker:in und/ oder Diätolog:in ab dem Säuglingsalter risikolos verabreichen? (Mehrfachauswahl möglich) *

Wählen Sie alle zutreffenden Optionen

Bitte wählen Sie alle zutreffenden Antworten aus:

- ☐ Vitamin B12
- ☐ Folsäure
- ☐ Omega-3-Fettsäuren (DHA/EPA)
- ☐ Eisen
- ☐ Vitamin D
- ☐ Iod
- ☐ Zink
- ☐ Kalzium
- ☐ Magnesium
- ☐ Kombinationspräparate (z.B. Multivitaminpräparate für Kinder)
- ☐ Keine
- ☐ Andere

Welche Nahrungsergänzungsmittel kann man als schwangere Veganerin ohne Beratung durch Arzt/ Ärztin, Apotheker:in und/oder Diätolog:in risikolos einnehmen? (Mehrfachauswahl möglich) *

Wählen Sie alle zutreffenden Optionen

Bitte wählen Sie alle zutreffenden Antworten aus:

- ☐ Vitamin B12
- ☐ Folsäure
- ☐ Omega-3-Fettsäuren (DHA/EPA)
- ☐ Eisen
- ☐ Vitamin D
- ☐ Iod
- ☐ Zink
- ☐ Kalzium
- ☐ Magnesium
- ☐ Kombinationspräparate (z.B. Multivitaminpräparate für Schwangere)
- ☐ Keine
- ☐ Andere

Welche Nahrungsergänzungsmittel wurden Ihnen während Ihrer/n Schwangerschaft/en von professioneller Seite aus (Arzt/Ärztin, Apotheker:innen und/oder Diätolog:innen) empfohlen? (Mehrfachauswahl möglich) *

Beantworten Sie diese Frage nur, wenn folgende Bedingungen erfüllt sind:
Antwort war 'Divers' *oder* 'Weiblich' bei Frage ' [G1Q00001]' (Geschlecht) *und* Antwort war 'Ich habe bereits mehrere Schwangerschaften durchlebt' *oder* 'Ich habe bereits eine Schwangerschaft durchlebt' *oder* 'Ich bin aktuell schwanger' *oder* 'Ich bin aktuell schwanger und habe schon ein oder mehrere Schwangerschaften durchlebt' bei Frage ' [G1Q00008]' (Schwangerschaft)

Wählen Sie alle zutreffenden Optionen
Bitte wählen Sie alle zutreffenden Antworten aus:

- ☐ Vitamin B12
- ☐ Folsäure
- ☐ Omega-3-Fettsäuren (DHA/EPA)
- ☐ Eisen
- ☐ Vitamin D
- ☐ Iod
- ☐ Zink
- ☐ Kalzium
- ☐ Magnesium
- ☐ Kombinationspräparate (z.B. Multivitaminpräparate für Schwangere)
- ☐ Keine
- ☐ Andere
- ☐ Beratung wurde nicht in Anspruch genommen

Können schwangere Veganerinnen ihren Nährstoffbedarf durch eine rein pflanzliche Ernährung decken? *

Bitte wählen Sie eine der folgenden Antworten:

Bitte wählen Sie nur eine der folgenden Antworten aus:

- ◯ Ja
- ◯ Nein
- ◯ Weiß nicht

Sind mögliche Nährstoffmängel einer veganen Ernährung in der Schwangerschaft durch Nahrungsergänzungsmittel ausgleichbar? *

Bitte wählen Sie eine der folgenden Antworten:

Bitte wählen Sie nur eine der folgenden Antworten aus:

- ◯ Ja
- ◯ Nein
- ◯ Weiß nicht

Kann der Nährstoffbedarf vegan ernährter Kinder im Alter von 0-5 Jahren durch rein pflanzliche Ernährung gedeckt werden? *

Bitte wählen Sie eine der folgenden Antworten:

Bitte wählen Sie nur eine der folgenden Antworten aus:

- ◯ Ja
- ◯ Nein
- ◯ Weiß nicht

Sind mögliche Nährstoffmängel vegan ernährter Kinder im Alter von 0-5 Jahren durch Nahrungsergänzungsmittel ausgleichbar? *

Bitte wählen Sie eine der folgenden Antworten:

Bitte wählen Sie nur eine der folgenden Antworten aus:

- ◯ Ja
- ◯ Nein
- ◯ Weiß nicht

NAHRUNGSERGÄNZUNGSMITTEL: INFORMATIONSQUELLEN

Woher beziehen Sie Ihre Informationen zur Einnahme von Nahrungsergänzungsmitteln in der Schwangerschaft? (Mehrfachauswahl möglich) *

Wählen Sie alle zutreffenden Optionen

Bitte wählen Sie alle zutreffenden Antworten aus:

- [ ] Freund:innen, Familie, Arbeitskolleg:innen
- [ ] Hausarzt:ärztin (Arzt/Ärztin für Allgemeinmedizin)
- [ ] Kinderarzt:ärztin
- [ ] Gynäkolog:in
- [ ] Apotheker:in
- [ ] Diätolog:in
- [ ] Hebamme
- [ ] Anderen Veganer:innen
- [ ] Physiotherapeut:in
- [ ] Fitnesstrainer:in
- [ ] Organisationen für Veganer:innen
- [ ] Fachliteratur/Fachzeitschriften
- [ ] Internet und soziale Medien (Instagram, Facebook etc.)
- [ ] Zeitungen, Zeitschriften und/oder Lifestyle Magazinen
- [ ] Ich informiere mich nicht über Nahrungsergänzungsmittel in der Schwangerschaft
- [ ] Werbung
- [ ] Sonstiges

Woher beziehen Sie Ihre Informationen zur Einnahme von Nahrungsergänzungsmitteln für vegan ernährte Kinder (im Alter von 0-5 Jahren)? (Mehrfachauswahl möglich) *

Wählen Sie alle zutreffenden Optionen

Bitte wählen Sie alle zutreffenden Antworten aus:

- ☐ Freund:innen, Familie, Arbeitskolleg:innen
- ☐ Hausarzt:ärztin (Arzt/Ärztin für Allgemeinmedizin)
- ☐ Kinderarzt:ärztin
- ☐ Gynäkolog:in
- ☐ Apotheker:in
- ☐ Diätolog:in
- ☐ Hebamme
- ☐ Andere Veganer:innen
- ☐ Physiotherapeut:in
- ☐ Fitnesstrainer:in
- ☐ Organisationen für Veganer:innen
- ☐ Fachliteratur/Fachzeitschriften
- ☐ Internet und soziale Medien (Instagram, Facebook etc.)
- ☐ Zeitungen, Zeitschriften und/oder Lifestyle Magazinen
- ☐ Ich informiere mich nicht über Nahrungsergänzungsmittel für Kinder
- ☐ Werbung
- ☐ Sonstiges

Wo wird man am besten zu Nahrungsergänzungsmitteln in der Schwangerschaft beraten? (Reihen Sie bitte maximal 3 Antworten) *

Alle Ihre Antworten müssen unterschiedlich sein, und müssen zugeordnet sein.
Bitte wählen Sie maximal 3 Antworten.
Bitte nummerieren Sie jede Box in der Reihenfolge Ihrer Präferenz, beginnen mit 1 bis 16
Bitte wähle Sie nicht mehr als 3 Einträge aus.

- [ ] Hausarzt:ärztin (Arzt/Ärztin für Allgemeinmedizin)
- [ ] Kinderarzt:ärztin
- [ ] Gynäkolog:in
- [ ] Apotheker:in
- [ ] Diätolog:in
- [ ] Hebamme
- [ ] Andere Veganer:innen
- [ ] Physiotherapeut:in
- [ ] Fitnesstrainer:in
- [ ] Organisationen für Veganer:innen
- [ ] Fachliteratur/Fachzeitschriften
- [ ] Internet und soziale Medien (Instagram, Facebook etc.)
- [ ] Zeitungen, Zeitschriften und/oder Lifestyle Magazinen
- [ ] Ich brauche keine Beratung zu Nahrungsergänzungsmitteln in der Schwangerschaft

Werbung

Sonstiges

Wo wird man am besten zu Nahrungsergänzungsmitteln im Kindesalter beraten? (Reihen Sie bitte maximal 3 Antworten) *

Alle Ihre Antworten müssen unterschiedlich sein, und müssen zugeordnet sein.

Bitte wählen Sie maximal 3 Antworten.

Bitte nummerieren Sie jede Box in der Reihenfolge Ihrer Präferenz, beginnen mit 1 bis 16

Bitte wähle Sie nicht mehr als 3 Einträge aus.

- [ ] Hausarzt:ärztin (Arzt/Ärztin für Allgemeinmedizin)
- [ ] Kinderarzt:ärztin
- [ ] Gynäkolog:in
- [ ] Apotheker:in
- [ ] Diätolog:in
- [ ] Hebamme
- [ ] Andere Veganer:innen
- [ ] Physiotherapeut:in
- [ ] Fitnesstrainer:in
- [ ] Organisationen für Veganer:innen
- [ ] Fachliteratur/Fachzeitschriften
- [ ] Internet und soziale Medien (Instagram, Facebook etc.)
- [ ] Zeitungen, Zeitschriften und/oder Lifestyle Magazinen
- [ ] Ich brauche keine Beratung zu Nahrungsergänzungsmitteln für Kinder
- [ ] Werbung

Sonstiges

Wie beschreiben Sie die erhaltenen Informationen zu Nahrungsergänzungsmitteln? *

Bitte wählen Sie eine der folgenden Antworten:

Bitte wählen Sie nur eine der folgenden Antworten aus:

- Vertrauenswürdig
- Hilfreich
- Unklar
- Wenig informativ
- Nicht ausreichend
- Ich erhalte keine Informationen zu Nahrungsergänzungsmitteln

Ist es leicht möglich an Informationen zu Nahrungsergänzungsmitteln für schwangere Veganerinnen zu gelangen? *

Bitte wählen Sie eine der folgenden Antworten:

Bitte wählen Sie nur eine der folgenden Antworten aus:

- Ja
- Nein
- Weiß nicht

Ist es leicht möglich an Informationen zu Nahrungsergänzungsmitteln für vegan ernährte Kinder (im Alter von 0-5 Jahren) zu gelangen? *

Bitte wählen Sie eine der folgenden Antworten:

Bitte wählen Sie nur eine der folgenden Antworten aus:

- ◯ Ja
- ◯ Nein
- ◯ Weiß nicht

Wie beurteilen Sie die verfügbare Beratung/ Informationsbereitstellung zur Dosierung von Nahrungsergänzungsmitteln in der Schwangerschaft? *

Bitte wählen Sie eine der folgenden Antworten:

Bitte wählen Sie nur eine der folgenden Antworten aus:

- ◯ Sehr gut
- ◯ Gut
- ◯ Ausreichend
- ◯ Nicht ausreichend
- ◯ Weiß nicht

Wie beurteilen Sie die verfügbare Beratung/ Informationsbereitstellung zur Dosierung von Nahrungsergänzungsmitteln im Kindesalter (von 0-5 Jahren)? *

Bitte wählen Sie eine der folgenden Antworten:

Bitte wählen Sie nur eine der folgenden Antworten aus:

- ◯ Sehr gut
- ◯ Gut
- ◯ Ausreichend
- ◯ Nicht Ausreichend
- ◯ Weiß nicht

VEGANE ERNÄHRUNG: EINFLUSSFAKTOREN

Wodurch werden/wurden Sie während Ihrer/n Schwangerschaft/en am meisten verunsichert, bei Ihrer veganen Ernährungsweise zu bleiben? *

Beantworten Sie diese Frage nur, wenn folgende Bedingungen erfüllt sind:
Antwort war 'Divers' *oder* 'Weiblich' bei Frage ' [G1Q00001]' (Geschlecht) *und* Antwort war 'Ich habe bereits mehrere Schwangerschaften durchlebt' *oder* 'Ich habe bereits eine Schwangerschaft durchlebt' *oder* 'Ich bin aktuell schwanger' *oder* 'Ich bin aktuell schwanger und habe schon ein oder mehrere Schwangerschaften durchlebt' bei Frage ' [G1Q00008]' (Schwangerschaft)

Wählen Sie alle zutreffenden Optionen
Bitte wählen Sie maximal 3 Antworten.
Bitte wählen Sie alle zutreffenden Antworten aus:

- ☐ Soziales Umfeld (Familie, Freund:innen, Arbeitskolleg:innen)
- ☐ Behandelnde Ärzt:innen
- ☐ Beratung in der Apotheke
- ☐ Diätolog:in
- ☐ Hebamme
- ☐ Internet und soziale Medien (Instagram, Facebook etc.)
- ☐ Fachliteratur/Fachzeitschriften
- ☐ Zeitungen, Zeitschriften und/oder Lifestyle Magazine
- ☐ Allgemeiner Mangel an vertrauensvoller Informationsbereitstellung zum Thema vegane Ernährung und Nahrungsergänzungsmittel in der Schwangerschaft
- ☐ Ich werde/wurde nicht verunsichert
- ☐ Sonstiges

Haben Sie Ihren:Ihre Gynäkolog:in in der Schwangerschaft darüber informiert, dass Sie Veganerin sind? *

Beantworten Sie diese Frage nur, wenn folgende Bedingungen erfüllt sind:
Antwort war 'Divers' *oder* 'Weiblich' bei Frage ' [G1Q00001]' (Geschlecht) *und* Antwort war 'Ich habe bereits mehrere Schwangerschaften durchlebt' *oder* 'Ich habe bereits eine Schwangerschaft durchlebt' *oder* 'Ich bin aktuell schwanger' *oder* 'Ich bin aktuell schwanger und habe schon ein oder mehrere Schwangerschaften durchlebt' bei Frage ' [G1Q00008]' (Schwangerschaft)

Bitte wählen Sie eine der folgenden Antworten:
Bitte wählen Sie nur eine der folgenden Antworten aus:

- Ja
- Nein

Wurden Sie von Ihrem:Ihrer Gynäkolog:in zur Einnahme von Nahrungsergänzungsmitteln während der Schwangerschaft beraten? *

Beantworten Sie diese Frage nur, wenn folgende Bedingungen erfüllt sind:
Antwort war 'Weiblich' *oder* 'Divers' bei Frage ' [G1Q00001]' (Geschlecht) *und* Antwort war 'Ich habe bereits mehrere Schwangerschaften durchlebt' *oder* 'Ich habe bereits eine Schwangerschaft durchlebt' *oder* 'Ich bin aktuell schwanger' *oder* 'Ich bin aktuell schwanger und habe schon ein oder mehrere Schwangerschaften durchlebt' bei Frage ' [G1Q00008]' (Schwangerschaft)

Bitte wählen Sie eine der folgenden Antworten:
Bitte wählen Sie nur eine der folgenden Antworten aus:

- Ja, ich wurde umfassend beraten
- Ich wurde kaum beraten
- Ich wollte nicht beraten werden
- Nein, ich wurde gar nicht beraten

Wie isst/essen Ihr/e Kind/er? *

Beantworten Sie diese Frage nur, wenn folgende Bedingungen erfüllt sind:
Antwort war 'Ja' bei Frage ' [G1Q00009]' (Haben Sie Kinder?)

Wählen Sie alle zutreffenden Optionen
Bitte wählen Sie alle zutreffenden Antworten aus:

- ☐ Mein/e Kind/er isst/essen rein pflanzlich
- ☐ Mein/e Kind/er isst/essen auch Milchprodukte, Eier und Honig
- ☐ Mein/e Kind/er isst/essen auch Fisch
- ☐ Mein/e Kind/er isst/essen auch Fleisch

Weshalb ernähren Sie Ihr/e Kind/er nicht ausschließlich vegan? (Mehrfachauswahl möglich) *

Beantworten Sie diese Frage nur, wenn folgende Bedingungen erfüllt sind:
Antwort war 'Ja' bei Frage ' [G1Q00009]' (Haben Sie Kinder?) *und* Antwort war 'Mein/e Kind/er isst/essen auch Fleisch' *oder* 'Mein/e Kind/er isst/essen auch Fisch' *oder* 'Mein/e Kind/er isst/essen auch Milchprodukte, Eier und Honig' bei Frage ' [G14Q00004]' (Wie isst/essen Ihr/e Kind/er?)

Wählen Sie alle zutreffenden Optionen
Bitte wählen Sie alle zutreffenden Antworten aus:

- ☐ Familie, Freund:innen und Arbeitskolleg:innen haben mir davon abgeraten
- ☐ Der/Die praktische Arzt/Ärztin hat mir davon abgeraten
- ☐ Der/Die Kinderarzt:ärztin hat mir davon abgeraten
- ☐ In der Apotheke wurde mir davon abgeraten
- ☐ Mein:e Diätolog:in hat mir davon abgeraten
- ☐ Ich befürchte, die vegane Ernährung könnte die Entwicklung meines Kindes negativ beeinflussen
- ☐ Ich gelange an keine vertrauenswürdigen Informationen, die eine ausgewogene vegane Ernährung für Kinder erklären
- ☐ Ich gelange an keine vertrauenswürdigen Informationen, für die richtige Dosierungs- und Darreichungsform (Tropfen, Kapseln, Tabletten, Saft etc.), von Nahrungsergänzungsmitteln für Kinder
- ☐ In den sozialen Medien (Instagram, Facebook etc.) wird/wurde davon abgeraten
- ☐ In Fachliteratur/Fachzeitschriften wird/wurde davon abgeraten
- ☐ In Zeitungen, Zeitschriften und/oder Lifestyle-Magazinen wird/wurde davon abgeraten
- ☐ Mein Kind verlangt nach nicht-veganen Nahrungsmitteln
- ☐ Sonstiges

Was verunsichert Sie am meisten/hat Sie am meisten verunsichert, Ihr/e Kind/er vegan zu ernähren? *

Beantworten Sie diese Frage nur, wenn folgende Bedingungen erfüllt sind:
Antwort war 'Ja' bei Frage ' [G1Q00009]' (Haben Sie Kinder?)

Wählen Sie alle zutreffenden Optionen
Bitte wählen Sie maximal 3 Antworten.
Bitte wählen Sie alle zutreffenden Antworten aus:

- ☐ Soziales Umfeld (Familie, Freund:innen, Arbeitskolleg:innen)
- ☐ Behandelnde Ärzt:innen
- ☐ Beratung in der Apotheke
- ☐ Diätolog:in
- ☐ Allgemeiner Mangel an vertrauenswürdigen Informationen, die eine ausgewogene vegane Ernährung für Kinder erklären
- ☐ Allgemeiner Mangel an vertrauenswürdigen Informationen, für die richtige Dosierungs- und Darreichungsform (Tropfen, Kapseln, Tabletten, Saft etc.), von Nahrungsergänzungsmitteln für Kinder
- ☐ Fachliteratur/Fachzeitschriften
- ☐ Internet und soziale Medien (Instagram, Facebook etc.)
- ☐ Zeitungen, Zeitschriften und/oder Lifestyle-Magazine
- ☐ Ich werde/wurde nicht verunsichert
- ☐ Sonstiges

Haben Sie Ihre:n Kinderarzt:ärztin darüber informiert, dass Sie Ihr/e Kind/er vegan ernähren? *

Beantworten Sie diese Frage nur, wenn folgende Bedingungen erfüllt sind:
Antwort war 'Ja' bei Frage ' [G1Q00009]' (Haben Sie Kinder?) *und* Antwort war 'Mein/e Kind/er isst/essen rein pflanzlich' bei Frage ' [G14Q00004]' (Wie isst/essen Ihr/e Kind/er?)

Bitte wählen Sie eine der folgenden Antworten:
Bitte wählen Sie nur eine der folgenden Antworten aus:

- ◯ Ja
- ◯ Nein
- ◯ Ich ernähre mein Kind nicht ausschließlich vegan

Wurden Sie von Ihrem:Ihrer Kinderarzt:ärztin zur Einnahme von Nahrungsergänzungsmitteln für Kinder beraten? *

Beantworten Sie diese Frage nur, wenn folgende Bedingungen erfüllt sind:
Antwort war 'Ja' bei Frage ' [G1Q00009]' (Haben Sie Kinder?)

Bitte wählen Sie eine der folgenden Antworten:
Bitte wählen Sie nur eine der folgenden Antworten aus:

- ◯ Ja, ich wurde umfassend beraten
- ◯ Ich wurde kaum beraten
- ◯ Ich wollte nicht beraten werden
- ◯ Nein, ich wurde gar nicht beraten

NAHRUNGSERGÄNZUNGSMITTEL: MANGEL UND FALSCHE DOSIERUNG (14) 1

Beurteilen Sie das Gesundheitsrisiko eines/r Mangels/ Unterdosierung bzw. einer Überdosierung der nachstehenden Nährstoffe/Nahrungsergänzungsmittel aufgrund einer veganen Ernährung für **Schwangere.** (1 = Kein Risiko, 5 = hohes Risiko) *

Bitte wählen Sie die zutreffende Antwort für jeden Punkt aus:

	Risiko bei Mangel/ Unterdosierung							Risiko bei Überdosierung					
	1	2	3	4	5	weiß nicht		1	2	3	4	5	weiß nicht
**Vitamin B12**	◯	◯	◯	◯	◯	◯		◯	◯	◯	◯	◯	◯
**Folsäure**	◯	◯	◯	◯	◯	◯		◯	◯	◯	◯	◯	◯
**Omega-3-Fettsäuren (DHA/EPA)**	◯	◯	◯	◯	◯	◯		◯	◯	◯	◯	◯	◯
**Eisen**	◯	◯	◯	◯	◯	◯		◯	◯	◯	◯	◯	◯
**Vitamin D**	◯	◯	◯	◯	◯	◯		◯	◯	◯	◯	◯	◯
**Iod**	◯	◯	◯	◯	◯	◯		◯	◯	◯	◯	◯	◯
**Zink**	◯	◯	◯	◯	◯	◯		◯	◯	◯	◯	◯	◯
**Kalzium**	◯	◯	◯	◯	◯	◯		◯	◯	◯	◯	◯	◯
**Magnesium**	◯	◯	◯	◯	◯	◯		◯	◯	◯	◯	◯	◯

NAHRUNGSERGÄNZUNGSMITTEL: MANGEL UND FALSCHE DOSIERUNG

Beurteilen Sie das Gesundheitsrisiko eines/r Mangels/ Unterdosierung bzw. einer Überdosierung der nachstehenden Nährstoffe/Nahrungsergänzungsmittel aufgrund einer veganen Ernährung von **Säuglingen und Kindern (bis zu einem Alter von 5 Jahren).** (1 = Kein Risiko, 5 = hohes Risiko) *

Bitte wählen Sie die zutreffende Antwort für jeden Punkt aus:

	Risiko bei Mangel/ Unterdosierung						Risiko bei Überdosierung					
	1	2	3	4	5	weiß nicht	1	2	3	4	5	weiß nicht
**Vitamin B12**	◯	◯	◯	◯	◯	◯	◯	◯	◯	◯	◯	◯
**Folsäure**	◯	◯	◯	◯	◯	◯	◯	◯	◯	◯	◯	◯
**Omega-3-Fettsäuren (DHA/EPA)**	◯	◯	◯	◯	◯	◯	◯	◯	◯	◯	◯	◯
**Eisen**	◯	◯	◯	◯	◯	◯	◯	◯	◯	◯	◯	◯
**Vitamin D**	◯	◯	◯	◯	◯	◯	◯	◯	◯	◯	◯	◯
**Iod**	◯	◯	◯	◯	◯	◯	◯	◯	◯	◯	◯	◯
**Zink**	◯	◯	◯	◯	◯	◯	◯	◯	◯	◯	◯	◯
**Kalzium**	◯	◯	◯	◯	◯	◯	◯	◯	◯	◯	◯	◯
**Magnesium**	◯	◯	◯	◯	◯	◯	◯	◯	◯	◯	◯	◯

NAHRUNGSERGÄNZUNGSMITTEL: APPLIKATION UND COMPLIANCE (15) 1

Was ist/war Ihr größtes Bedenken bei der Einnahme von Nahrungsergänzungsmitteln in der Schwangerschaft? *

Beantworten Sie diese Frage nur, wenn folgende Bedingungen erfüllt sind:
Antwort war 'Weiblich' *oder* 'Divers' bei Frage ' [G1Q00001]' (Geschlecht) *und* Antwort war 'Ich bin aktuell schwanger' *oder* 'Ich habe bereits eine Schwangerschaft durchlebt' *oder* 'Ich habe bereits mehrere Schwangerschaften durchlebt' *oder* 'Ich bin aktuell schwanger und habe schon ein oder mehrere Schwangerschaften durchlebt' bei Frage ' [G1Q00008]' (Schwangerschaft)

Bitte wählen Sie eine der folgenden Antworten:
Bitte wählen Sie nur eine der folgenden Antworten aus:

- ◯ Meinem ungeborenen Kind wird durch falsche Dosierung geschadet
- ◯ Mein ungeborenes Kind und ich werden durch die Zufuhr von Nahrungsergänzungsmitteln gesundheitlich belastet
- ◯ Mein Körper wird durch das Nahrungsergänzungsmittel mehr belastet als unterstützt
- ◯ Die Gesundheit meines ungeborenen Kindes wird durch die Einnahme von Nahrungsergänzungsmitteln belastet (auch bei richtiger Dosierung)
- ◯ Ich habe keine Bedenken, da Nahrungsergänzungsmittel sicher sind
- ◯ Sonstiges

Was ist/war Ihr größtes Bedenken bei der Verabreichung von Nahrungsergänzungsmitteln an Ihr/e Kind/er (im Alter von 0-5 Jahren)? *

Beantworten Sie diese Frage nur, wenn folgende Bedingungen erfüllt sind:
Antwort war 'Ja' bei Frage ' [G1Q00009]' (Haben Sie Kinder?)

Bitte wählen Sie eine der folgenden Antworten:
Bitte wählen Sie nur eine der folgenden Antworten aus:

- ◯ Meinem Kind wird durch falsche Dosierung geschadet
- ◯ Mein Kind wird durch die Zufuhr von Nahrungsergänzungsmitteln gesundheitlich belastet (auch bei richtiger Dosierung)
- ◯ Ich habe keine Bedenken, da Nahrungsergänzungsmittel sicher sind
- ◯ Sonstiges

Welche Verabreichungsform/en von Nahrungsergänzungsmitteln ist/sind am ehesten für Kinder geeignet? (Mehrfachauswahl möglich) *

Wählen Sie alle zutreffenden Optionen
Bitte wählen Sie alle zutreffenden Antworten aus:

- ☐ Tropfen
- ☐ Saft/Sirup
- ☐ Tablette ganz
- ☐ Tablette zu Pulver zerstoßen (und in Wasser, Fruchtsaft, Joghurt etc. gelöst)
- ☐ Kapsel ganz
- ☐ Kapselinhalt geleert (und in Wasser, Fruchtsaft, Joghurt etc. gelöst)
- ☐ Nahrungsergänzungsmittel in Form einer "Süßigkeit" (z.B. Gummidrops, Toffees)
- ☐ Zahnpaste/Zahncreme mit Nährstoffen angereichert (z.B. mit Vitamin B12)
- ☐ Mundspray (z.B. mit Vitamin D, Vitamin B12)
- ☐ Keine

NAHRUNGSERGÄNZUNGSMITTEL: APPLIKATION UND

COMPLIANCE 3

Bitte beantworten Sie nachfolgende Fragen durch Markierung der zutreffenden Antwort: *

Beantworten Sie diese Frage nur, wenn folgende Bedingungen erfüllt sind:
Antwort war 'Ja' bei Frage ' [G1Q00009]' (Haben Sie Kinder?)

Bitte wählen Sie die zutreffende Antwort für jeden Punkt aus:

	immer	meistens	selten	nie	keine Angabe
**Nehmen/nahmen Sie Nahrungsergänzungsmittel in der Schwangerschaft ein?**	○	○	○	○	○
**Verabreichen/ Verabreichten Sie Ihren Kindern Nahrungsergänzungsmittel?**	○	○	○	○	○
**Halten/Hielten Sie bei der Verabreichung von Nahrungsergänzungsmitteln an Ihre Kinder die empfohlene Dosierung ein?**	○	○	○	○	○
**Ist die Verabreichung von Nahrungsergänzungsmitteln (als Tropfen, Tabletten, Kapsel, Säfte etc.) an Ihr/e Kind/er schwer umsetzbar?**	○	○	○	○	○
**Sind/waren Sie sich bei der Verabreichung von Nahrungsergänzungsmitteln an Ihre Kinder verunsichert?**	○	○	○	○	○

NAHRUNGSERGÄNZUNGSMITTEL: APPLIKATION UND COMPLIANCE 4

Ab welchem Alter haben Sie Ihrem Kind Nahrungsergänzungsmittel verabreicht? *

Beantworten Sie diese Frage nur, wenn folgende Bedingungen erfüllt sind:
Antwort war 'Ja' bei Frage ' [G1Q00009]' (Haben Sie Kinder?) *und* Antwort war 'Mein/e Kind/er isst/essen rein pflanzlich' bei Frage ' [G14Q00004]' (Wie isst/essen Ihr/e Kind/er?) *und* Antwort war 'selten' *oder* 'meistens' *oder* 'immer' bei Frage ' [G18Q00001]' (Bitte beantworten Sie nachfolgende Fragen durch Markierung der zutreffenden Antwort: (Verabreichen/Verabreichten Sie Ihren Kindern Nahrungsergänzungsmittel?)) *und* Antwort war '1' bei Frage ' [G1Q00011]' (Wie viele Kinder haben Sie?)

Bitte wählen Sie eine der folgenden Antworten:
Bitte wählen Sie nur eine der folgenden Antworten aus:

- ◯ Ab der Geburt
- ◯ Ab Beginn der Beikost
- ◯ Nach einem Monat
- ◯ Nach 1-3 Monaten
- ◯ Nach 3-6 Monaten
- ◯ Nach 6-12 Monaten
- ◯ Nach 12-24 Monaten
- ◯ Später

Ab welchem Alter haben Sie Ihren Kindern Nahrungsergänzungsmittel verabreicht? (Mehrfachauswahl möglich) *

Beantworten Sie diese Frage nur, wenn folgende Bedingungen erfüllt sind:
Antwort war 'Ja' bei Frage ' [G1Q00009]' (Haben Sie Kinder?) *und* Antwort war 'Mein/e Kind/er isst/essen rein pflanzlich' bei Frage ' [G14Q00004]' (Wie isst/essen Ihr/e Kind/er?) *und* Antwort war 'immer' *oder* 'meistens' *oder* 'selten' bei Frage ' [G18Q00001]' (Bitte beantworten Sie nachfolgende Fragen durch Markierung der zutreffenden Antwort: (Verabreichen/Verabreichten Sie Ihren Kindern Nahrungsergänzungsmittel?)) *und* Antwort war '2' *oder* '3' bei Frage ' [G1Q00011]' (Wie viele Kinder haben Sie?)

Wählen Sie alle zutreffenden Optionen
Bitte wählen Sie alle zutreffenden Antworten aus:

- ☐ Ab der Geburt
- ☐ Ab Beginn der Beikost
- ☐ Nach einem Monat
- ☐ Nach 1-3 Monaten
- ☐ Nach 6-12 Monaten
- ☐ Nach 12-24 Monaten
- ☐ Später

NAHRUNGSERGÄNZUNGSMITTEL: APPLIKATION UND COMPLIANCE 5

Bitte bewerten Sie folgende Fragen/Aussagen: *

Bitte wählen Sie die zutreffende Antwort für jeden Punkt aus:

	ja	eher ja	eher nein	nein	weiß nicht
**Ist die regelmäßige Einnahme von Nahrungsergänzungsmitteln in der Schwangerschaft wichtig für deren Wirksamkeit?**	○	○	○	○	○
**Ist die regelmäßige Einnahme von Nahrungsergänzungsmitteln im Kindesalter (von 0-5 Jahren) wichtig für deren Wirksamkeit?**	○	○	○	○	○
**Genaue Dosierungsempfehlungen für Nahrungsergänzungsmittel für schwangere Veganerinnen sind für mich eine Voraussetzung für deren Einnahme in der Schwangerschaft**	○	○	○	○	○
**Genaue Dosierungsempfehlungen für Nahrungsergänzungsmittel für vegan ernährte Kinder (im Alter von 0-5 Jahren) sind für mich eine Voraussetzung für deren Verabreichung**	○	○	○	○	○

	ja	eher ja	eher nein	nein	weiß nicht
**im Kindesalter**					

NAHRUNGSERGÄNZUNGSMITTEL: APPLIKATION UND COMPLIANCE (15) 2

Wie beurteilen/beurteilten Sie die Verabreichung von Nahrungsergänzungsmitteln an Ihr/e Kind/er (im Alter von 0-5 Jahren)? *

Beantworten Sie diese Frage nur, wenn folgende Bedingungen erfüllt sind:
Antwort war 'Ja' bei Frage ' [G1Q00009]' (Haben Sie Kinder?)

Bitte wählen Sie die zutreffende Antwort für jeden Punkt aus:

	unkompliziert	eher unkompliziert	eher kompliziert	kompliziert	weiß nicht
**Tropfen**	○	○	○	○	○
**Saft/Sirup**	○	○	○	○	○
**Tablette ganz**	○	○	○	○	○
**Tablette zu Pulver zerstoßen (und in Wasser, Fruchtsaft, Joghurt etc. gelöst)**	○	○	○	○	○
**Kapsel ganz**	○	○	○	○	○
**Kapselinhalt geleert (und in Wasser, Fruchtsaft, Joghurt etc. gelöst)**	○	○	○	○	○
**Nahrungsergänzungsmittel in Form einer "Süßigkeit" (z.B. Gummidrops, Toffees)**	○	○	○	○	○
**Zahnpaste/Zahncreme mit Nährstoffen angereichert (z.B. mit Vitamin B12)**	○	○	○	○	○
**Mundspray (z.B. mit Vitamin D, Vitamin B12)**	○	○	○	○	○

Vielen Dank für Ihre Umfrageteilnahme! Bei Fragen steht Ihnen Wolfgang Huber-Schneider (Studiendurchführung) unter folgendem Kontakt zur Verfügung:

a00225229@unet.univie.ac.at

Senden Sie Ihre Umfage ein.
Vielen Dank für die Beantwortung des Fragebogens.
