# Supplementary material for: Can Dietary Supplements Be Linked to a Vegan Diet and Health Risk Modulation During Vegan Pregnancy, Infancy, and Early Childhood? The VedieS Study Protocol for an Explorative, Quantitative, Cross-Sectional Study
Source: Int J Environ Res Public Health. 2025 Jul 31;22(8):1210. doi: 10.3390/ijerph22081210 (PMC12386434; doi:10.3390/ijerph22081210)
Supplement: Supplementary file 1 [file ijerph-22-01210-s001.zip › S2_3677589.pdf]

Expert:innenbefragung zu veganer Ernährung sowie zur Einnahme von Nahrungsergänzungsmitteln während der Schwangerschaft und im Säuglings- und frühen Kindesalter

Teilnehmer:inneninformation und Einwilligungserklärung zur Teilnahme an der Studie:

Arbeitstitel der Studie:

Können Nahrungsergänzungsmittel mit veganer Ernährung und einer Modulation des Gesundheitsrisikos während Schwangerschaft, Säuglings- und frühen Kindesalters in Verbindung gebracht werden?

Sehr geehrte Teilnehmerin, sehr geehrter Teilnehmer,

wir laden Sie ein, an der oben genannten Studie teilzunehmen.

Ihre Teilnahme an dieser Studie erfolgt freiwillig. Sie können jederzeit, ohne Angabe von Gründen, Ihre Teilnahme im Verlauf der Befragung beenden und somit Ihre Teilnahmebereitschaft an der Studie, durch Abbruch der Befragung, widerrufen. Die Ablehnung der Teilnahme oder ein vorzeitiges Ausscheiden aus dieser Studie hat keine nachteiligen Folgen für Sie. Diese Art von Studien ist notwendig, um verlässliche neue wissenschaftliche Forschungsergebnisse zu gewinnen. Unverzichtbare Voraussetzung für die Durchführung von Studien ist jedoch, dass Sie Ihr Einverständnis zur Teilnahme an dieser Studie erklären. Es werden KEINE sensiblen Daten wie Name, E-

Mail-Adresse oder IP-Adresse erhoben. Die Teilnahme an der Befragung wird **ca. 20 Minuten** Ihrer Zeit in Anspruch nehmen. Bitte lesen Sie den folgenden Text sorgfältig durch und zögern Sie nicht, Fragen zu stellen. Bei Rückfragen wenden Sie sich bitte an den angegebenen Kontakt des **studiendurchführenden Doktoratsstudenten Wolfgang Huber-Schneider** (Kontakt ganz unten im Text).

Bitte bestätigen Sie die Einwilligungserklärung nur (Bestätigung = Anklicken von "Weiter" - der Beginn der Befragung gilt als Bestätigung der Einwilligungserklärung):

- wenn Sie Art und Ablauf der Studie vollständig verstanden haben,
- wenn Sie bereit sind, der Teilnahme zuzustimmen und
- wenn Sie sich über Ihre Rechte als Teilnehmer:in an dieser Studie im Klaren sind.

- **Was ist der Zweck der Studie?**

Das Ziel der Studie ist es, Zusammenhänge von Beratung durch medizinische Expert:innen (z.B. durch Arzt/Ärztin, Apotheker:innen) und anderen Einflussfaktoren (z.B. soziales Umfeld, soziale Medien), mit der veganen Ernährung und der Einnahme von Nahrungsergänzungsmitteln in Schwangerschaft, Säuglingsalter und früher Kindheit (bis zu einem Alter von 5 Jahren) von Veganer:innen zu klären. Somit können mögliche gesundheitliche Risiken für betreffende Veganer:innen in Erfahrung gebracht und präventiv minimiert werden. Bitte helfen Sie mit Ihrer Teilnahme an der Befragung mit, Informationsmöglichkeiten und Risikoprävention für die Gesundheit von Veganer:innen zu optimieren. Die Befragung wird in Zusammenhang mit einem PhD Projekt durchgeführt.

- **Wie läuft die Studie ab?**

Der Fragebogen richtet sich an medizinische Expert:innen (Gynäkolog:innen, Pädiater:innen, Allgemeinmediziner:innen, Apotheker:innen, Diätolog:innen). Fragen zur veganen Ernährung und Einnahme von Nahrungsergänzungsmitteln in Schwangerschaft, Säuglingsalter und früher Kindheit (bis zu einem Alter von 5 Jahren) werden gestellt. Die Beantwortung des Fragenbogens wird ca. **20 Minuten in Anspruch nehmen.**

- **Worin liegt der Nutzen einer Teilnahme an der Studie?**

Es ist nicht zu erwarten, dass Sie aus Ihrer Teilnahme einen direkten, persönlichen Nutzen ziehen werden, allerdings leisten Sie durch die Beantwortung des Fragebogens einen wichtigen Beitrag, mögliche gesundheitliche Risiken für schwangere Veganerinnen und vegan ernährte Kinder präventiv zu minimieren. Aus wissenschaftlicher Sicht soll, durch Erforschung von Informationsquellen und deren Einflussnahme auf die Ernährung und Einnahme von Nahrungsergänzungsmitteln von und für genannte Veganer:innen, die Informationsversorgung und Informationsqualität für Veganer:innen optimiert werden.

- **Gibt es Risiken bei der Durchführung der Studie und ist mit Beschwerden oder anderen Begleiterscheinungen zu rechnen?**

Es ist nicht zu erwarten, dass die Teilnahme an der Studie mit Unannehmlichkeiten oder Risiken verbunden ist.

Einschlusskriterien für die Teilnahme an der Studie:

1. Gynäkolog:innen, Pädiater:innen, Allgemeinmediziner:innen, Pharmazeut:innen, Diätolog:innen mit oder ohne Beratungsschwerpunkt zu veganer Ernährung

2. Bestätigung der Teilnehmer:inneninformation und Einverständniserklärung

Ausschlusskriterien für die Teilnahme an der Studie:

1. Andere medizinische Expert:innen, als in Einschlusskriterien genannt

2. Keine Bestätigung der Teilnehmer:inneninformation und Einverständniserklärung

Für die Teilnahme an der Studie müssen beide Einschlusskriterien erfüllt sein. Wenn nur ein Ausschlusskriterium erfüllt ist, kann die Teilnahme an der Studie nicht erfolgen.

- **Hat die Teilnahme an der Studie sonstige Auswirkungen auf die Lebensführung und welche Verpflichtungen ergeben sich daraus?**

Nein, Sie können jederzeit an der Befragung teilnehmen und diese zu jedem beliebigen Zeitpunkt, ohne Konsequenzen oder sonstigen Auswirkungen, abbrechen.

- **Was ist zu tun beim Auftreten von Beschwerdesymptomen, unerwünschten Begleiterscheinungen und/ oder Verletzungen?**

Da es sich um eine Befragung handelt, ist mit keinen Beschwerdesymptomen zu rechnen.

- **Wann wird die Studie vorzeitig beendet?**

Sie können jederzeit, auch ohne Angabe von Gründen, Ihre Teilnahmebereitschaft widerrufen und aus der Studie ausscheiden (indem Sie die Befragung abbrechen), ohne dass dadurch irgendwelche Nachteile für Sie entstehen.

- **In welcher Weise werden die im Rahmen dieser Studie gesammelten Daten verwendet?**

Innerhalb Ihrer Teilnahme an der Befragung ist es nicht erforderlich, persönliche Daten anzugeben. Die Beantwortung der Fragen erfolgt völlig anonym. Anonyme, gesammelte Daten werden ausschließlich für statistische Zwecke gespeichert. Es werden ausnahmslos keine persönlichen Daten (z.B. Name, IP-Adresse, E-Mail-Adresse) erfragt oder gespeichert. Keine Rückschlüsse auf Ihre Person sind möglich.

- **Entstehen für die Teilnehmer:innen Kosten? Gibt es einen Kostenersatz oder eine Vergütung?**

Durch die Teilnahme an dieser Studie entstehen für Sie keinerlei Kosten. Es gibt keinen Kostenersatz und keine Vergütung für Teilnehmer:innen der Befragung/Studie.

- **Möglichkeit zur Diskussion weiterer Fragen**

Für weitere Fragen im Zusammenhang mit dieser Studie steht Ihnen Wolfgang Huber-Schneider gern zur Verfügung. Auch Fragen, die Ihre Rechte als Teilnehmer:in betreffen, werden Ihnen selbstverständlich beantwortet. Sobald allgemeine Ergebnisse dieser Studie vorliegen, können Sie ebenfalls darüber informiert werden, falls Sie dies wünschen.

- **Kontakt - Studienteam/Doktoratsstudent**

Die Befragung wird im Zuge der Dissertation von **Wolfgang Huber-Schneider** am **Department für Ernährungswissenschaften der Universität Wien in Kooperation mit der AGES (Österreichische Agentur für Gesundheit und Ernährungssicherheit)** durchgeführt. Bei Fragen kontaktieren Sie:

Doktoratsstudent: Wolfgang Huber-Schneider, a00225229@unet.univie.ac.at

Studienleitung: Univ.-Prof. Mag. Dr. Karl-Heinz Wagner (Department für Ernährungswissenschaften Universität Wien), Univ.-Doz.in Mag.a Dr.in Ingrid Kiefer (AGES), Mag. pharm. Wolfgang Huber-Schneider (Doktoratsstudent)
Prüfarzt: Univ.-Prof. Dr. Daniel König (Institut für Sportwissenschaft Universität Wien)

Sobald Sie auf **"Weiter"** klicken, stimmen Sie automatisch Ihrer Teilnahme zur Befragung zu.

In dieser Umfrage sind 54 Fragen enthalten.

Allgemeines

Geschlecht *

Bitte wählen Sie eine der folgenden Antworten:
Bitte wählen Sie nur eine der folgenden Antworten aus:

- ◯ Weiblich
- ◯ Männlich
- ◯ Divers

Alter (in Jahren) *

Bitte wählen Sie eine der folgenden Antworten:
Bitte wählen Sie nur eine der folgenden Antworten aus:

- ◯ Unter 30
- ◯ 31-40
- ◯ 41-50
- ◯ 51-60
- ◯ 61 oder älter

In welchem Land arbeiten Sie? *

Bitte wählen Sie eine der folgenden Antworten:
Bitte wählen Sie nur eine der folgenden Antworten aus:

- ◯ Österreich
- ◯ Deutschland
- ◯ Schweiz
- ◯ Italien
- ◯ Anderes EU-Land
- ◯ Sonstiges

Wo ist der Ort Ihrer Berufsausübung (bitte geben Sie die Postleitzahl an)? *

Bitte geben Sie Ihre Antwort hier ein:

Welcher Expert:innengruppe gehören Sie an? *

Bitte wählen Sie eine der folgenden Antworten:
Bitte wählen Sie nur eine der folgenden Antworten aus:

- Facharzt:ärztin für Gynäkologie und Geburtshilfe
- Facharzt:ärztin für Kinder- und Jugendheilkunde
- (Fach-)arzt:ärztin für Allgemeinmedizin
- Pharmazeut:in
- Diätolog:in/Diätassistent:in

Ich bin *

Beantworten Sie diese Frage nur, wenn folgende Bedingungen erfüllt sind:
Antwort war 'Österreich' bei Frage ' [G1Q00003]' (In welchem Land arbeiten Sie?) *und* Antwort war '(Fach-)arzt:ärztin für Allgemeinmedizin' *oder* 'Facharzt:ärztin für Kinder- und Jugendheilkunde' *oder* 'Facharzt:ärztin für Gynäkologie und Geburtshilfe' bei Frage ' [G1Q00005]' (Welcher Expert:innengruppe gehören Sie an?)

Bitte wählen Sie eine der folgenden Antworten:
Bitte wählen Sie nur eine der folgenden Antworten aus:

- Kassenarzt:ärztin
- Wahlarzt:ärztin
- ausschließlich im Krankenhaus als Arzt:ärztin tätig
- Sonstiges

Gesundheit 1

Was bedeutet Gesundheit aus persönlicher Sicht für Sie? (Reihen Sie bitte maximal 3 Antworten) *

Alle Ihre Antworten müssen unterschiedlich sein, und müssen zugeordnet sein.
Bitte wählen Sie maximal 3 Antworten.
Bitte nummerieren Sie jede Box in der Reihenfolge Ihrer Präferenz, beginnen mit 1 bis 8
Bitte wähle Sie nicht mehr als 3 Einträge aus.

[ ] Ein Zustand, völligen psychischen, physischen und sozialen Wohlbefindens

[ ] Das Freisein von Krankheit und Gebrechen

[ ] Sich wohlzufühlen - auch ohne Diagnosen und Untersuchungsergebnisse

[ ] An keiner diagnostizierten Krankheit zu leiden

[ ] Den Alltag ohne Einschränkungen leben zu können

[ ] Psychisch und physisch belastbar zu sein

[ ] Glücklich und beschwerdefrei zu sein

[ ] Überdurchschnittlich leistungsfähig zu sein

Gesundheit 2

Was bedeutet Gesundheit in der Schwangerschaft vor allem für Sie? *

Bitte wählen Sie eine der folgenden Antworten:
Bitte wählen Sie nur eine der folgenden Antworten aus:

- ◯ Eine komplikationsfreie Schwangerschaft
- ◯ Eine Schwangerschaft, in der sich das Kind durch Bereitstellung aller benötigten Nährstoffe ideal entwickelt
- ◯ Eine Schwangerschaft, in der das Kind den Entwicklungsfortschritten (lt. ärztlichen Untersuchungen) bestmöglich entspricht
- ◯ Eine Schwangerschaft, in der man sich wohl fühlt - auch ohne ärztliche Bestätigung des Gesundheitszustandes
- ◯ Eine Schwangerschaft, die zeitlich wie erwartet und planmäßig verläuft

Gesundheit 3

Was bedeutet Gesundheit im Kindesalter vor allem für Sie? *

Bitte wählen Sie eine der folgenden Antworten:
Bitte wählen Sie nur eine der folgenden Antworten aus:

- ◯ Das Kind macht physische und psychische Fortschritte die seinem Alter entsprechen (lt. Arzt/Ärztinnen, Pädagog:innen etc.)
- ◯ Das Kind macht physische und psychische Fortschritte, die ich als angemessen empfinde
- ◯ Das Kind neigt nicht zu Infekten und hat ein starkes Immunsystem
- ◯ Das Kind ist meiner Beobachtung nach glücklich und ausgeglichen
- ◯ Das Kind macht überdurchschnittliche physische und psychische Fortschritte

VEGANE ERNÄHRUNG: DEFINITION UND BERATUNG

Wie ernähren Sie sich? *

Bitte wählen Sie eine der folgenden Antworten:
Bitte wählen Sie nur eine der folgenden Antworten aus:

- ◯ Vegan (rein pflanzlich)
- ◯ Vegetarisch (kein Fisch/Fleisch)
- ◯ Pescetarisch (kein Fleisch, aber Fisch und/oder Meeresfrüchte)
- ◯ Omnivor (Mischkost, auch Fisch, Meeresfrüchte und Fleisch)

Haben Sie in Ihrem beruflichen Alltag mit Veganer:innen zu tun? *

Bitte wählen Sie eine der folgenden Antworten:
Bitte wählen Sie nur eine der folgenden Antworten aus:

- ◯ Ja
- ◯ Nein
- ◯ Weiß nicht

Bieten Sie Beratung zu Nahrungsergänzungsmittel in der veganen Ernährung an? *

Bitte wählen Sie eine der folgenden Antworten:
Bitte wählen Sie nur eine der folgenden Antworten aus:

- ◯ Ja
- ◯ Nein

Ist vegane Ernährung gesund? *

Bitte wählen Sie eine der folgenden Antworten:
Bitte wählen Sie nur eine der folgenden Antworten aus:

- ◯ Ja, immer
- ◯ Ja, aber nur wenn Veganer:innen umfassend über eine ausgewogene, rein pflanzliche Ernährung informiert sind
- ◯ Nein
- ◯ Weiß nicht

Warum ernähren Sie sich vegan? (Mehrfachauswahl möglich) *

Beantworten Sie diese Frage nur, wenn folgende Bedingungen erfüllt sind:
Antwort war 'Vegan (rein pflanzlich)' bei Frage ' [G5Q00001]' (Wie ernähren Sie sich?)

Wählen Sie alle zutreffenden Optionen
Bitte wählen Sie alle zutreffenden Antworten aus:

- ☐ Aus gesundheitlichen Gründen
- ☐ Aus Gründen des Tierschutzes
- ☐ Aus Gründen des Klimaschutzes
- ☐ Weil mir tierische Lebensmittel nicht schmecken
- ☐ Sonstiges

Wie lange ernähren Sie sich schon vegan? *

Beantworten Sie diese Frage nur, wenn folgende Bedingungen erfüllt sind:
Antwort war 'Vegan (rein pflanzlich)' bei Frage ' [G5Q00001]' (Wie ernähren Sie sich?)

Bitte wählen Sie eine der folgenden Antworten:
Bitte wählen Sie nur eine der folgenden Antworten aus:

- ◯ Weniger als einen Monat
- ◯ Ca. 3 Monate
- ◯ Ca. 6 Monate
- ◯ Ca. 1 Jahr
- ◯ Ca. 1-3 Jahre
- ◯ Ca. 3-5 Jahre
- ◯ Ca. 5-10 Jahre
- ◯ Über 10 Jahre

VEGANE ERNÄHRUNG: DEFINITION UND BERATUNG 2

Bitte bewerten Sie folgende Aussagen: *

Bitte wählen Sie die zutreffende Antwort für jeden Punkt aus:

	trifft zu	trifft eher zu	trifft eher nicht zu	trifft nicht zu
**Ich rate allen Veganer:innen davon ab, sich vegan zu ernähren**	○	○	○	○
**Ich rate Veganer:innen während ihrer Schwangerschaft von ihrer Ernährungsweise ab**	○	○	○	○
**Ich rate davon ab, Säuglinge und Kinder vegan zu ernähren**	○	○	○	○

VEGANE ERNÄHRUNG: DEFINITION UND BERATUNG 3

Bitte bewerten Sie folgende Aussagen: *

Bitte wählen Sie die zutreffende Antwort für jeden Punkt aus:

	oft	selten	nie	weiß nicht
**Ich berate in meinem Berufsalltag Veganer:innen**	◯	◯	◯	◯
**Ich berate in meinem Berufsalltag schwangere Veganerinnen**	◯	◯	◯	◯
**Ich berate in meinem Berufsalltag Eltern veganer Kinder**	◯	◯	◯	◯

NAHRUNGSERGÄNZUNGSMITTEL: DEFINITION UND VERSTÄNDNIS

Wie wirken Nahrungsergänzungsmittel? *

Bitte wählen Sie eine der folgenden Antworten:
Bitte wählen Sie nur eine der folgenden Antworten aus:

- ◯ Wie rezeptpflichtige Medikamente
- ◯ Wie frei verkäufliche Medikamente
- ◯ Wie Lebensmittel
- ◯ Wie Placebos
- ◯ Sind in ihrer Wirkung mit keiner der Antwortmöglichkeiten vergleichbar
- ◯ Weiß nicht

NAHRUNGSERGÄNZUNGSMITTEL: NUTZEN, RISIKEN UND ERWARTUNGSHALTUNG

Wie wirken sich ärztlich/pharmazeutisch/diätologisch empfohlene und richtig dosierte Nahrungsergänzungsmittel auf die Gesundheit aus?

*

Bitte wählen Sie eine der folgenden Antworten:

Bitte wählen Sie nur eine der folgenden Antworten aus:

- ◯ Sind immer vorteilhaft für die Gesundheit
- ◯ Können sich positiv auf die Gesundheit auswirken
- ◯ Haben keinen Einfluss auf die Gesundheit
- ◯ Können der Gesundheit schaden
- ◯ Sind immer gesundheitsgefährdend
- ◯ Weiß nicht

NAHRUNGSERGÄNZUNGSMITTEL: NUTZEN, RISIKEN UND ERWARTUNGSHALTUNG 2

Bitte bewerten Sie folgende Aussagen: *

Bitte wählen Sie die zutreffende Antwort für jeden Punkt aus:

	trifft zu	trifft eher zu	trifft eher nicht zu	trifft nicht zu	weiß nicht
**Die Einnahme von Nahrungsergänzungsmitteln während Zeiten körperlicher Belastung (wie Schwangerschaft, Entwicklungsunterstützung von Kindern, Krankheit, Stress etc.) ist gesundheitsfördernd**	○	○	○	○	○
**Die Einnahme von Nahrungsergänzungsmitteln ist vor allem für Veganer:innen vorteilhaft für die Gesundheit**	○	○	○	○	○
**Nahrungsergänzungsmittel haben eine negative Wirkung auf die Gesundheit von Mutter und Kind in der Schwangerschaft**	○	○	○	○	○
**Schwangere Veganerinnen sollten eher Nahrungsergänzungsmittel einnehmen als sich nicht vegan ernährende Schwangere**	○	○	○	○	○
**Vegan ernährte Kinder sollten keine Nahrungsergänzungsmittel**	○	○	○	○	○

	trifft zu	trifft eher zu	trifft eher nicht zu	trifft nicht zu	weiß nicht
einnehmen					
Vegan ernährte Kinder sollten eher Nahrungsergänzungsmittel einnehmen als Kinder, die durch Mischkost ernährt werden	○	○	○	○	○

NAHRUNGSERGÄNZUNGSMITTEL: NUTZEN, RISIKEN UND ERWARTUNGSHALTUNG 3

Bitte beantworten Sie nachfolgende Fragen durch Markierung der zutreffenden Antwort: *

Bitte wählen Sie die zutreffende Antwort für jeden Punkt aus:

	ja	eher ja	eher nein	nein	weiß nicht
**Ist eine vegane Ernährung in der Schwangerschaft gesundheitlich risikoreich für Mutter und Kind?**	○	○	○	○	○
**Helfen Nahrungsergänzungsmittel die Risiken einer vegan gelebten Schwangerschaft zu reduzieren?**	○	○	○	○	○
**Ist eine vegane Ernährung von Säuglingen und Kindern gesundheitlich risikoreich?**	○	○	○	○	○
**Helfen Nahrungsergänzungsmittel die Risiken eines Nährstoffmangels durch die vegane Ernährung von Säuglingen und Kindern zu reduzieren?**	○	○	○	○	○
**Ist es während der Schwangerschaft gesundheitlich notwendig Nahrungsergänzungsmittel zu supplementieren?**	○	○	○	○	○

NAHRUNGSERGÄNZUNGSMITTEL: EMPFEHLUNG

Bitte beantworten Sie nachfolgende Fragen mit ja oder nein: *

Bitte wählen Sie die zutreffende Antwort für jeden Punkt aus:

	Ja	Nein
**Nehmen Sie selbst Nahrungsergänzungsmittel ein?**	○	○
**Befürworten Sie die Einnahme von Nahrungsergänzungsmitteln in der Schwangerschaft (unabhängig von der Ernährungsweise)?**	○	○
**Befürworten Sie die Einnahme von Nahrungsergänzungsmitteln für Kinder, im Alter von 0-5 Jahren (unabhängig von der Ernährungsweise)?**	○	○
**Empfehlen Sie die Einnahme von Nahrungsergänzungsmitteln in der Schwangerschaft von Mischköstlerinnen?**	○	○
**Empfehlen Sie die Einnahme von Nahrungsergänzungsmitteln für Kinder, im Alter von 0-5 Jahren, die omnivor ernährt werden?**	○	○
**Empfehlen Sie die Einnahme von**	○	○

	Ja	Nein
**Nahrungsergänzungsmitteln in der Schwangerschaft von Veganerinnen?**		
**Empfehlen Sie die Einnahme von Nahrungsergänzungsmitteln, für Kinder im Alter von 0-5 Jahren, die vegan ernährt werden?**	○	○

Weshalb empfehlen Sie KEINE Einnahme von Nahrungsergänzungsmitteln, in der Schwangerschaft von Mischköstlerinnen? *

Beantworten Sie diese Frage nur, wenn folgende Bedingungen erfüllt sind:
Antwort war 'Nein' bei Frage ' [G12Q00001]' (Bitte beantworten Sie nachfolgende Fragen mit ja oder nein: (Empfehlen Sie die Einnahme von Nahrungsergänzungsmitteln in der Schwangerschaft von Mischköstlerinnen?))

Wählen Sie alle zutreffenden Optionen
Bitte wählen Sie maximal 3 Antworten.
Bitte wählen Sie alle zutreffenden Antworten aus:

- ☐ Nahrungsergänzungsmittel können vom schwangeren Körper schlecht aufgenommen werden
- ☐ Alle notwendigen Nährstoffe können auch über die Nahrung zugeführt werden
- ☐ Nahrungsergänzungsmittel schaden der Gesundheit
- ☐ Nahrungsergänzungsmittel haben keine nennenswerte Wirkung in der Schwangerschaft
- ☐ Die Möglichkeit einer falschen Dosierung ist in der Schwangerschaft zu risikoreich
- ☐ Sonstiges

Weshalb empfehlen Sie KEINE Einnahme von Nahrungsergänzungsmitteln, in der Schwangerschaft von Veganerinnen? *

Beantworten Sie diese Frage nur, wenn folgende Bedingungen erfüllt sind:
Antwort war 'Nein' bei Frage ' [G12Q00001]' (Bitte beantworten Sie nachfolgende Fragen mit ja oder nein: (Empfehlen Sie die Einnahme von Nahrungsergänzungsmitteln in der Schwangerschaft von Veganerinnen?))

Wählen Sie alle zutreffenden Optionen
Bitte wählen Sie maximal 3 Antworten.
Bitte wählen Sie alle zutreffenden Antworten aus:

- ☐ Nahrungsergänzungsmittel können vom schwangeren Körper schlecht aufgenommen werden
- ☐ Alle notwendigen Nährstoffe können auch über die vegane Nahrung zugeführt werden
- ☐ Nahrungsergänzungsmittel schaden der Gesundheit der Schwangeren
- ☐ Nahrungsergänzungsmittel haben keine nennenswerte Wirkung in der Schwangerschaft
- ☐ Die Möglichkeit einer falschen Dosierung ist in der Schwangerschaft zu risikoreich
- ☐ Sonstiges

Weshalb empfehlen Sie KEINE Einnahme von Nahrungsergänzungsmitteln, von omnivor ernährten Kindern (im Alter von 0-5 Jahren)? *

Beantworten Sie diese Frage nur, wenn folgende Bedingungen erfüllt sind:
Antwort war 'Nein' bei Frage ' [G12Q00001]' (Bitte beantworten Sie nachfolgende Fragen mit ja oder nein: (Empfehlen Sie die Einnahme von Nahrungsergänzungsmitteln für Kinder, im Alter von 0-5 Jahren, die omnivor ernährt werden?))

Wählen Sie alle zutreffenden Optionen
Bitte wählen Sie maximal 3 Antworten.
Bitte wählen Sie alle zutreffenden Antworten aus:

- ☐ Nahrungsergänzungsmittel können vom kindlichen Körper schlecht aufgenommen werden
- ☐ Alle notwendigen Nährstoffe können auch durch die Nahrung zugeführt werden
- ☐ Nahrungsergänzungsmittel schaden der Gesundheit
- ☐ Nahrungsergänzungsmittel haben keine nennenswerte Wirkung bei Kindern
- ☐ Die Möglichkeit einer falschen Dosierung, für Kinder im Alter von 0-5 Jahren, ist zu risikoreich
- ☐ Sonstiges

Weshalb empfehlen Sie KEINE Einnahme von Nahrungsergänzungsmitteln, von vegan ernährten Kindern (im Alter von 0-5 Jahren)?

*

Beantworten Sie diese Frage nur, wenn folgende Bedingungen erfüllt sind:
Antwort war 'Nein' bei Frage ' [G12Q00001]' (Bitte beantworten Sie nachfolgende Fragen mit ja oder nein: (Empfehlen Sie die Einnahme von Nahrungsergänzungsmitteln, für Kinder im Alter von 0-5 Jahren, die vegan ernährt werden?))

Wählen Sie alle zutreffenden Optionen
Bitte wählen Sie maximal 3 Antworten.
Bitte wählen Sie alle zutreffenden Antworten aus:

- ☐ Nahrungsergänzungsmittel können vom kindlichen Körper schlecht aufgenommen werden
- ☐ Alle notwendigen Nährstoffe können auch durch die vegane Nahrung zugeführt werden
- ☐ Nahrungsergänzungsmittel schaden der Gesundheit
- ☐ Nahrungsergänzungsmittel haben keine nennenswerte Wirkung bei Kindern
- ☐ Die Möglichkeit einer falschen Dosierung, für Kinder im Alter von 0-5 Jahren, ist zu risikoreich
- ☐ Sonstiges

Weshalb empfehlen Sie die Einnahme von Nahrungsergänzungsmitteln, in der Schwangerschaft von Mischköstlerinnen? *

Beantworten Sie diese Frage nur, wenn folgende Bedingungen erfüllt sind:
Antwort war 'Ja' bei Frage ' [G12Q00001]' (Bitte beantworten Sie nachfolgende Fragen mit ja oder nein: (Empfehlen Sie die Einnahme von Nahrungsergänzungsmitteln in der Schwangerschaft von Mischköstlerinnen?))

Bitte wählen Sie eine der folgenden Antworten:
Bitte wählen Sie nur eine der folgenden Antworten aus:

- ◯ Nahrungsergänzungsmittel unterstützen die positive Entwicklung des ungeborenen Kindes
- ◯ Nahrungsergänzungsmittel unterstützen die Gesundheit der Schwangeren
- ◯ Nahrungsergänzungsmittel unterstützen die positive Entwicklung des ungeborenen Kindes und die Gesundheit der Schwangeren
- ◯ Notwendige Nährstoffe, die durch die omnivore Ernährung nicht ausreichend zugeführt werden, sollten während der Schwangerschaft risikominimierend ergänzt werden
- ◯ Sonstiges

Weshalb empfehlen Sie die Einnahme von Nahrungsergänzungsmitteln in der Schwangerschaft von Veganerinnen? *

Beantworten Sie diese Frage nur, wenn folgende Bedingungen erfüllt sind:
Antwort war 'Ja' bei Frage ' [G12Q00001]' (Bitte beantworten Sie nachfolgende Fragen mit ja oder nein: (Empfehlen Sie die Einnahme von Nahrungsergänzungsmitteln in der Schwangerschaft von Veganerinnen?))

Bitte wählen Sie eine der folgenden Antworten:
Bitte wählen Sie nur eine der folgenden Antworten aus:

- ◯ Nahrungsergänzungsmittel unterstützen die positive Entwicklung des ungeborenen Kindes
- ◯ Nahrungsergänzungsmittel unterstützen die Gesundheit der veganen Schwangeren
- ◯ Nahrungsergänzungsmittel unterstützen die positive Entwicklung des ungeborenen Kindes und die Gesundheit der veganen Schwangeren
- ◯ Notwendige Nährstoffe, die durch die vegane Ernährung nicht ausreichend zugeführt werden, sollten während der Schwangerschaft risikominimierend ergänzt werden
- ◯ Sonstiges

Weshalb empfehlen Sie die Einnahme von Nahrungsergänzungsmitteln, von omnivor ernährten Kindern (im Alter von 0-5 Jahren)? *

Beantworten Sie diese Frage nur, wenn folgende Bedingungen erfüllt sind:
Antwort war 'Ja' bei Frage ' [G12Q00001]' (Bitte beantworten Sie nachfolgende Fragen mit ja oder nein: (Empfehlen Sie die Einnahme von Nahrungsergänzungsmitteln für Kinder, im Alter von 0-5 Jahren, die omnivor ernährt werden?))

Bitte wählen Sie eine der folgenden Antworten:
Bitte wählen Sie nur eine der folgenden Antworten aus:

- ◯ Nahrungsergänzungsmittel unterstützen die positive Entwicklung des Kindes
- ◯ Notwendige Nährstoffe, die durch die Ernährung nicht ausreichend zugeführt werden, können ergänzt werden
- ◯ Sonstiges

Weshalb empfehlen Sie die Einnahme von Nahrungsergänzungsmitteln, von vegan ernährten Kindern (im Alter von 0-5 Jahren)? *

Beantworten Sie diese Frage nur, wenn folgende Bedingungen erfüllt sind:
Antwort war 'Ja' bei Frage ' [G12Q00001]' (Bitte beantworten Sie nachfolgende Fragen mit ja oder nein: (Empfehlen Sie die Einnahme von Nahrungsergänzungsmitteln, für Kinder im Alter von 0-5 Jahren, die vegan ernährt werden?))

Bitte wählen Sie eine der folgenden Antworten:
Bitte wählen Sie nur eine der folgenden Antworten aus:

- ◯ Nahrungsergänzungsmittel unterstützen die positive Entwicklung des Kindes
- ◯ Notwendige Nährstoffe, die durch die vegane Ernährung nicht ausreichend zugeführt werden, können ergänzt werden
- ◯ Sonstiges

NAHRUNGSERGÄNZUNGSMITTEL: EMPFEHLUNG 2

Bitte bewerten Sie folgende Aussagen: *

Bitte wählen Sie die zutreffende Antwort für jeden Punkt aus:

	trifft zu	trifft eher zu	trifft eher nicht zu	trifft nicht zu
**Ich würde einer Veganerin eher zu Nahrungsergänzungsmitteln in der Schwangerschaft raten als einer schwangeren Mischköstlerin**	○	○	○	○
**Ich würde einer schwangeren Veganerin zusätzliche Nahrungsergänzungsmittel empfehlen, zu denen ich einer Mischköstlerin nicht raten würde**	○	○	○	○
**Ich würde Nahrungsergänzungsmittel für ein vegan ernährtes Kind empfehlen, ohne dass zuvor ein Nährstoffmangel diagnostiziert wurde (via Blutbefund)**	○	○	○	○
**Ich würde Nahrungsergänzungsmittel für Kinder nicht grundsätzlich empfehlen - nur wenn es wegen weiterer Indikatoren (z.B. Blutbefund) notwendig ist**	○	○	○	○

NAHRUNGSERGÄNZUNGSMITTEL: EMPFEHLUNG 3

Welche Nahrungsergänzungsmittel würden Sie einer schwangeren Veganerin empfehlen? (Mehrfachauswahl möglich) *

Wählen Sie alle zutreffenden Optionen

Bitte wählen Sie alle zutreffenden Antworten aus:

- ☐ Vitamin B12
- ☐ Folsäure
- ☐ Omega-3-Fettsäuren (DHA/EPA)
- ☐ Eisen
- ☐ Vitamin D
- ☐ Iod
- ☐ Zink
- ☐ Kalzium
- ☐ Magnesium
- ☐ Kombinationspräparate (z.B. Multivitaminpräparate für Schwangere)
- ☐ Keine
- ☐ Andere

Welche Nahrungsergänzungsmittel würden Sie einer schwangeren Mischköstlerin empfehlen? (Mehrfachauswahl möglich) *

Wählen Sie alle zutreffenden Optionen

Bitte wählen Sie alle zutreffenden Antworten aus:

- [ ] Vitamin B12
- [ ] Folsäure
- [ ] Omega-3-Fettsäuren (DHA/EPA)
- [ ] Eisen
- [ ] Vitamin D
- [ ] Iod
- [ ] Zink
- [ ] Kalzium
- [ ] Magnesium
- [ ] Kombinationspräparate (z.B. Multivitaminpräparate für Schwangere)
- [ ] Keine
- [ ] Andere

Welche Nahrungsergänzungsmittel würden Sie einem vegan ernährten Kind, im Alter von 0-5 Jahren, empfehlen? (Mehrfachauswahl möglich) *

Wählen Sie alle zutreffenden Optionen

Bitte wählen Sie alle zutreffenden Antworten aus:

- ☐ Vitamin B12
- ☐ Folsäure
- ☐ Omega-3-Fettsäuren (DHA/EPA)
- ☐ Eisen
- ☐ Vitamin D
- ☐ Iod
- ☐ Zink
- ☐ Kalzium
- ☐ Magnesium
- ☐ Kombinationspräparate (z.B. Multivitaminpräparate für Kinder)
- ☐ Keine
- ☐ Andere

Welche Nahrungsergänzungsmittel würden Sie einem Kind, im Alter von 0-5 Jahren empfehlen, das omnivor ernährt wird? (Mehrfachauswahl möglich) *

Wählen Sie alle zutreffenden Optionen

Bitte wählen Sie alle zutreffenden Antworten aus:

- ☐ Vitamin B12
- ☐ Folsäure
- ☐ Omega-3-Fettsäuren (DHA/EPA)
- ☐ Eisen
- ☐ Vitamin D
- ☐ Iod
- ☐ Zink
- ☐ Kalzium
- ☐ Magnesium
- ☐ Kombinationspräparate (z.B. Multivitaminpräparate für Kinder)
- ☐ Keine
- ☐ Andere

NAHRUNGSERGÄNZUNGSMITTEL: WISSENSSTAND

Wissen Sie, dass es vegane Alternativen zu nicht-veganen Nahrungsergänzungsmitteln gibt (z.B. vegane Vitamin D3- und vegane Omega-3-Fettsäurepräparate)? *

Bitte wählen Sie eine der folgenden Antworten:

Bitte wählen Sie nur eine der folgenden Antworten aus:

- ○ Ja
- ○ Bis jetzt wusste ich noch nicht davon

NAHRUNGSERGÄNZUNGSMITTEL: WISSENSSTAND 2

Bitte bewerten Sie folgende Aussagen: *

Bitte wählen Sie die zutreffende Antwort für jeden Punkt aus:

	trifft zu	trifft eher zu	trifft eher nicht zu	trifft nicht zu	weiß nicht
**Mein persönlicher Kenntnisstand zu Nahrungsergänzungsmitteln ist umfassend**	○	○	○	○	○
**Mein persönlicher Kenntnisstand zu veganen Nahrungsergänzungsmitteln ist umfassend**	○	○	○	○	○
**Die Bioverfügbarkeit von Nahrungsergänzungsmitteln ist hoch**	○	○	○	○	○
**Die Bioverfügbarkeit veganer Nahrungsergänzungsmittel entspricht derer nicht veganer Nahrungsergänzungsmittel**	○	○	○	○	○

NAHRUNGSERGÄNZUNGSMITTEL: WISSENSSTAND 3

Bitte beantworten Sie folgende Fragen: *

Bitte wählen Sie die zutreffende Antwort für jeden Punkt aus:

	Ja	Nein	Weiß nicht
**Können schwangere Veganerinnen ihren Nährstoffbedarf durch eine rein pflanzliche Ernährung decken?**	○	○	○
**Sind mögliche Nährstoffmängel einer veganen Ernährung in der Schwangerschaft durch Nahrungsergänzungsmittel ausgleichbar?**	○	○	○
**Kann der Nährstoffbedarf vegan ernährter Kinder (im Alter von 0-5 Jahren) durch rein pflanzliche Ernährung gedeckt werden?**	○	○	○
**Sind mögliche Nährstoffmängel vegan ernährter Kinder (im Alter von 0-5 Jahren) durch Nahrungsergänzungsmittel ausgleichbar?**	○	○	○

NAHRUNGSERGÄNZUNGSMITTEL: INFORMATIONSQUELLEN

Wo wird man am besten zu Nahrungsergänzungsmitteln in der Schwangerschaft beraten? *

Bitte wählen Sie eine der folgenden Antworten:
Bitte wählen Sie nur eine der folgenden Antworten aus:

- ◯ Arzt/Ärztin für Allgemeinmedizin
- ◯ Gynäkolog:in
- ◯ Apotheker:in
- ◯ Diätolog:in
- ◯ Hebamme
- ◯ Fachliteratur und Fachzeitschriften
- ◯ Internet
- ◯ Eine Beratung ist nicht notwendig

Wo wird man am besten zu Nahrungsergänzungsmitteln im Kindesalter (0-5 Jahre) beraten? *

Bitte wählen Sie eine der folgenden Antworten:
Bitte wählen Sie nur eine der folgenden Antworten aus:

- ◯ Arzt/Ärztin für Allgemeinmedizin
- ◯ Pädiater:in
- ◯ Apotheker:in
- ◯ Diätolog:in
- ◯ Fachliteratur und Fachzeitschriften
- ◯ Internet
- ◯ Eine Beratung ist nicht notwendig

NAHRUNGSERGÄNZUNGSMITTEL:

EXPERT:INNENEINFLUSS

Beraten Sie schwangere Veganerinnen zur Einnahme von Nahrungsergänzungsmitteln? *

Bitte wählen Sie eine der folgenden Antworten:

Bitte wählen Sie nur eine der folgenden Antworten aus:

- ○ Ja
- ○ Nein

Beraten Sie Eltern vegan ernährter Kinder, zur Verabreichung von Nahrungsergänzungsmitteln an ihre Kinder? *

Bitte wählen Sie eine der folgenden Antworten:

Bitte wählen Sie nur eine der folgenden Antworten aus:

- ○ Ja
- ○ Nein

Teilen Ihnen Ihre Kund:innen/Patient:innen mit, dass sie Veganer:innen sind? *

Bitte wählen Sie eine der folgenden Antworten:

Bitte wählen Sie nur eine der folgenden Antworten aus:

- ○ Ja
- ○ Nein
- ○ Selten

Kommt es vor, dass vegane Kund:innen/Patient:innen (Schwangere oder Eltern) Ihnen erst zu einem späteren Zeitpunkt (nicht beim Erstgespräch/Erstkontakt) von ihrer veganen Ernährung berichten? *

Bitte wählen Sie eine der folgenden Antworten:
Bitte wählen Sie nur eine der folgenden Antworten aus:

- ◯ Ja
- ◯ Nein

Ihrer Einschätzung zufolge: Was veranlasst Kund:innen/Patient:innen, ihre vegane Ernährung gegenüber medizinischen Expert:innen unerwähnt zu lassen? (Mehrfachauswahl möglich) *

Wählen Sie alle zutreffenden Optionen
Bitte wählen Sie alle zutreffenden Antworten aus:

- ☐ Das Thema der veganen Ernährung wird als nicht erwähnenswert eingeschätzt
- ☐ Kund:innen/Patient:innen sind davon überzeugt, bereits alle nötigen Informationen zu haben
- ☐ Kund:innen/Patient:innen befürchten, für ihre Ernährungsweise kritisiert zu werden
- ☐ Kund:innen/Patient:innen haben es ohne spezifische Gründe nicht erwähnt (z.B. vergessen)
- ☐ Kund:innen/Patient:innen sind skeptisch gegenüber Expert:innen
- ☐ Weiß nicht

NAHRUNGSERGÄNZUNGSMITTEL: APPLIKATION UND COMPLIANCE

Welche Verabreichungsform von Nahrungsergänzungsmitteln ist am ehesten für Kinder geeignet? *

Wählen Sie alle zutreffenden Optionen

Bitte wählen Sie alle zutreffenden Antworten aus:

- ☐ Tropfen
- ☐ Saft/Sirup
- ☐ Tablette ganz
- ☐ Tablette zu Pulver zerstoßen (und in Wasser, Fruchtsaft, Joghurt etc. gelöst)
- ☐ Kapsel ganz
- ☐ Kapselinhalt geleert (und in Wasser, Fruchtsaft, Joghurt etc. gelöst)
- ☐ Nahrungsergänzungsmittel in Form einer "Süßigkeit" (z.B. Gummidrops, Toffees)
- ☐ Zahnpaste/Zahncreme mit Nährstoffen angereichert (z.B. mit Vitamin B12)
- ☐ Mundspray zur sublingualen Anwendung (z.B. mit Vitamin D, Vitamin B12)
- ☐ Keine

Was ist Ihr größtes Bedenken, wenn schwangere Veganerinnen, Nahrungsergänzungsmittel einnehmen? *

Bitte wählen Sie eine der folgenden Antworten:

Bitte wählen Sie nur eine der folgenden Antworten aus:

- ○ Dem ungeborenen Kind wird durch falsche Dosierung geschadet
- ○ Mutter und Kind werden in der Schwangerschaft durch die Zufuhr von Nahrungsergänzungsmitteln gesundheitlich belastet
- ○ Der schwangere Körper wird durch die Einnahme von Nahrungsergänzungsmitteln mehr belastet als unterstützt
- ○ Die Gesundheit des ungeborenen Kindes wird durch die Einnahme von Nahrungsergänzungsmitteln belastet (auch bei richtiger Dosierung)
- ○ Ich habe keine Bedenken, da Nahrungsergänzungsmittel sicher sind
- ○ Sonstiges

Was ist Ihr größtes Bedenken, wenn schwangere Veganerinnen keine Nahrungsergänzungsmittel einnehmen? *

Bitte wählen Sie eine der folgenden Antworten:
Bitte wählen Sie nur eine der folgenden Antworten aus:

- ◯ Komplikationen während Schwangerschaft und/oder Geburt durch Nährstoffunterversorgung
- ◯ Das Risiko einer Frühgeburt wird erhöht
- ◯ Das Risiko einer Fehlgeburt wird erhöht
- ◯ Gesundheitliche Konsequenzen für die Mutter und/oder das Neugeborene durch Nährstoffmängel
- ◯ Ich habe keine Bedenken
- ◯ Sonstiges

Was ist Ihr größtes Bedenken, wenn Eltern ihren vegan ernährten Kindern (im Alter von 0-5 Jahren) Nahrungsergänzungsmittel verabreichen? *

Bitte wählen Sie eine der folgenden Antworten:
Bitte wählen Sie nur eine der folgenden Antworten aus:

- ◯ Dem Kind wird durch falsche Dosierung geschadet
- ◯ Das Kind wird durch die Zufuhr von Nahrungsergänzungsmitteln gesundheitlich belastet (auch bei richtiger Dosierung)
- ◯ Ich habe keine Bedenken, da Nahrungsergänzungsmittel sicher sind
- ◯ Sonstiges

Was ist Ihr größtes Bedenken, wenn Eltern ihren vegan ernährten Kindern (im Alter von 0-5 Jahren) keine Nahrungsergänzungsmittel verabreichen? *

Bitte wählen Sie eine der folgenden Antworten:

Bitte wählen Sie nur eine der folgenden Antworten aus:

- ◯ Gesundheitliche Konsequenzen durch Nährstoffmängel
- ◯ Verzögerte körperliche und/oder geistige Entwicklung durch Nährstoffunterversorgung
- ◯ Ich habe keine Bedenken
- ◯ Sonstiges

NAHRUNGSERGÄNZUNGSMITTEL: APPLIKATION UND COMPLIANCE 2

Bitte beantworten Sie nachfolgenden Fragen durch Markierung der zutreffenden Antwort: *

Bitte wählen Sie die zutreffende Antwort für jeden Punkt aus:

	hoch	eher hoch	eher niedrig	niedrig	weiß nicht
**Wie beurteilen Sie die Compliance Ihrer Kundinnen/ Patientinnen, betreffend der Einnahme von Nahrungsergänzungsmitteln während der Schwangerschaft?**	○	○	○	○	○
**Wie beurteilen Sie die Compliance Ihrer VEGANEN Kundinnen/ Patientinnen, betreffend der Einnahme von Nahrungsergänzungsmitteln während der Schwangerschaft?**	○	○	○	○	○

NAHRUNGSERGÄNZUNGSMITTEL: APPLIKATION UND COMPLIANCE 3

Bitte beantworten Sie nachfolgenden Fragen durch Markierung der zutreffenden Antwort: *

Bitte wählen Sie die zutreffende Antwort für jeden Punkt aus:

	sehr	eher	eher nicht	nicht	weiß nicht
**Wie konsequent beurteilen Sie Ihre veganen Kund:innen/ Patient:innen, bei der empfohlenen Verabreichung von Nahrungsergänzungsmitteln an deren Kinder?**	○	○	○	○	○
**Wie schwierig beurteilen Sie eine dosisgenaue Verabreichung von Nahrungsergänzungsmitteln (über Tropfen, Tabletten, Kapseln, Säfte etc.), von veganen Eltern an deren Kinder?**	○	○	○	○	○

NAHRUNGSERGÄNZUNGSMITTEL: APPLIKATION UND COMPLIANCE 3

Bitte beantworten Sie nachfolgenden Fragen durch Markierung der zutreffenden Antwort: *

Bitte wählen Sie die zutreffende Antwort für jeden Punkt aus:

	ja	nein	weiß nicht
**Ist die regelmäßige Einnahme von Nahrungsergänzungsmitteln (die durch medizinische Expert:innen empfohlen werden) in der Schwangerschaft wichtig für deren Wirksamkeit?**	○	○	○
**Ist die regelmäßige Einnahme von Nahrungsergänzungsmitteln (die durch medizinische Expert:innen empfohlen werden) im Kindesalter (von 0-5 Jahren) wichtig für deren Wirksamkeit?**	○	○	○

NAHRUNGSERGÄNZUNGSMITTEL: APPLIKATION UND COMPLIANCE 4

Wie beurteilen Sie die Verabreichung von Nahrungsergänzungsmitteln an Kinder (im Alter von 0-5 Jahren)? *

Bitte wählen Sie die zutreffende Antwort für jeden Punkt aus:

	unkompliziert	eher unkompliziert	eher kompliziert	kompliziert	weiß nicht
**Tropfen**	○	○	○	○	○
**Saft/Sirup**	○	○	○	○	○
**Tablette ganz**	○	○	○	○	○
**Tablette zu Pulver zerstoßen (und in Wasser, Fruchtsaft, Joghurt etc. gelöst)**	○	○	○	○	○
**Kapsel ganz**	○	○	○	○	○
**Kapselinhalt geleert (und in Wasser, Fruchtsaft, Joghurt etc. gelöst)**	○	○	○	○	○
**Nahrungsergänzungsmittel in Form einer "Süßigkeit" (z.B. Gummidrops, Toffees)**	○	○	○	○	○
**Zahnpaste/Zahncreme mit Nährstoffen angereichert (z.B. mit Vitamin B12)**	○	○	○	○	○
**Mundspray zur sublingualen Anwendung (z.B. mit Vitamin D, Vitamin B12)**	○	○	○	○	○

Vielen Dank für Ihre Umfrageteilnahme! Bei Fragen steht Ihnen Wolfgang Huber-Schneider (Studiendurchführung) unter folgendem Kontakt zur Verfügung:

a00225229@unet.univie.ac.at

Senden Sie Ihre Umfage ein.
Vielen Dank für die Beantwortung des Fragebogens.
