# Supplementary material for: Can Dietary Supplements Be Linked to a Vegan Diet and Health Risk Modulation During Vegan Pregnancy, Infancy, and Early Childhood? The VedieS Study Protocol for an Explorative, Quantitative, Cross-Sectional Study
Source: Int J Environ Res Public Health. 2025 Jul 31;22(8):1210. doi: 10.3390/ijerph22081210 (PMC12386434; doi:10.3390/ijerph22081210)
Supplement: Supplementary file 1 [file ijerph-22-01210-s001.zip › S3_3677589.pdf]

| **Beschluss der Ethikkommission**<br>Decision of the Ethics Committee | universität wien<br>Ethikkommission |
| --- | --- |

Antragsteller*in / Applicant: **Wolfgang Huber-Schneider**
Bearbeitungsnummer / Reference Number: **01021**
Projekttitel / Title of Project: **Can dietary supplements be linked to a vegan diet and health risk modulation during vegan pregnancy, infancy and early childhood?**

Die Stellungnahme der Ethikkommission erfolgt aufgrund folgender eingereichter Unterlagen / The decision of the Ethics Committee is based on the following documents:

22.05.2023

• application form 22052023 • ArztausweisDanielKönig • Aushang Gynäkologinnen • Aushang Pädiaterinnen • Aushang Pharmazeutinnen Diätologinnen • Aushang Veganerinnen • Facharzt_Koenig_2022 • Fragebogen Expertinnen 22052023 • Fragebogen Veganerinnen 22052023 • Kurzinformation 22052023 • study protocol 22052023 • Teilnehmerinnneninformation 22052023

31.07.2023

• Begleitschreiben - cover letter • Fragebogen Expertinnen korrigierte Version • Fragebogen Expertinnen mit track changes • Fragebogen Veganerinnen korrigierte Version • Fragebogen Veganerinnen mit track changes • Teilnehmerinneninformation und Einwilligungserklärung korrigierte Version • Teilnehmerinneninformation und Einwilligungserklärung mit track changes

Die Kommission fasst folgenden Beschluss (mit X markiert) / The Ethics Committee has made the following decision (marked with an X):

☒ Zustimmung: Es besteht kein ethischer Einwand gegen die Durchführung der Studien. / Consent: There is no ethical objection to conduct the study as proposed.

☐ Negative Beurteilung: Der Antrag wird von der Ethikkommission abgelehnt. / Negative evaluation: The proposal is rejected by the Ethics Committee.

Inhaltliche Abänderungen müssen der Ethikkommission vorgelegt werden. / Amendments to the content must be presented to the Ethics Committee.

Unterschrift / Signature — Datum / Date

Vor [signature] — ETHIKKOMMISSION UNIVERSITÄT WIEN — 01.09.2023

Vorsitzender der Ethikkommission / Chair of the Ethics Committee
Univ.-Prof. MMag. DDDr. Martin Voracek
