# Supplementary material for: Can Dietary Supplements Be Linked to a Vegan Diet and Health Risk Modulation During Vegan Pregnancy, Infancy, and Early Childhood? The VedieS Study Protocol for an Explorative, Quantitative, Cross-Sectional Study
Source: Int J Environ Res Public Health. 2025 Jul 31;22(8):1210. doi: 10.3390/ijerph22081210 (PMC12386434; doi:10.3390/ijerph22081210)
Supplement: Supplementary file 1 [file ijerph-22-01210-s001.zip › S4_3677589.pdf]

Note to the reviewers:

Some questions in this survey are conditional, meaning they are only displayed if a previous answer logically leads to them. In both the German and English versions of the questionnaire, these conditions are indicated in *italic text* directly below the relevant question. Survey participants will not see or need to read these notes - they are automatically guided through the questionnaire based on their prior responses. Additionally, the section headings for the different test categories (highlighted in blue) are not visible to participants.

Please also note that the attached versions of the questionnaire are intended for illustrative purposes only. In the actual online survey, the structure is more intuitive and user-friendly, with dynamic filtering that ensures clarity and logic. This level of interactivity and visual clarity cannot be fully replicated in the static PDF format.

Survey of Vegans on a Vegan Diet and the Use of Dietary Supplements During Pregnancy, Infancy, and Early Childhood

Participant Information and Informed Consent for Study Participation

Working Title of the Study:

Can dietary supplements be linked to vegan diet and health risk modulation during pregnancy, infancy, and early childhood?

Dear Participant,

We invite you to participate in the study mentioned above.

Your participation in this study is voluntary. You may withdraw from the survey at any time without providing any reason, and your willingness to participate will be considered revoked. Refusal to participate or early withdrawal from the study will not have any negative consequences for you. Studies of this nature are necessary to gain reliable new scientific insights. However, a prerequisite for conducting such studies is that you give your consent to participate. NO sensitive data such as your name, email address, or IP address will be collected. Completing the survey will take approximately 25 minutes of your time. Please read the following text carefully and do not hesitate to ask questions. If you have any questions, please contact the doctoral student conducting the study, Wolfgang Huber-Schneider (contact details below).

Please confirm your consent (confirmation = click on "Next" – starting the survey is considered confirmation of your consent):

• if you fully understand the nature and procedure of the study,
• if you are willing to participate, and
• if you understand your rights as a participant in this study.

What is the purpose of the study?

The aim of this study is to examine the relationship between counseling by medical professionals (e.g., physicians, pharmacists) and other influencing factors (e.g., social environment, social media) with a vegan diet and the intake of dietary supplements during pregnancy, infancy, and early childhood (up to the age of 5 years) among vegans. This will help identify and minimize potential health risks for vegans. Your participation will

contribute to improving information access and health risk prevention for vegans. This survey is part of a PhD project.

How does the study proceed?

The questionnaire is addressed to vegans. It includes questions about a vegan diet and the use of dietary supplements during pregnancy, infancy, and early childhood (up to 5 years of age). Completing the questionnaire will take approximately 25 minutes.

What are the benefits of participating in the study?

You are not expected to receive any direct personal benefits from participating. However, by answering the questionnaire, you are contributing to the identification and prevention of potential health risks for pregnant vegans and vegan-fed children. From a scientific perspective, the study aims to optimize the quality and accessibility of information for vegans by investigating the sources of information and their influence on diet and supplement intake.

Are there any risks or side effects associated with participating?

No discomfort or risks are expected from participating in this study.

Inclusion Criteria for Study Participation:

1. Pregnant individuals (aged 18 or older) who follow(ed) a vegan diet during their pregnancy/pregnancies
2. Parents who feed/fed their child/children (up to age 5) a vegan diet
3. Mothers who follow a vegan diet and breastfeed their children
4. Confirmation of participant information and consent

Exclusion Criteria for Study Participation:

5. Participants (aged 18 or older) who did not follow a vegan diet during their pregnancy/pregnancies
6. Parents who do/did not feed their child/children (up to age 5) a vegan diet
7. No confirmation of participant information and consent

At least one inclusion criterion (1, 2, or 3) must be fulfilled (always including 4). If criterion 7 is fulfilled, participation is excluded. If only 5 OR 6 is fulfilled (but not 7), participation is still possible.

Does participation affect daily life or involve any obligations?

No, you may participate in the survey at any time and withdraw at any point without consequences or other effects.

What should be done if symptoms, side effects, or injuries occur?

As this is a questionnaire-based survey, no symptoms or adverse effects are expected.

When can the study be terminated prematurely?

You may withdraw your consent and discontinue participation at any time without giving reasons and without any disadvantages resulting from this decision.

How will the data collected in this study be used?

No personal data is required to participate in the survey. Your responses will be completely anonymous. The collected anonymous data will be stored solely for statistical purposes. No personal data (e.g., name, IP address, email address) will be collected or stored. It will not be possible to trace any data back to your identity.

Are there any costs for participants? Will there be reimbursement or compensation?

There are no costs associated with participating in this study. No reimbursement or compensation will be provided for participating in the survey/study.

Possibility to ask further questions

If you have further questions regarding the study, please contact Wolfgang Huber-Schneider. Questions related to your rights as a participant will also be answered. Once general results of the study are available, you can be informed upon request.

Contact – Study Team / Doctoral Student

The survey is part of the dissertation of Wolfgang Huber-Schneider at the Department of Nutritional Sciences, University of Vienna, in cooperation with AGES (Austrian Agency for Health and Food Safety). For questions, please contact:

Doctoral Student: Mag. pharm. Wolfgang Huber-Schneider
Email: a00225229@unet.univie.ac.at

Study Leadership:
- Univ.-Prof. Mag. Dr. Karl-Heinz Wagner (Department of Nutritional Sciences, University of Vienna)
- Univ.-Doz.in Mag.a Dr.in Ingrid Kiefer (AGES)
- Mag. pharm. Wolfgang Huber-Schneider (Doctoral Student)

By clicking “Next,” you automatically consent to participate in the survey.

This survey contains 75 questions.

General/Demographics

Gender *

Please select only one of the following answers:

• Female

• Male

• Diverse

Age (in years) *

Please select only one of the following answers:

• Under 18

• 18-20

• 21-30

• 31-40

• 41-50

• 51-60

• 61 or older

Highest Completed Education *

Please select only one of the following answers:

• Compulsory School

• Apprenticeship/Vocational Training

• High School Diploma (Matura/Abitur)

• College

• University of Applied Sciences

• University

• Other

Monthly Net Household Income (including all household members, subsidies, and benefits) - in EUR or CHF *

Please select only one of the following answers:

• Up to 1,500

• 1,501 - 2,000

• 2,001 - 2,500

• 2,501 - 3,000

• 3,001 - 3,500

• 3,501 - 4,000

• Over 4,001

• Prefer not to say

Household Size (including children) *

Please select only one of the following answers:

• 1

• 2

• 3

• 4 or more

In which country do you live? *

Please select only one of the following answers:

• Austria

• Germany

• Switzerland

• Italy

• Another EU country

• Other

Where do you live (postal code)? *

Please enter your answer here:

Pregnancy *

Answer only if the selected gender was 'Female' or 'Diverse'.

• I am currently pregnant

• I have been pregnant once

• I have been pregnant multiple times

• I am currently pregnant and have been pregnant before

• I am NOT pregnant and have NEVER been pregnant

Do you have children? *

Please select only one of the following answers:

• Yes

• No

How many children do you have? *

Answer only if the answer to 'Do you have children?' was 'Yes'.

• 1

• 2

• 3

• More than 3

Did you/do you breastfeed your children? *

Answer only if: Gender = Female or Diverse, and Pregnancies = Multiple or Currently pregnant with previous pregnancies, and Has children = Yes, and Number of children = 2 or 3.

• Yes

• No

• I did not breastfeed all my children

Did you/do you breastfeed your child? *

Answer only if: Gender = Female or Diverse, and Pregnancies = One, and Has children = Yes, and Number of children = 1.

• Yes

• No

Health

What does health mean to you? (Rank up to 3 answers) *

All answers must be different. Rank a maximum of 3 answers in order of preference (1 to 8).

• A state of complete mental, physical, and social well-being

• Freedom from illness and ailments

• Feeling good – even without diagnoses or test results

• Not suffering from any diagnosed illness

• Being able to live everyday life without limitations

• Being mentally and physically resilient

• Being happy and free of complaints

• Being exceptionally high-performing

What does health during pregnancy mainly mean to you? *

Please select only one of the following answers:

• A pregnancy without complications

• A pregnancy in which the child develops ideally through provision of all necessary nutrients

• A pregnancy in which the child develops in line with medical developmental expectations

• A pregnancy in which one feels well – even without medical confirmation of health status

• A pregnancy that proceeds as expected and on schedule

What does health in childhood mainly mean to you? *

Please select only one of the following answers:

• The child makes physical and mental progress appropriate for their age (according to physicians, educators, etc.)

• The child makes physical and mental progress I consider appropriate

• The child is not prone to infections and has a strong immune system

• The child appears happy and balanced to me

• The child makes above-average physical and mental progress

Vegan Diet: Definition and Motivation

How do you eat? (Multiple answers possible) *

Select all applicable options

• I ALWAYS follow a strictly plant-based (vegan) diet

• I ALMOST ALWAYS eat vegan, but I occasionally consume honey and/or other bee products (e.g., propolis, royal jelly)

• I ALMOST ALWAYS eat vegan, but I occasionally consume dairy products (e.g., milk, butter, yogurt)

• I ALMOST ALWAYS eat vegan, but I occasionally eat fish and/or seafood (e.g., shrimp, mussels)

• I ALMOST ALWAYS eat vegan, but I occasionally eat meat (e.g., chicken)

• I ALMOST ALWAYS eat vegan, but I occasionally eat fish and meat

How often do you make an exception to your vegan diet? *

Answer only if you selected any of the 'almost always vegan' options above.

• About once or twice a year

• About once or twice every six months

• About once or twice a month

• About once or twice a week

Is a vegan diet healthy? *

Please select only one of the following answers:

• Yes

• No

• Don't know

Why do you eat vegan? (Multiple answers possible) *

Select all applicable options

• For health reasons

• For animal welfare

• For climate protection

• Because I don't like the taste of animal products

• Other

When did you start eating vegan? *

Answer only if you are or have been pregnant.

• Before my pregnancy/pregnancies

• During my pregnancy/pregnancies

• Between two pregnancies (i.e., not during every pregnancy)

In which trimester of pregnancy did you start eating vegan? *

Answer only if you selected 'During my pregnancy/pregnancies' above.

• First trimester

• Second trimester

• Third trimester

Did you follow a strictly vegan diet throughout your (current/most recent) pregnancy?*

Answer only if you are or have been pregnant.

• I continued eating vegan

• I ate vegetarian

• I also ate fish

• I also ate fish and meat

Why did you change your vegan diet? (Multiple answers possible) *

Answer only if you ate vegetarian, fish, or meat during pregnancy.

• Fear of harming my child with a vegan diet

• Because my physician advised me to

• Because my pharmacist advised me to

• Because my dietitian advised me to

• Because my midwife advised me to

• Because friends, family, and/or colleagues advised me to

• Because other vegans advised me to

• Due to information from the internet or social media (Instagram, Facebook, etc.)

• Due to information from scientific literature and journals

• Due to information from magazines, newspapers, and/or lifestyle magazines

• Other

Why did you NOT change your vegan diet? (Multiple answers possible) *

Answer only if you continued eating vegan during pregnancy.

• All nutrients for mother and child were covered

• Because I took supplements to prevent nutrient deficiencies

• Because my physician advised me to continue

• Because my pharmacist advised me to continue

• Because my dietitian advised me to continue

• Because my midwife advised me to continue

• Because friends, family, and/or colleagues advised me to continue

• Because other vegans advised me to continue

• Due to information from the internet or social media (Instagram, Facebook, etc.)

• Due to information from scientific literature and journals

• Due to information from magazines, newspapers, and/or lifestyle magazines

• I did not consider changing my diet

• Other

How long have you been eating vegan? *

• Less than a month

• About 3 months

• About 6 months

• About 1 year

• About 1-3 years

• About 3-5 years

• About 5-10 years

• More than 10 years

Dietary Supplements: Definition and Understanding

Which dietary supplements do you know? Name up to three examples *

Please enter your answer here:

What are dietary supplements? (Multiple answers possible) *

• Foods that supplement the normal human diet

• Concentrates to be taken in small, measured amounts in dosage form

• Typical components of dietary supplements include vitamins, minerals, essential fatty acids, amino acids, and plant extracts

• Dietary supplements are medications

• Dietary supplements are foods like candy, sodas, teas, etc.

• Don't know

How do dietary supplements work? *

Please select only one of the following answers:

• Like prescription medications

• Like over-the-counter medications

• Like food

• Like placebos (inactive substances)

• Cannot be compared to any of the above

• Don't know

Dietary Supplements: Benefits, Risks, and Expectations

How do dietary supplements affect health? *

Please select only one of the following answers:

• Dietary supplements are always beneficial for health

• Dietary supplements can have a positive effect on health

• Dietary supplements have no effect on health

• Dietary supplements can be harmful to health

• Dietary supplements are always harmful to health

• Don't know

Please evaluate the following statements: *

Select the appropriate answer for each item:

Applies, Rather applies, Rather does not apply, Does not apply, Don't know

Taking dietary supplements during times of physical stress (e.g., pregnancy, child development, illness, stress) promotes health

Dietary supplements are particularly beneficial to the health of vegans

Dietary supplements are beneficial to the health of pregnant women

Dietary supplements have a negative effect on the health of the mother and child during pregnancy

Pregnant vegans should take dietary supplements more than non-vegan pregnant women

Vegan-fed children should not take dietary supplements

Vegan-fed children should take dietary supplements more than children with a mixed diet

Dietary Supplements: Personal Selection Criteria

Please answer the following questions with yes or no: *

Do you take dietary supplements?

- Yes
- No

Have you ever received advice on dietary supplements?

- Yes
- No

Should physicians, pharmacists, and dietitians provide more guidance on dietary supplements for pregnant vegans?

- Yes
- No

Should physicians, pharmacists, and dietitians provide more guidance on dietary supplements for vegan-fed children?

- Yes
- No

Do you support the use of dietary supplements?

- Yes
- No

Do you support the use of dietary supplements (e.g., multivitamins, combination products, individual nutrients) during pregnancy?

• Yes

• No

Do you support the use of dietary supplements (e.g., multivitamins, combination products, individual nutrients) in early childhood (ages 0–5)?

• Yes

• No

What are your main reasons for taking dietary supplements? (Rank up to 5 answers) *

Answer only if you answered 'yes' to taking supplements. Please rank up to 5 different answers.

• Dietary supplements support health

• Essential nutrients lacking in a vegan diet can be supplemented

• My physician advised me to take supplements

• My pharmacist advised me to take supplements

• My dietitian advised me to take supplements

• My friends, family, or colleagues advised me to take supplements

• Motivated by the internet and social media (Instagram, Facebook, etc.)

• Motivated by advertising

• Motivated by scientific literature and journals

• Motivated by magazines, newspapers, and/or lifestyle magazines

• Other

Why do you reject dietary supplements during pregnancy? (Multiple answers possible) *

Only answer this question if you previously stated that you do not support the use of dietary supplements during pregnancy.

• Supplements are poorly absorbed by the body

• All essential nutrients can be obtained from a vegan diet

• Supplements harm the health of the pregnant person

• Supplements harm the health of the unborn child

• Uncertain if supplements are vegan

• Physician advised against taking them

• Pharmacist advised against taking them

• Dietitian advised against taking them

• Friends, family, or colleagues advised against taking them

• Other

Why do you reject dietary supplements for children (ages 0–5)? (Multiple answers possible) *

Only answer this question if you previously stated that you do not support the use of dietary supplements in early childhood (ages 0–5).

• Supplements are poorly absorbed by children

• All essential nutrients can be obtained from a vegan diet

• Supplements harm the health of the child

• Uncertain if supplements are vegan

• Physician advised against giving them to my child

• Pharmacist advised against giving them to my child

• Dietitian advised against giving them to my child

• Friends, family, or colleagues advised against giving them to my child

• Other

Why do you support the use of dietary supplements during pregnancy? (Multiple answers possible) *

Only answer this question if you previously stated that you support the use of dietary supplements during pregnancy.

• Supplements support the positive development of the child

• Supplements support the health of the pregnant person

• Essential nutrients missing from the vegan diet can be supplemented

• Supplements meet the increased nutritional needs during pregnancy

• Physician advised taking supplements

• Pharmacist advised taking supplements

• Dietitian advised taking supplements

• Friends, family, or colleagues advised taking supplements

• Other

Why do you support the use of dietary supplements in children (ages 0–5)? (Multiple answers possible) *

Only answer this question if you previously stated that you support the use of dietary supplements in early childhood (ages 0–5).

• Supplements support the positive development of the child

• Essential nutrients missing from the diet can be supplemented

• Physician advised giving supplements to my child

• Pharmacist advised giving supplements to my child

• Dietitian advised giving supplements to my child

• Friends, family, or colleagues advised giving supplements to my child

• Other

Do you make sure your supplements are vegan? *

Only answer this question if you previously stated that you support the use of dietary supplements.

• Yes

• No

• I don't take dietary supplements

Which dietary supplements do you take? (Multiple answers possible) *

Only answer this question if you previously answered ‘yes’ to supporting the use of dietary supplements.

• Vitamin B12

• Folic acid

• Omega-3 fatty acids (DHA/EPA)

• Iron

• Vitamin D

• Iodine

• Zinc

• Calcium

• Magnesium

• Combination products (e.g., multivitamins)

• None

• Other

Which dietary supplements did you take or are you taking during your pregnancy/pregnancies? (Multiple answers possible) *

Only answer this question if you identified as female or diverse, and are currently pregnant or have experienced one or more pregnancies.

• Vitamin B12

• Folic acid

• Omega-3 fatty acids (DHA/EPA)

• Iron

• Vitamin D

• Iodine

• Zinc

• Calcium

• Magnesium

• Combination products (e.g., prenatal multivitamins)

• None

• Other

Which dietary supplements do/did you administer to your children (ages 0–5)? (Multiple answers possible) *

Only answer this question if you previously answered 'Yes' to having children.

• Vitamin B12

• Folic acid

• Omega-3 fatty acids (DHA/EPA)

• Iron

• Vitamin D

• Iodine

• Zinc

• Calcium

• Magnesium

• Combination products (e.g., children's multivitamins)

• None

• Other

Dietary Supplements: Knowledge Level

Please answer the following questions by selecting the appropriate response: *

Options: Applies / Rather applies / Rather does not apply / Does not apply / Don't know

• I am well informed about dietary supplements for vegan-fed children

• I am well informed about dietary supplements for pregnant vegans

Which dietary supplements can be safely administered to children from infancy without consulting a physician, pharmacist, and/or dietitian? (Multiple answers possible) *

• Vitamin B12

• Folic acid

• Omega-3 fatty acids (DHA/EPA)

• Iron

• Vitamin D

• Iodine

• Zinc

• Calcium

• Magnesium

• Combination products (e.g., multivitamins for children)

• None

• Other

Which dietary supplements can pregnant vegans safely take without consulting a physician, pharmacist, and/or dietitian? (Multiple answers possible) *

• Vitamin B12

• Folic acid

• Omega-3 fatty acids (DHA/EPA)

• Iron

• Vitamin D

• Iodine

• Zinc

• Calcium

• Magnesium

• Combination products (e.g., multivitamins for children)

• None

• Other

Which dietary supplements were recommended to you during your pregnancy/pregnancies by professionals (physician, pharmacist, and/or dietitian)? (Multiple answers possible) *

Only answer this question if you identified as diverse or female, and are currently pregnant or have experienced one or more pregnancies.

• Vitamin B12

• Folic acid

• Omega-3 fatty acids (DHA/EPA)

• Iron

• Vitamin D

• Iodine

• Zinc

• Calcium

• Magnesium

• Combination products (e.g., multivitamins for children)

• None

• Other

• Advice was not received

Can pregnant vegans meet their nutrient needs through a purely plant-based diet? *

• Yes

• No

• Don't know

Can potential nutrient deficiencies in a vegan pregnancy be compensated by supplements? *

• Yes

• No

• Don't know

Can the nutrient needs of vegan-fed children aged 0–5 be met through a purely plant-based diet? *

• Yes

• No

• Don't know

Can nutrient deficiencies in vegan-fed children aged 0–5 be compensated by supplements? *

• Yes

• No

• Don't know

Dietary Supplements: Information Sources

Where do you get your information on supplement use during pregnancy? (Multiple answers possible) *

• Friends, family, colleagues

• General practitioner

• Pediatrician

• Gynecologist

• Pharmacist

• Dietitian

• Midwife

• Other vegans

• Physiotherapist

• Fitness trainer

• Vegan organizations

• Scientific literature/journals

• Internet and social media (Instagram, Facebook etc.)

• Newspapers, magazines and/or lifestyle magazines

• I do not seek information about supplements in pregnancy

• Advertising

• Other

Where do you get your information on supplement use for vegan-fed children (aged 0–5)? (Multiple answers possible) *

• Friends, family, colleagues

• General practitioner

• Pediatrician

- Gynecologist
- Pharmacist
- Dietitian
- Midwife
- Other vegans
- Physiotherapist
- Fitness trainer
- Vegan organizations
- Scientific literature/journals
- Internet and social media (Instagram, Facebook etc.)
- Newspapers, magazines and/or lifestyle magazines
- I do not seek information about supplements in pregnancy
- I do not seek information about supplements for children
- Advertising
- Other

Where is the best place to get advice on dietary supplements during pregnancy? (Rank up to 3 answers) *

- Friends, family, colleagues
- General practitioner
- Pediatrician
- Gynecologist
- Pharmacist
- Dietitian
- Midwife
- Other vegans

- Physiotherapist
- Fitness trainer
- Vegan organizations
- Scientific literature/journals
- Internet and social media (Instagram, Facebook etc.)
- Newspapers, magazines and/or lifestyle magazines
- I do not seek information about supplements in pregnancy
- Advertising
- Other

Where is the best place to get advice on dietary supplements in early childhood? (Rank up to 3 answers) *

- Friends, family, colleagues
- General practitioner
- Pediatrician
- Gynecologist
- Pharmacist
- Dietitian
- Midwife
- Other vegans
- Physiotherapist
- Fitness trainer
- Vegan organizations
- Scientific literature/journals
- Internet and social media (Instagram, Facebook etc.)
- Newspapers, magazines and/or lifestyle magazines

• I do not seek information about supplements in pregnancy

• Advertising

• Other

How would you describe the information you have received about dietary supplements? *

• Trustworthy

• Helpful

• Unclear

• Not very informative

• Insufficient

• I do not receive information about supplements

Is it easy to access information on dietary supplements for pregnant vegans? *

• Yes

• No

• Don't know

Is it easy to access information on dietary supplements for vegan-fed children (aged 0–5)? *

• Yes

• No

• Don't know

How do you evaluate the available advice/information on dosage of dietary supplements during pregnancy? *

• Very good

• Good

• Adequate

• Inadequate

• Don't know

How do you evaluate the available advice/information on dosage of dietary supplements in early childhood (ages 0–5)? *

• Very good

• Good

• Adequate

• Inadequate

• Don't know

Vegan Diet: Influencing Factors

What caused you the most uncertainty about maintaining a vegan diet during your pregnancy/pregnancies? (Multiple answers possible, max 3) *

Only answer this question if you identified as diverse or female, and are currently pregnant or have been pregnant once or multiple times.

• Social environment (family, friends, colleagues)

• Treating physicians

• Advice from pharmacist

• Dietitian

• Midwife

• Internet and social media

• Scientific literature/journals

• Newspapers, magazines, and/or lifestyle magazines

• General lack of trustworthy information on vegan diet and supplements during pregnancy

• I was not uncertain

• Other

Did you inform your gynecologist during pregnancy that you follow a vegan diet? *

Only answer this question if you identified as female or diverse, and are currently pregnant or have had one or more previous pregnancies.

• Yes

• No

Did your gynecologist advise you on dietary supplements during pregnancy? *

Only answer this question if you identified as female or diverse, and are currently pregnant or have experienced one or more pregnancies in the past.

• Yes, I received comprehensive advice

• I received minimal advice

• I did not want to be advised

• No, I received no advice

How does/do your child/children eat? *

Please answer this question only if the following condition is met:

You answered 'Yes' to the question (Do you have children?)

Select all applicable options:

• My child/children follow/s a strictly plant-based diet

• My child/children also consume/s dairy products, eggs, and honey

• My child/children also eat/s fish

• My child/children also eat/s meat

Why do you not feed your child/children exclusively a vegan diet? (Multiple answers possible) *

Please answer this question only if the following condition is met:

You answered 'Yes' to the question 'Do you have children?' and selected 'My child/children also eat/s meat', 'also eat/s fish', or 'also consume/s dairy products, eggs, and honey' in question 'How does/do your child/children eat?'

Select all applicable options:

• Family, friends, and colleagues advised against it

• General practitioner advised against it

• Pediatrician advised against it

• Pharmacist advised against it

• Dietitian advised against it

• I am concerned that a vegan diet could negatively affect my child's development

• I cannot access reliable information that explains a balanced vegan diet for children

• I cannot access reliable information regarding proper dosage and form (drops, capsules, tablets, liquid, etc.) of supplements for children

• Advice on social media (Instagram, Facebook, etc.) advised against it

• Professional literature/journals advised against it

• Newspapers, magazines, and/or lifestyle magazines advised against it

• My child requests non-vegan food

• Other

What has made you most uncertain about feeding your child/children a vegan diet? *

Please answer this question only if the following condition is met:

You answered 'Yes' to the question 'Do you have children?'

Select all applicable options (please select up to 3):

- Social environment (family, friends, colleagues)
- Attending physician
- Advice from pharmacy
- Dietitian
- General lack of trustworthy information on balanced vegan diets for children
- General lack of trustworthy information regarding proper dosage and form of supplements for children
- Professional literature/journals
- Internet and social media (Instagram, Facebook, etc.)
- Newspapers, magazines, and/or lifestyle magazines
- I have not felt uncertain
- Other

Have you informed your pediatrician that your child/children follow a vegan diet? *

Please answer this question only if the following condition is met:

You answered 'Yes' to the question 'Do you have children?' and 'My child/children follow/s a strictly plant-based diet'

Select one of the following answers:

- Yes
- No
- I do not exclusively feed my child a vegan diet

Were you advised by your pediatrician on supplement use for children? *

Please answer this question only if the following condition is met:

You answered 'Yes' to the question 'Do you have children?'

Select one of the following answers:

• Yes, I received comprehensive advice

• I received little advice

• I did not want to be advised

• No, I did not receive any advice

Dietary Supplements: Deficiency and Overdosage Risks

Please assess the health risks of nutrient deficiency (underdosing) and overdosage due to a vegan diet during pregnancy and in infants/children (up to age 5). Use the following scale for each item:

1 = No Risk, 5 = High Risk, 'Don't know' = If you are unsure.

*Risk Assessment **– vegan pregnancy***

For each nutrient listed, please indicate:

- The perceived risk if there is a deficiency or underdosage.

- The perceived risk if there is an overdosage.

- Vitamin B12:

Risk of Deficiency/Underdosing: 1 2 3 4 5 Don't know

Risk of Overdosage: 1 2 3 4 5 Don't know

- Folic Acid:

Risk of Deficiency/Underdosing: 1 2 3 4 5 Don't know

Risk of Overdosage: 1 2 3 4 5 Don't know

- Omega-3 Fatty Acids (DHA/EPA):

Risk of Deficiency/Underdosing: 1 2 3 4 5 Don't know

Risk of Overdosage: 1 2 3 4 5 Don't know

- Iron:

Risk of Deficiency/Underdosing: 1 2 3 4 5 Don't know

Risk of Overdosage: 1 2 3 4 5 Don't know

- Vitamin D:

Risk of Deficiency/Underdosing: 1 2 3 4 5 Don't know

Risk of Overdosage: 1 2 3 4 5 Don't know

- Iodine:

Risk of Deficiency/Underdosing: 1 2 3 4 5 Don't know

Risk of Overdosage: 1 2 3 4 5 Don't know

- Zinc:

Risk of Deficiency/Underdosing: 1 2 3 4 5 Don't know

Risk of Overdosage: 1 2 3 4 5 Don't know

- Calcium:

Risk of Deficiency/Underdosing: 1 2 3 4 5 Don't know

Risk of Overdosage: 1 2 3 4 5 Don't know

- Magnesium:

Risk of Deficiency/Underdosing: 1 2 3 4 5 Don't know

Risk of Overdosage: 1 2 3 4 5 Don't know

Please assess the health risks of nutrient deficiency (underdosing) and overdosage due to a vegan diet in infants/children (up to age 5). Use the following scale for each item:

1 = No Risk, 5 = High Risk, 'Don't know' = If you are unsure.

*Risk Assessment – **Infants and Children (0–5 years)***

For each nutrient listed, please indicate:

- The perceived risk if there is a deficiency or underdosage.

- The perceived risk if there is an overdosage.

- Vitamin B12:

Risk of Deficiency/Underdosing: 1 2 3 4 5 Don't know

Risk of Overdosage: 1 2 3 4 5 Don't know

- Folic Acid:

Risk of Deficiency/Underdosing: 1 2 3 4 5 Don't know

Risk of Overdosage: 1 2 3 4 5 Don't know

- Omega-3 Fatty Acids (DHA/EPA):

Risk of Deficiency/Underdosing: 1 2 3 4 5 Don't know

Risk of Overdosage: 1 2 3 4 5 Don't know

- Iron:

Risk of Deficiency/Underdosing: 1 2 3 4 5 Don't know

Risk of Overdosage: 1 2 3 4 5 Don't know

- Vitamin D:

Risk of Deficiency/Underdosing: 1 2 3 4 5 Don't know

Risk of Overdosage: 1 2 3 4 5 Don't know

- Iodine:

Risk of Deficiency/Underdosing: 1 2 3 4 5 Don't know

Risk of Overdosage: 1 2 3 4 5 Don't know

- Zinc:

Risk of Deficiency/Underdosing: 1 2 3 4 5 Don't know

Risk of Overdosage: 1 2 3 4 5 Don't know

- Calcium:

Risk of Deficiency/Underdosing: 1 2 3 4 5 Don't know

Risk of Overdosage: 1 2 3 4 5 Don't know

- Magnesium:

Risk of Deficiency/Underdosing: 1 2 3 4 5 Don't know

Risk of Overdosage: 1 2 3 4 5 Don't know

Dietary Supplements: Administration and Compliance

Main concern during supplement intake in pregnancy

Only answer this question if you are female or diverse and are currently pregnant or have been pregnant at least once before. Please select only one of the following answers:

• My unborn child could be harmed due to incorrect dosage

• My unborn child and I could be negatively affected by the intake of supplements

• My body is more burdened than supported by the supplement

• The health of my unborn child could be negatively affected by taking supplements (even with correct dosage)

• I have no concerns as supplements are safe

• Other

Main concern with giving supplements to your child(ren) aged 0-5

Please answer this question only if you answered 'Yes' to question 'Do you have children?'.

Please select only one of the following answers:

• My child could be harmed due to incorrect dosage

• My child could be negatively affected by supplements (even with correct dosage)

• I have no concerns as supplements are safe

• Other

Which dosage forms are most suitable for children? (Multiple answers possible)

Please select all applicable answers:

• Drops

• Liquid/Syrup

• Whole tablet

• Tablet crushed to powder (dissolved in water, juice, yogurt, etc.)

• Whole capsule

• Capsule contents emptied (dissolved in water, juice, yogurt, etc.)

• Supplements in the form of 'sweets' (e.g., gummy drops, toffees)

• Toothpaste enriched with nutrients (e.g., Vitamin B12)

• Mouth spray (e.g., with Vitamin D, Vitamin B12)

•None

Supplement use in pregnancy and with children

Please answer this question only if you answered 'Yes' to question 'Do you have children?'.

Please select the appropriate answer for each statement:

• I take/took supplements during pregnancy: Always / Mostly / Rarely / Never / No answer

• I adiminister/ed supplements to my children: Always / Mostly / Rarely / Never / No answer

• I follow/followed the recommended dosage when giving supplements to my children: Always / Mostly / Rarely / Never / No answer

• Giving supplements (drops, tablets, capsules, liquids, etc.) to my children is/was difficult: Always / Mostly / Rarely / Never / No answer

• I am/was uncertain when giving supplements to my children: Always / Mostly / Rarely / Never / No answer

At what age did you start giving supplements to your child?

Only answer this question if you have one child who follows a strictly plant-based (vegan) diet and you indicated that you rarely, mostly, or always administered them dietary supplements.

• From the start of solid foods

• After one month

• After 1-3 months

• After 3-6 months

• After 6-12 months

• After 12-24 months

• Later

At what age did you start giving supplements to your children? (Multiple answers possible)

Only answer this question if you have two or three children who follow a purely plant-based (vegan) diet and you indicated that you always, mostly, or rarely administer them dietary supplements.

• From birth

• From the start of solid foods

• After one month

• After 1-3 months

• After 6-12 months

• After 12-24 months

• Later

Please rate the following statements

• Regular intake of supplements during pregnancy is important for their effectiveness: Yes / Rather Yes / Rather No / No / Don't know

• Regular intake of supplements in early childhood (0–5 years) is important for their effectiveness: Yes / Rather Yes / Rather No / No / Don't know

• Clear dosage recommendations are a prerequisite for me to take supplements during pregnancy as a vegan: Yes / Rather Yes / Rather No / No / Don't know

• Clear dosage recommendations are a prerequisite for me to administer supplements to vegan-fed children (aged 0–5): Yes / Rather Yes / Rather No / No / Don't know

How do you rate giving supplements to your child(ren) aged 0–5?

Answer only if you answered 'Yes' to question ‘Do you have children?’.

• Drops: Uncomplicated / Rather uncomplicated / Rather complicated / Complicated / Don’t know

• Whole tablet: Uncomplicated / Rather uncomplicated / Rather complicated / Complicated / Don’t know

• Tablet as powder (in water, juice, yogurt): Uncomplicated / Rather uncomplicated / Rather complicated / Complicated / Don’t know

• Whole capsule: Uncomplicated / Rather uncomplicated / Rather complicated / Complicated / Don’t know

• Capsule contents emptied: Uncomplicated / Rather uncomplicated / Rather complicated / Complicated / Don’t know

• Supplements as 'sweets': Uncomplicated / Rather uncomplicated / Rather complicated / Complicated / Don’t know

• Enriched toothpaste: Uncomplicated / Rather uncomplicated / Rather complicated / Complicated / Don’t know

• Mouth spray: Uncomplicated / Rather uncomplicated / Rather complicated / Complicated / Don’t know

Thank you for participating in the survey! If you have any questions, please contact Wolfgang Huber-Schneider (study coordinator): a00225229@unet.univie.ac.at
