# Supplementary material for: Can Dietary Supplements Be Linked to a Vegan Diet and Health Risk Modulation During Vegan Pregnancy, Infancy, and Early Childhood? The VedieS Study Protocol for an Explorative, Quantitative, Cross-Sectional Study
Source: Int J Environ Res Public Health. 2025 Jul 31;22(8):1210. doi: 10.3390/ijerph22081210 (PMC12386434; doi:10.3390/ijerph22081210)
Supplement: Supplementary file 1 [file ijerph-22-01210-s001.zip › S5_3677589.pdf]

Note to the reviewers:

Some questions in this survey are conditional, meaning they are only displayed if a previous answer logically leads to them. In both the German and English versions of the questionnaire, these conditions are indicated in italic text directly below the relevant question. Survey participants will not see or need to read these notes - they are automatically guided through the questionnaire based on their prior responses. Additionally, the section headings for the different test categories (highlighted in blue) are not visible to participants.

Please also note that the attached versions of the questionnaire are intended for illustrative purposes only. In the actual online survey, the structure is more intuitive and user-friendly, with dynamic filtering that ensures clarity and logic. This level of interactivity and visual clarity cannot be fully replicated in the static PDF format.

# Expert Survey on a Vegan Diet and the Use of Dietary Supplements During Pregnancy, Infancy and Early Childhood

## Participant Information and Informed Consent for Study Participation

### Working Title of the Study:

*Can dietary supplements be linked to a vegan diet and health risk modulation during pregnancy, infancy, and early childhood?*

Dear Participant,

we invite you to take part in the study mentioned above.

Your participation in this study is voluntary. You may withdraw from the survey at any time without providing any reason, and your willingness to participate will be considered revoked. Refusal to participate or early withdrawal from the study will not have any negative consequences for you. Studies of this nature are necessary to gain reliable new scientific insights. However, a prerequisite for conducting such studies is that you give your consent to participate. NO sensitive data such as your name, email address, or IP address will be collected. Completing the survey will take approximately 25 minutes of your time. Please read the following text carefully and do not hesitate to ask questions. If you have any questions, please contact the doctoral student conducting the study, Wolfgang Huber-Schneider (contact details below).

**Please confirm the informed consent only (confirmation = clicking "Next" – starting the survey is considered as consent):**

- • if you have fully understood the nature and procedure of the study,
- • if you are willing to give your consent to participate, and
- • if you are aware of your rights as a participant in this study.

## • What is the purpose of the study?

The aim of this study is to examine the relationship between counseling by medical professionals (e.g., physicians, pharmacists) and other influencing factors (e.g., social environment, social media) with a vegan diet and the intake of dietary supplements during pregnancy, infancy, and early childhood (up to the age of 5 years) among vegans. This will help identify and minimize potential health risks for vegans. Your participation will

contribute to improving information access and health risk prevention for vegans. This survey is part of a PhD project.

## How does the study proceed?

The questionnaire is intended for medical experts (gynecologists, pediatricians, general practitioners, pharmacists, dietitians). It includes questions about a vegan diet and the use of dietary supplements during pregnancy, infancy, and early childhood (up to 5 years of age). Completing the questionnaire will take approximately 20 minutes.

## • What are the benefits of participating in the study?

You are not expected to receive any direct personal benefits from participating. However, by answering the questionnaire, you are contributing to the identification and prevention of potential health risks for pregnant vegans and vegan-fed children. From a scientific perspective, the study aims to optimize the quality and accessibility of information for vegans by investigating the sources of information and their influence on diet and supplement intake.

## • Are there any risks or side effects associated with participating?

No risks or discomforts are expected from participating in the study.

## Inclusion Criteria for Study Participation:

1. Gynecologists, pediatricians, general practitioners, pharmacists, dietitians with or without focus on a vegan diet.

2. Confirmation of study information and informed consent.

## Exclusion Criteria for Study Participation:

1. Other medical professionals not listed in the inclusion criteria.

2. No confirmation of study information and informed consent.

To participate, both inclusion criteria must be fulfilled. Participation is not possible if even one exclusion criterion applies.

## • Does participation affect daily life or involve any obligations?

No, you may participate in the survey at any time and withdraw at any point without consequences or other effects.

## • What should be done if symptoms, side effects, or injuries occur?

As this is a questionnaire-based survey, no symptoms or adverse effects are expected.

## • When can the study be terminated prematurely?

You may withdraw your consent and discontinue participation at any time without giving reasons and without any disadvantages resulting from this decision.

## • How will the data collected in this study be used?

No personal data is required to participate in the survey. Your responses will be completely anonymous. The collected anonymous data will be stored solely for statistical purposes. No personal data (e.g., name, IP address, email address) will be collected or stored. It will not be possible to trace any data back to your identity.

## • Are there any costs for participants? Will there be reimbursement or compensation?

There are no costs associated with participating in this study. No reimbursement or compensation will be provided for participating in the survey/study.

## • Possibility to ask further questions

If you have further questions regarding the study, please contact Wolfgang Huber-Schneider. Questions related to your rights as a participant will also be answered. Once general results of the study are available, you can be informed upon request.

## • Contact – Study Team / Doctoral Student

The survey is part of the dissertation of Wolfgang Huber-Schneider at the Department of Nutritional Sciences, University of Vienna, in cooperation with AGES (Austrian Agency for Health and Food Safety). For questions, please contact:

Doctoral Student: Mag. pharm. Wolfgang Huber-Schneider
Email: a00225229@unet.univie.ac.at

Study Leadership:
- Univ.-Prof. Mag. Dr. Karl-Heinz Wagner (Department of Nutritional Sciences, University of Vienna)
- Univ.-Doz.in Mag.a Dr.in Ingrid Kiefer (AGES)
- Mag. pharm. Wolfgang Huber-Schneider (Doctoral Student)

**By clicking “Next,” you automatically consent to participate in the survey.**

This survey contains 54 questions.

## General/Demographics

**Gender** *
*Please choose one of the following answers:*
• Female
• Male
• Diverse

**Age (in years)** *
*Please choose one of the following answers:*
• Under 30
• 31-40
• 41-50
• 51-60
• 61 or older

**In which country do you work?** *
*Please choose one of the following answers:*
• Austria
• Germany
• Switzerland
• Italy
• Other EU country
• Other

**Where is your workplace located (please enter the ZIP code)?** *
*Please enter your answer here:*

**Which expert group do you belong to?** *
*Please choose one of the following answers:*
• Specialist in gynecology and obstetrics
• Specialist in pediatrics
• Specialist in general medicine

- Pharmacist
- Dietitian

**I am ***

*(Answer this question only if the following conditions are met:*
*Answer was 'Austria' for the question 'In which country do you work?' and Answer was '(Specialist) in general medicine' or 'Specialist in pediatrics' or 'Specialist in gynecology and obstetrics' for the question 'Which expert group do you belong to?')*
*Please choose one of the following answers:*

- Contracted physician
- Private physician
- Hospital-based physician only
- Other

## Health

**What does health mean to you personally? (Rank up to 3 answers) ***

*All your answers must be different and must be assigned.*
*Please select up to 3 answers.*
*Please number each box in your preferred order, from 1 to 8:*

- A state of complete mental, physical, and social well-being
- Freedom from illness and infirmity
- Feeling well – even without diagnoses or test results
- Not suffering from a diagnosed illness
- Being able to live everyday life without limitations
- Being mentally and physically resilient
- Being happy and free from complaints
- Being above-average in performance

**What does health during pregnancy mainly mean to you? ***

*Please choose one of the following answers:*

- A pregnancy free of complications
- A pregnancy in which the child develops ideally due to the provision of all necessary nutrients
- A pregnancy in which the child meets developmental milestones (according to medical exams) as best as possible
- A pregnancy where one feels well – even without medical confirmation of health status
- A pregnancy that proceeds as expected and on schedule

**What does health in childhood mainly mean to you? ***

*Please choose one of the following answers:*

• The child makes physical and mental progress appropriate for their age (according to doctors, educators, etc.)
• The child makes physical and mental progress that I consider appropriate
• The child is not prone to infections and has a strong immune system
• The child appears happy and well-balanced based on my observation
• The child makes above-average physical and mental progress

## Vegan Diet: Definition and Counseling

**How do you eat? ***

*Please choose one of the following answers:*

• Vegan (plant-based only)
• Vegetarian (no fish/meat)
• Pescetarian (no meat, but fish and/or seafood)
• Omnivore (mixed diet including fish, seafood, and meat)

**Do you deal with vegans in your professional practice? ***

*Please choose one of the following answers:*

• Yes
• No
• Don't know

**Do you offer counseling on dietary supplements as part of a vegan diet? ***

*Please choose one of the following answers:*

• Yes
• No

**Is a vegan diet healthy? ***

*Please choose one of the following answers:*

• Yes, always
• Yes, but only if vegans are fully informed about a balanced, plant-based diet
• No
• Don't know

**Why do you eat vegan? (Multiple answers possible) ***
*Answer this question only if the previous answer was 'Vegan (plant-based only)'*
*Please select all applicable answers:*
• For health reasons
• For animal welfare reasons
• For climate protection reasons
• Because I do not like the taste of animal products
• Other

**How long have you been eating vegan? ***
*Answer this question only if the previous answer was 'Vegan (plant-based only)'*
*Please choose one of the following answers:*
• Less than one month
• About 3 months
• About 6 months
• About 1 year
• About 1–3 years
• About 3–5 years
• About 5–10 years
• More than 10 years

**Please evaluate the following statements: ***

*Please select the appropriate answer for each item:*

*Options: strongly agree, rather agree, rather disagree, strongly disagree*

• I advise all vegans against following a vegan diet

• I advise vegans against maintaining a vegan diet during pregnancy

• I advise against raising infants and children on a vegan diet

**Please evaluate the following statements: ***

*Please select the appropriate answer for each item:*

*Options: often, rarely, never, don't know*

• I counsel vegans in my daily professional practice

• I counsel pregnant vegans in my daily professional practice

• I counsel parents of vegan-fed children in my daily professional practice

## Dietary Supplements: Definition and Understanding

**How do dietary supplements work? ***

*Please choose one of the following answers:*

• Like prescription drugs

• Like over-the-counter drugs

• Like food

• Like placebos

• Cannot be compared with any of the above options

• Don't know

## Dietary Supplements: Benefits, Risks, and Expectations

**How do medically/pharmaceutically/dietetically recommended and properly dosed dietary supplements affect health? ***

*Please choose one of the following answers:*

• Always beneficial for health

• Can have a positive effect on health

• Have no impact on health

• Can harm health

• Always pose a health risk

• Don't know

**Please evaluate the following statements: ***

*Select the appropriate response for each item:*

*Options: strongly agree, rather agree, rather disagree, strongly disagree, don't know*

• Taking dietary supplements during periods of physical stress (e.g., pregnancy, child development, illness, stress, etc.) is health-promoting

• Taking dietary supplements is especially beneficial for vegans

• Dietary supplements have a negative effect on the health of mother and child during pregnancy

• Pregnant vegans should take dietary supplements more than non-vegan pregnant women

• Vegan-fed children should not take dietary supplements

• Vegan-fed children should take dietary supplements more than children on a mixed diet

**Please answer the following questions by selecting the appropriate answer: ***

*Options: yes, rather yes, rather no, no, don't know*

• Is a vegan diet during pregnancy a health risk for mother and child?

• Do dietary supplements help reduce risks in a vegan pregnancy?

• Is a vegan diet for infants and children a health risk?

• Do dietary supplements help reduce nutrient deficiency risks in vegan-fed infants and children?

• Is it medically necessary to supplement dietary supplements during pregnancy?

## Dietary Supplements: Recommendations

**Please answer the following questions with yes or no: ***

• Do you personally take dietary supplements?

• Do you support the use of dietary supplements during pregnancy (regardless of diet)?

• Do you support the use of dietary supplements for children aged 0–5 (regardless of diet)?

• Do you recommend the use of dietary supplements for pregnant women with a mixed diet?

• Do you recommend the use of dietary supplements for children aged 0–5 with a mixed diet?

• Do you recommend the use of dietary supplements for pregnant vegan women?

• Do you recommend the use of dietary supplements for vegan-fed children aged 0–5?

**Why do you NOT recommend taking dietary supplements during pregnancy for women on a mixed diet? ***

*Answer this question only if the following condition is met:*

*Answer was 'No' to: 'Do you recommend the use of dietary supplements during pregnancy for women on a mixed diet?'*

*Select all applicable options:*

• Dietary supplements may be poorly absorbed by the pregnant body

• All necessary nutrients can be obtained through food

• Dietary supplements harm health

• Dietary supplements have no significant effect during pregnancy

• Risk of incorrect dosage during pregnancy is too high

• Other

**Why do you NOT recommend taking dietary supplements during pregnancy for vegan women? ***

*Answer this question only if the following condition is met:*

*Answer was 'No' to: 'Do you recommend the use of dietary supplements during pregnancy for vegan women?'*

*Select all applicable options:*

• Dietary supplements may be poorly absorbed by the pregnant body

• All necessary nutrients can be obtained through a vegan diet

• Dietary supplements harm the health of the pregnant woman

• Dietary supplements have no significant effect during pregnancy

• Risk of incorrect dosage during pregnancy is too high

• Other

**Why do you NOT recommend dietary supplements for omnivorous children aged 0–5? ***

*Answer this question only if the following condition is met:*

*Answer was 'No' to: 'Do you recommend the use of dietary supplements for children aged 0–5 who are omnivorous?'*

*Select all applicable options:*

• Dietary supplements may be poorly absorbed by the child's body

• All necessary nutrients can be obtained through food

• Dietary supplements harm health

• Dietary supplements have no significant effect on children

• Risk of incorrect dosage for children aged 0–5 is too high

• Other

**Why do you NOT recommend dietary supplements for vegan-fed children aged 0–5? ***

*Answer this question only if the following condition is met:*

*Answer was 'No' to: 'Do you recommend the use of dietary supplements for children aged 0–5 who are vegan?'*

*Select all applicable options:*

• Dietary supplements may be poorly absorbed by the child's body

• All necessary nutrients can be obtained through a vegan diet

• Dietary supplements harm health

• Dietary supplements have no significant effect on children

• Risk of incorrect dosage for children aged 0–5 is too high

• Other

**Why do you recommend dietary supplements during pregnancy for women on a mixed diet? ***

*Answer this question only if the following condition is met:*

*Answer was 'Yes' to: 'Do you recommend the use of dietary supplements during pregnancy for women on a mixed diet?'*

*Please choose one of the following answers:*

• Supplements support the healthy development of the unborn child

• Supplements support the health of the pregnant woman

• Supplements support both the healthy development of the unborn child and the health of the pregnant woman

• Essential nutrients that are not sufficiently provided by an omnivorous diet should be supplemented to minimize risks during pregnancy

• Other

**Why do you recommend dietary supplements during pregnancy for vegan women? ***

*Answer this question only if the following condition is met:*

*Answer was 'Yes' to: 'Do you recommend the use of dietary supplements during pregnancy for vegan women?'*

*Please choose one of the following answers:*

• Supplements support the healthy development of the unborn child

• Supplements support the health of the pregnant vegan woman

• Supplements support both the healthy development of the unborn child and the health of the pregnant vegan woman

• Essential nutrients that are not sufficiently provided by a vegan diet should be supplemented to minimize risks during pregnancy

• Other

**Why do you recommend dietary supplements for omnivorous children aged 0–5? ***

*Answer this question only if the following condition is met:*

*Answer was 'Yes' to: 'Do you recommend the use of dietary supplements for children aged 0–5 who are omnivorous?'*

*Please choose one of the following answers:*

• Supplements support the healthy development of the child

• Essential nutrients not sufficiently provided by diet can be supplemented

• Other

**Why do you recommend dietary supplements for vegan-fed children aged 0–5? ***

*Answer this question only if the following condition is met:*

*Answer was 'Yes' to: 'Do you recommend the use of dietary supplements for children aged 0–5 who are vegan?'*

*Please choose one of the following answers:*

• Supplements support the healthy development of the child

• Essential nutrients not sufficiently provided by a vegan diet can be supplemented

• Other

**Please evaluate the following statements: ***

*Options: strongly agree, rather agree, rather disagree, strongly disagree*

• I would be more likely to recommend supplements to a pregnant vegan woman than to a pregnant omnivorous woman

• I would recommend additional supplements to a pregnant vegan woman that I would not recommend to an omnivorous woman

• I would recommend supplements to a vegan child even without a diagnosed deficiency (e.g., via blood test)

• I would not generally recommend supplements for children – only if there are further indicators (e.g., blood test)

**Which dietary supplements would you recommend to a pregnant vegan woman? (Multiple answers possible) ***

• Vitamin B12

• Folic acid

• Omega-3 fatty acids (DHA/EPA)

• Iron

• Vitamin D

• Iodine

• Zinc

• Calcium

• Magnesium

• Combination supplements (e.g., prenatal multivitamins)

• None

• Other

**Which dietary supplements would you recommend to a pregnant omnivorous woman? (Multiple answers possible) ***

• Vitamin B12

• Folic acid

• Omega-3 fatty acids (DHA/EPA)

• Iron

• Vitamin D

• Iodine

• Zinc

• Calcium

• Magnesium

• Combination supplements (e.g., prenatal multivitamins)

• None

• Other

**Which dietary supplements would you recommend to a vegan child aged 0–5? (Multiple answers possible) ***

• Vitamin B12

• Folic acid

• Omega-3 fatty acids (DHA/EPA)

• Iron

• Vitamin D

• Iodine

• Zinc

• Calcium

• Magnesium

• Combination supplements (e.g., children's multivitamins)

• None

• Other

**Which dietary supplements would you recommend to an omnivorous child aged 0–5? (Multiple answers possible) ***

• Vitamin B12

- Folic acid
- Omega-3 fatty acids (DHA/EPA)
- Iron
- Vitamin D
- Iodine
- Zinc
- Calcium
- Magnesium
- Combination supplements (e.g., children's multivitamins)
- None
- Other

## Dietary Supplements: Knowledge

**Are you aware that there are vegan alternatives to non-vegan supplements (e.g., vegan vitamin D3 and vegan omega-3)? ***

- Yes
- I wasn't aware of this until now

**Please evaluate the following statements: ***

*Options: strongly agree, rather agree, rather disagree, strongly disagree, don't know*

- My personal knowledge of dietary supplements is comprehensive
- My personal knowledge of vegan dietary supplements is comprehensive
- The bioavailability of dietary supplements is high
- The bioavailability of vegan supplements is equal to non-vegan ones

**Please answer the following questions: ***

*Options: yes, no, don't know*

• Can pregnant vegans meet their nutritional needs through a purely plant-based diet?

• Can potential deficiencies in a vegan pregnancy be compensated through supplements?

• Can the nutritional needs of vegan-fed children (aged 0–5) be met through a purely plant-based diet?

• Can potential deficiencies in vegan-fed children (aged 0–5) be compensated through supplements?

## Dietary Supplements: Sources of Information

**Where is the best place to get advice on supplements during pregnancy? ***

• General practitioner

• Gynecologist

• Pharmacist

• Dietitian

• Midwife

• Scientific literature and journals

• Internet

• No advice necessary

**Where is the best place to get advice on supplements during early childhood (0–5 years)? ***

• General practitioner

• Pediatrician

• Pharmacist

• Dietitian

• Scientific literature and journals

• Internet

• No advice necessary

## Dietary Supplements: Expert Influence

• Do you advise pregnant vegans on supplement use? (Yes / No /)

• Do you advise parents of vegan-fed children on supplement use? (Yes / No /)

• Do your clients/patients inform you they are vegan? (Yes / No / Sometimes)

• Do some vegan clients/patients (pregnant or parents) mention their vegan diet only later (not during the initial consultation/first contact)? (Yes / No)

**In your opinion, why might clients/patients not mention their vegan diet to medical professionals? (Multiple answers possible) ***

• They believe the topic is not worth mentioning

• They feel sufficiently informed already

• They fear criticism for their diet

• They forgot or had no specific reason

• They are skeptical of medical professionals

• Don't know

## Dietary Supplements: Administration and Compliance

**Which dosage form of supplement administration is most suitable for children? (Multiple answers possible) ***

• Drops

• Liquid/Syrup

• Whole tablet

• Crushed tablet (in juice/yogurt, etc.)

• Whole capsule

• Capsule content emptied (in juice/yogurt, etc.)

• Candy form (e.g., gummies)

• Toothpaste with nutrients (e.g., B12)

• Oral spray (e.g., with vitamin D, B12)

• None

**What is your greatest concern if pregnant vegan women take dietary supplements? ***

*Please choose one of the following answers:*

*Please select only one of the following options:*

• The unborn child is harmed due to incorrect dosage

• Mother and child experience health burdens during pregnancy due to supplement intake

• The pregnant body is more burdened than supported by supplement intake

• The health of the unborn child is affected by supplement intake (even with correct dosage)

• I have no concerns, as supplements are safe

• Other

**What is your greatest concern if pregnant vegan women do not take dietary supplements? ***

*Please choose one of the following answers:*

*Please select only one of the following options:*

• Complications during pregnancy and/or birth due to nutrient deficiencies

• Increased risk of premature birth

• Increased risk of miscarriage

• Health consequences for the mother and/or newborn due to nutrient deficiencies

• I have no concerns

• Other

**What is your greatest concern if parents administer dietary supplements to their vegan-fed children (aged 0–5)? ***

*Please choose one of the following answers:*

*Please select only one of the following options:*

• The child is harmed due to incorrect dosage

• The child is health-wise burdened by supplement intake (even with correct dosage)

• I have no concerns, as supplements are safe

• Other

**What is your greatest concern if parents do not administer dietary supplements to their vegan-fed children (aged 0–5)? ***

*Please choose one of the following answers:*

*Please select only one of the following options:*

• Health consequences due to nutrient deficiencies

• Delayed physical and/or mental development due to nutrient deficiencies

• I have no concerns

• Other

**Please rate the following: ***

*Options: high, rather high, rather low, low, don't know*

• Compliance of clients/patients regarding supplement use during pregnancy

• Compliance of vegan clients/patients regarding supplement use during pregnancy

**Please rate the following: ***

*Options: very, rather, rather not, not, don't know*

• How consistent are vegan clients/patients in supplementing their children?

• How difficult is accurate dosing (drops, tablets, capsules, etc.) by vegan parents?

**Please answer the following questions: ***

*Options: yes, no, don't know*

• Is regular intake of supplements important during pregnancy for effectiveness?

• Is regular intake of supplements important for children (0–5) for effectiveness?

**How do you rate administration of supplements to children aged 0–5? ***

*Options: easy, rather easy, rather difficult, difficult, don't know*

• Drops

• Liquid/Syrup

• Whole tablet

• Crushed tablet (in juice/yogurt, etc.)

• Whole capsule

• Capsule content emptied (in juice/yogurt, etc.)

• Candy form (e.g., gummies)

• Toothpaste with nutrients (e.g., B12)

• Oral spray (e.g., with vitamin D, B12)

Thank you for participating in the survey! For any questions, please contact Wolfgang Huber-Schneider: a00225229@unet.univie.ac.at

Submit your survey.

Thank you for completing the questionnaire.
